# Systems Identification and Characterization of Cell Wall Reassembly and Degradation Related Genes in *Glycine max* (L.) Merill, a Bioenergy Legume

**DOI:** 10.1038/s41598-017-11495-4

**Published:** 2017-09-07

**Authors:** Muhammad Amjad Nawaz, Hafiz Mamoon Rehman, Muhammad Imtiaz, Faheem Shehzad Baloch, Jeong Dong Lee, Seung Hwan Yang, Soo In Lee, Gyuhwa Chung

**Affiliations:** 1 Department of Biotechnology, Chonnam National University, Chonnam, 59626 Republic of Korea; 2 School of Environmental Science and Engineering, Guangzhou University, Guangzhou, 510275 China; 3 Department of Field Crops, Faculty of Agricultural and Natural Science, Abant Izzet Baysal University, 14280 Bolu, Turkey; 4 Division of Plant Biosciences, Kyungpook National University, Daegu, 41566 Republic of Korea; 5 Metabolic Engineering Division, Department of Agricultural Biotechnology, National Institute of Agricultural Sciences (NAS), Jeonju, 54874 Republic of Korea

## Abstract

Soybean is a promising biomass resource for generation of second-generation biofuels. Despite the utility of soybean cellulosic biomass and post-processing residues in biofuel generation, there is no comprehensive information available on cell wall loosening and degradation related gene families. In order to achieve enhanced lignocellulosic biomass with softened cell walls and reduced recalcitrance, it is important to identify genes involved in cell wall polymer loosening and degrading. Comprehensive genome-wide analysis of gene families involved in cell wall modifications is an efficient stratagem to find new candidate genes for soybean breeding for expanding biofuel industry. We report the identification of 505 genes distributed among 12 gene families related to cell wall loosening and degradation. 1262 tandem duplication events contributed towards expansion and diversification of studied gene families. We identified 687 Simple Sequence Repeat markers and 5 miRNA families distributed on 316 and 10 genes, respectively. Publically available microarray datasets were used to explore expression potential of identified genes in soybean plant developmental stages, 68 anatomical parts, abiotic and biotic stresses. Co-expression networks revealed transcriptional coordination of different gene families involved in cell wall loosening and degradation process.

## Introduction

Soybean (*Glycine max* (L.) Merrill), a model legume species, is one of the world’s main sources of protein and edible oil^1, 2^. It occupies first rank as edible oil source in the world with an estimated global production of 336.62 million metric tons by 2017^3^. Being an energy-intensive crop, energy output from soybean must be increased. The utility of soybean molasses along with post-processing residues in ethanol production has already been considered in many countries, especially in Brazil^4, 5^ and is it has been reported as a promising and viable alternative in terms of usable energy and reduced greenhouse gases as compared to many other crops^6, 7^. Such a superiority makes soybean a potential alternative resource to provide agro-industrial by-products such as molasses and residual field biomass after oil production^5^. One ton of a de-oiled soybean yields 190.8 Kg molasses, which upon fermentation can produce 18.4 Kg ethanol^8^. Such a yield can be improved by increasing amount of polymeric compounds used in biofuel generation by employing functional genomics tools and molecular biology techniques.

Recently, the focus of biofuel industry has been shifted towards second generation bioethanol to overcome the future fuel demands. Second generation biofuel production targets plant biomass which is the most abundant organic raw material^9, 10^. Most of this raw material come from grasses i.e. sugarcane, maize, and fodder sorghum. None of the current crops fulfills the demands of an ideal biofuel crop, shifting the attention of scientists towards other crops such as legumes i.e. soybean^4, 6^. However, an optimized and enough bioethanol production from soybean is yet to be achieved. The first step in the production of bioethanol is to convert the biomass into fermentable sugars. Fermentable sugars can be generated by accessing polysaccharides which form plant cell walls^11^. The lignocellulosic material of plant cell walls is mainly composed of cellulose, hemicellulose, lignin, and pectin^12^. To aid the conversion of these polysaccharides, it is important to understand the mechanisms involved in cell wall loosening, reassembly and degradation^13–15^. The assembly and range of potential cross-links between polymers, (cellulose, hemicellulose, and pectins) outlines the cell-wall architecture which enforces several restrictions on its degradability^10^.

Degradation of cell wall polymers can be achieved by various strategies such as biomass pre-treatment with chemicals and the production of less-recalcitrant cell walls *in planta* by engineering the genes involved in cell wall reassembly and degradation (CWRD). However, each method comes at a cost. Pre-treatment can interfere with macro- and microfibril associations, resulting in the removal of certain soluble hemicelluloses. On the other hand, engineering plants with less recalcitrant cell walls is an effective strategy however reduced biomass yield, decreased germination frequency with less viable seed production and the unwanted aftermaths. Whatever the case is, it should not pose any serious consequences on plant health by conflicting with principle biological activities of the cell wall. Although some negative after effects have been witnessed in some experiments^16^ but thanks to recent developments in understanding cell wall synthesis, assembly, loosening, and degradation processes, modification of cell walls has helped genetic engineers to recover the biomass yields^17–19^. Clearly, a better understanding of the processes underlying the interactions between pathogens and the cell wall will support the development of plants with optimized lignocellulosic characteristics, without negatively affecting disease resistance. However, the recent studies on over-expression of pectin modifying enzymes resulted in increased amount of released sugars^20, 21^ suggesting that it is essential to understand the mechanistic aspects underlying some natural cell-wall degrading processes. This kind of investigations could be achieved by understanding co-expression networks of gene modules related to cell wall reassembly and degradation^22, 23^. These networks will in-turn lead towards a more detailed explanation of step-wise cell wall modification and degradation events from cell separation to programmed cell death and lysis of the cell wall polymers^10, 24^. Ultimately, plants could be engineered with modified cell walls by manipulating the key players which upon activation will give swollen cell walls before harvesting. Such a biomass would be more promising raw material for bioethanol industry.

Plants share several common features in relation to cell wall disassembly and degradation. This involves co-action of several enzymes and a step-by-step procedure which starts with expansion of the cell followed by separation and then hydrolysis of cell wall polymers (particularly the hydrolysis of pectins). Some of the natural cell wall degradation processes depend on cell targeting to start carbohydrate breakdown and then continue degradation to the surrounding cells where first the hemicelluloses are degraded followed by cellulose^11^. Cell wall loosening involves expansins (EXP), yieldins and xyloglucan endotransglucosylases/hydrolases (XTH)^25–27^. Expansin proteins provoke cell-wall relaxation by destabilizing hydrogen bonds which in turn ease the action of glycoside hydrolases (GHs) on cellulose^13, 25, 28^, which further promote slippage of xyloglucans on the cellulose microfibrils surface. The endogenous production and secretion of plant GHs is sequential and it follows spatial and temporal characteristics and is always compatible with architecture of particular cell wall. This in turn recruits enzymes (expansins and pectinases) for separation and expansion and xyloglucanases and cellulases to loosen cell wall. While degradation requires a range of pectin modifying and glycoside hydrolases such as endo-1, 4-b-glucanases, endo-xylanases, glucan1, 3-b-glucosidases, polygalacturonases, b-galactosidases, pectate and pectin lyases (PLs), rhamnogalacturonan I lyases, pectin methyl esterases and pectin acetyl esterases^10, 11^. Physiological roles of plant GHs has been well reviewed elsewhere^29, 30^. Theses enzymes act accordingly in a relatively conserved sequence of events *in planta* during fruit ripening, leaf abscission, pathogen attack, under stress, aerenchyma formation and transport of cell wall storage proteins^10^. Details of mechanism of cell wall degradation for biofuel has been discussed elsewhere^10, 14^.

The competitive and simultaneous demand of food and biofuel from soybean is increasing which demands a clear and thorough understanding of mechanisms underlying cell wall degradation and loosening by the application of genomics and computation biology tools. The availability of whole genome sequence, microarrays, co-expression network platforms, proteome and transcriptome data has enabled large-scale investigations in order to understand and examine gene families involved in soybean CWRD. It will greatly help to increase the efficiency of polymer digestibility and scarification and production of less-recalcitrant *in planta* cell walls. The present study focused on mining publically available soybean genome for identification and comprehensive analysis of gene families involved in soybean cell wall loosening and degradation. Phylogenetic analysis, physical mapping of genes, duplication analysis, synteny analysis and gene co-expression analysis was done to get insight into evolution, functional relationship, and transcriptional association and coordination. To further aid future molecular breeding and biotechnological applications, all the identified genes were subjected to predict the presence of SSR markers and miRNA target sites. Furthermore, publically available microarray datasets for various soybean plant developmental stages, anatomical parts as well as under different biotic and abiotic stresses were analyzed to study the expression potential of all gene families involved in CWRD. Finally, gene co-expression networks were studied to visualize transcriptional coordination within studied gene families.

## Results

### Identification of CWRD related genes from soybean

**Figure 1:**
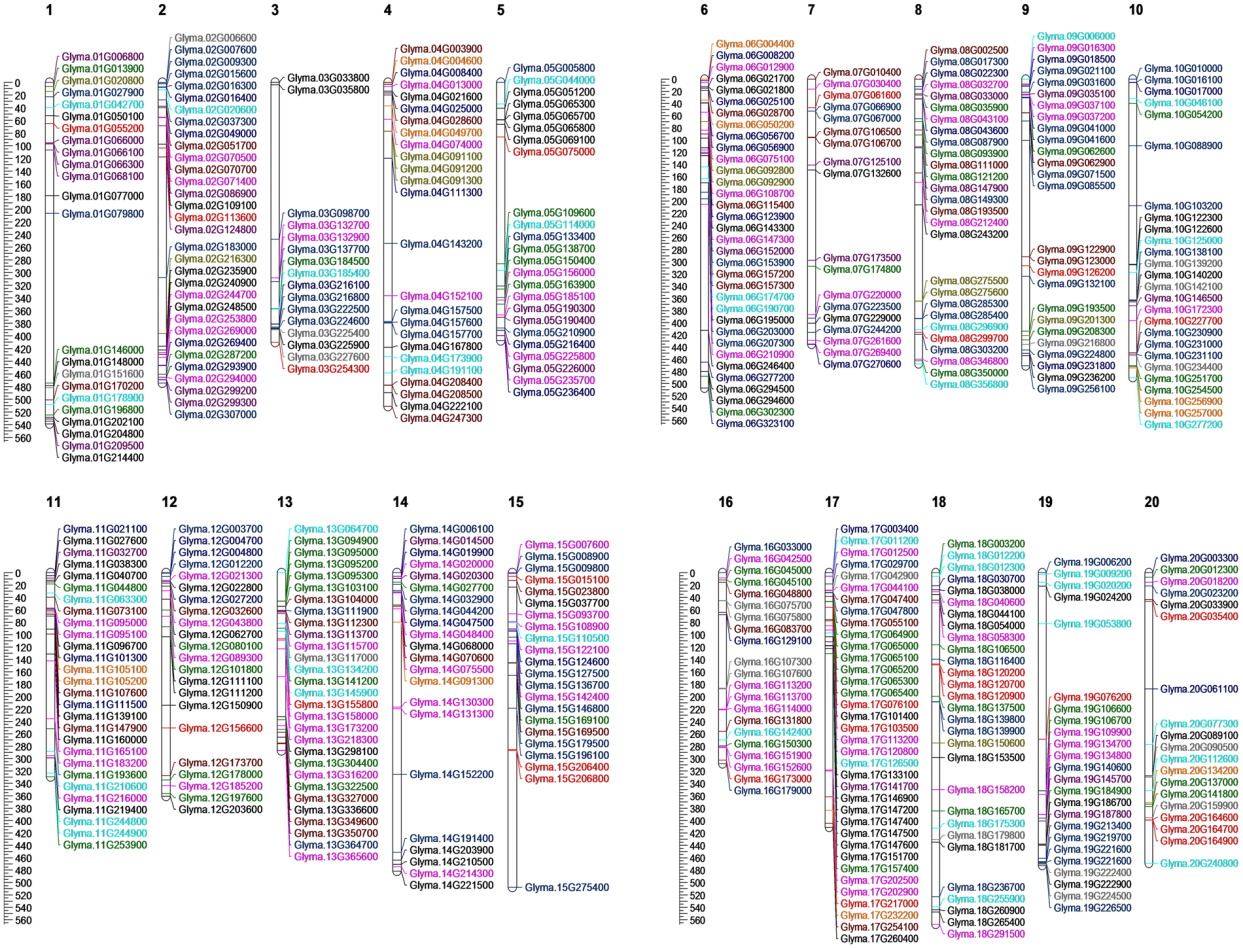
Map showing the chromosomal locations of cell wall reassembly and degradation related genes in soybean. A scale in left represents length of chromosomes in 100- kilobases. Expansins (black), Yieldins (red), Xyloglucan endotransglucosylases/hydrolases (green), Endo-1, 4-β-glucanases (dark blue), Endo-xylanases (orange), Glucan 1, 3- β-glucosidases (hot pink), Polygalacturonases (central blue), β –Galactosidases (brown), Pectate and pectin lyases (cyan), Rhamnogalacturonana l lyases (olive), Pectin methyl esterases (blue violet), and Pectin acetyl esterases (grey).

Understanding about the gene families related to CWRD in soybean can broaden the utility of soybean as a bioethanol resource. Several gene families are involved in the dynamic process of CWRD. A total of 505 genes were identified from 12 soybean CWRD related gene families (Table 1; Supplementary Table S1; Fig. 1).

**Table 1:** Summary of identified cell wall reassembly and degradation related gene families in soybean.

Gene families involved in reassembly and degradation of cell wall				
Substrate Category	Gene family	Pfam Domain(s)	CAZy ID	No. of Genes
Cell wall loosening	Expansins (EXP)	PF03330, PF01357		77
	Yeildins (YLD)	PF00704	GH18	25
	Xyloglucan endotransglucosylases/hydrolases (XTH)	PF00722, PF06955	GH16	58
Glycoside hydrolases	Endo-1, 4-β-glucanases (EGL)	PF00759	GH9	37
	Endo-xylanases (EXL)	PF00331	GH10	11
	Glucan 1, 3- β-glucosidases (GGL)	PF00332, PF07983	GH17	78
	Polygalacturonases (PGL)	PF00295, PF12708	GH28	84
	β -Galactosidases (GL)	PF01301	GH35	41
Pectin modifying	Pectate and pectin lyases	PF00544, PF04431	PL1	37
	Rhamnogalacturonana l lyases	PF06045, PF14686, PF14683	PL4	11
	Pectin methyl esterases (PME)	PF01095	CE8	19
	Pectin acetyl esterases (PAE)	PF03283	CE13	27
				505

### Cell wall loosening gene families

**Figure 2:**
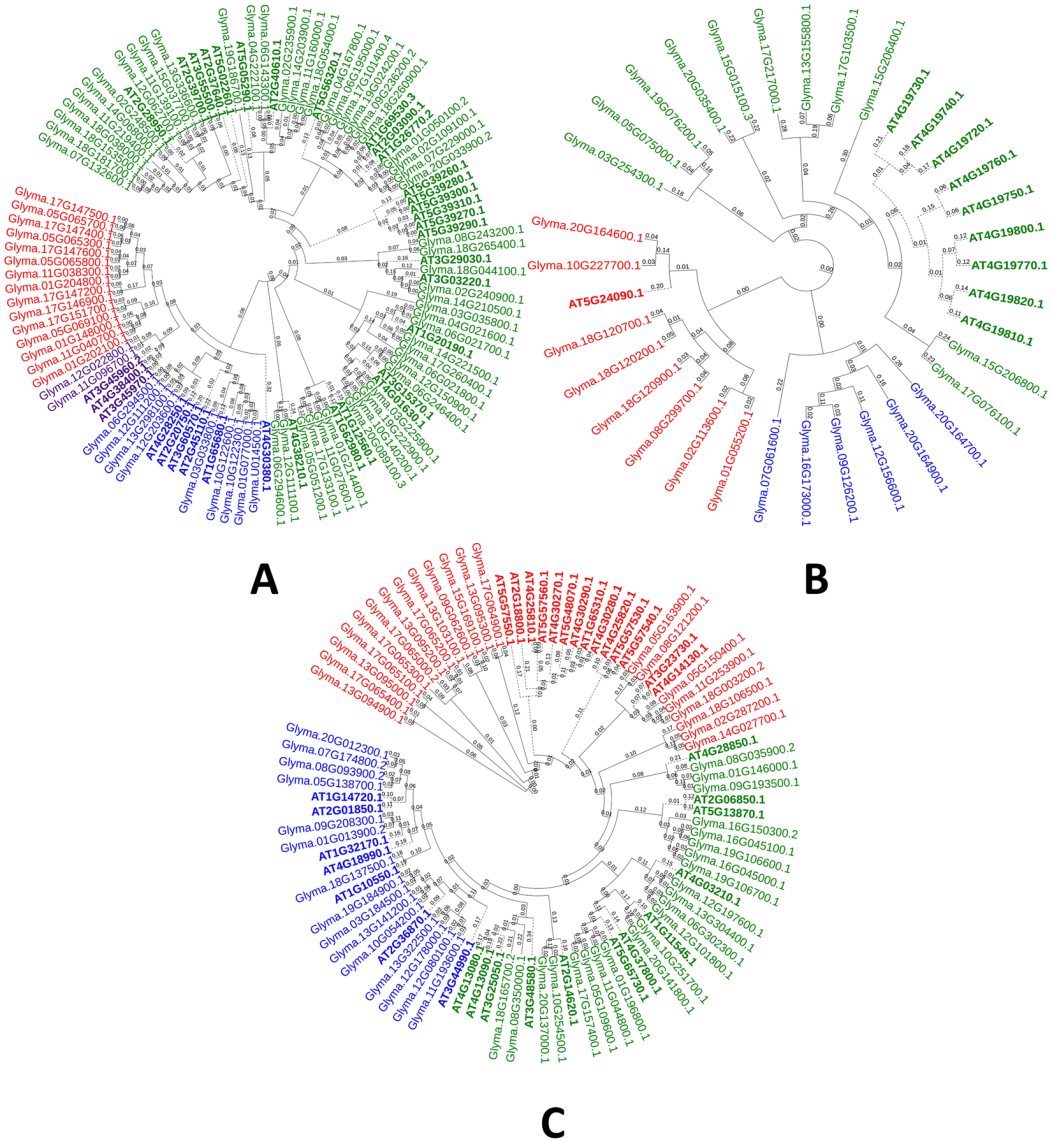
Phylogenetic tree of cell wall loosening related gene families in soybean. (**A**) Expansins, (**B**) yieldins and (**C**) Xyloglucan endotransglucosylases/hydrolases. Tree was constructed using Neighbour-Joining method with 500 times bootstrap values. *Arabidopsis* genes are represented with bold letters and dotted lines.

A total of 77, 25 and 58 genes were identified as the members of EXP, yieldins and XTH gene families, respectively. These gene families are principally involved in cell wall loosening (Table 1). The membership of the identified genes was primarily confirmed on the basis of presence of conserved domains i.e. PF03330 and PF01357 for EXPs, PF00704 for yieldins and PF00722 and PF06955 for XTHs. The phylogenetic tree with *Arabidopsis* EXPs is shown in Fig. 2A. The phylogeny showed four subfamilies i.e. EXPA, EXPB, EXLA and EXLB as previously studied^31^. Yieldins formed three major clades and the largest clade contained all *Arabidopsis* yieldins (except *At5G24090*.1) and grouped with 11 soybean yieldins. 8 soybean yieldins formed a clade with *At5G24090*.1, while remaining genes clustered into a third separate clade. Physical map revealed their presence on all chromosomes except Chr. 16. XTH family members were clustered in three main phylogenetic groups. All *Arabidopsis* XTHs and 54 soybean XTHs were grouped in one large phylogenetic group having 12 clades. XTHs showed distinct clustering with those of *Arabidopsis* (Fig. 2C). This classification was different from that of studied in monocots (rice and sorghum) where the family has been reported to be subdivided into three subfamilies^12, 32^. Maximum number of XTHs i.e. 7 was found on Chr. 6 (Fig. 1). Additionally, one scaffold gene i.e. *Glyma*.*U014500* was also found in this gene family. Phylogenetic tree of soybean yieldins with those of *Arabidopsis* showed no distinct clustering pattern (Fig. 2B). Yieldins were distributed on 16 soybean chromosomes except on Chr. 4, 6, 11 and 14. Maximum yieldins i.e. 4 were located on Chr. 20. XTHs were found on all chromosomes except Chr. 4 and the maximum number of 8 XTHs were located on Chr. 13 (Fig. 1).

### Glycoside hydrolases

**Figure 3:**
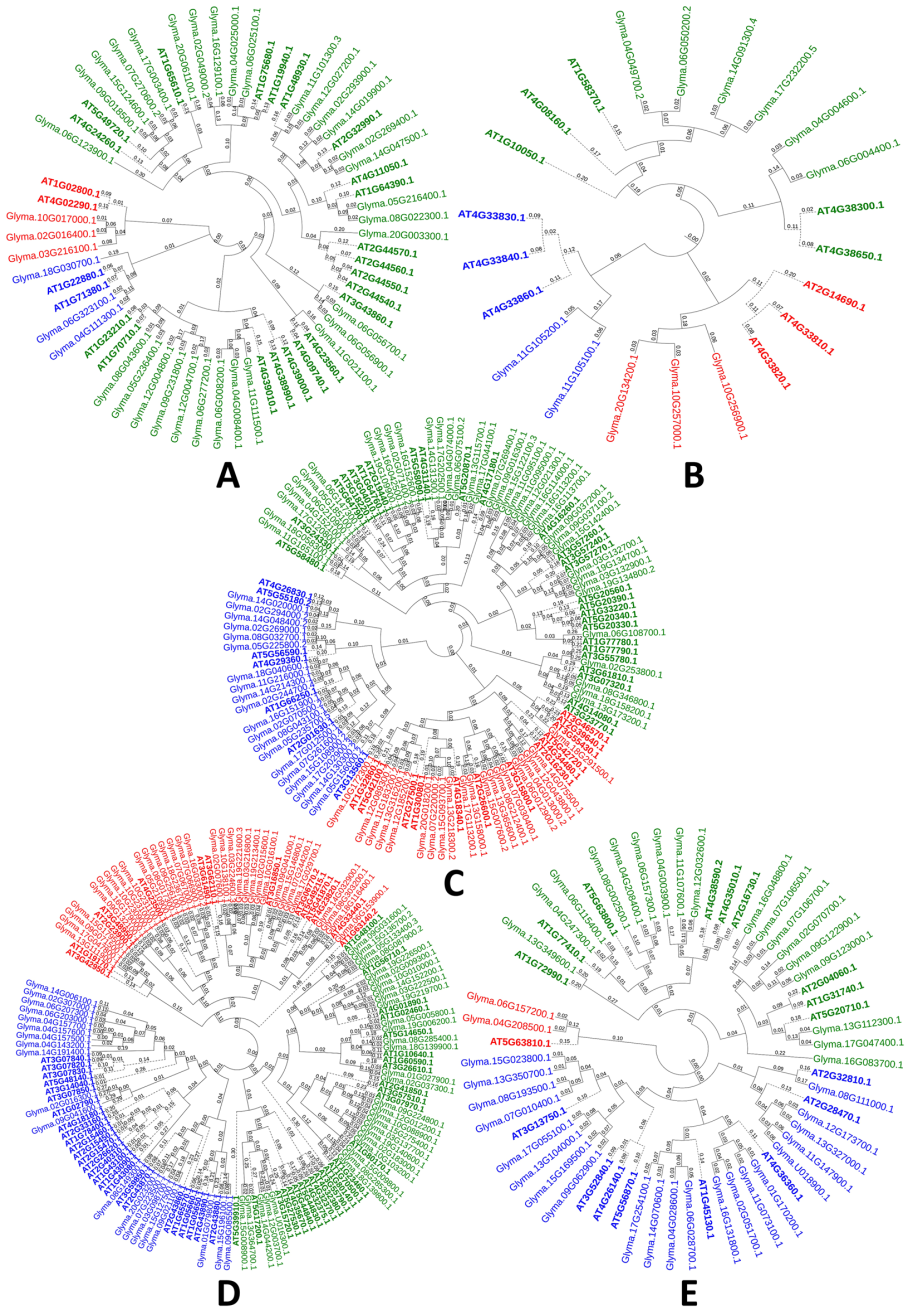
Phylogenetic tree representing five glycoside hydrolase gene families. (**A**) Endo-1, 4-β-glucanases, (**B**) Endo-xylanases, (**C**) Glucan 1, 3- β-glucosidases, (**D**) Polygalacturonases, and (**E**) β –Galactosidases. Tree was constructed using Neighbour-Joining method with 500 times bootstrap values. *Arabidopsis* genes are represented with bold letters and dotted lines.

GHs are important class of enzymes involved in the hydrolysis of glycosidic bond between two or more carbohydrates. The cocktail of GHs is used in bioethanol industry in hydrolysis of cell wall polymers after chemical pre-treatment^10^. In our study we focused on 7 soybean gene families i.e. GH9, GH10, GH16, GH17, GH8 and GH35. Two of GH gene families i.e. GH16 and GH18 are also involved in cell wall loosening process, therefore these families have been described with cell wall loosening gene families. With 84 genes GH28 was the largest gene family followed by GH17 with 78 genes, GH35 with 41 genes, GH9 with 37 genes and GH10 with 11 genes (Table 1; Supplementary Table S1). Based on conserved Pfam domains given in Table 1, the family membership was confirmed. Phylogenetic classification of GH9, GH10, GH17, GH28 and GH35 gene families showed the formation of distinct clade formation with those of *Arabidopsis* homologues (Fig. 3A–E). Soybean GH9s were divided into three subfamilies with largest subfamily containing 31 genes. GH10 gene family members grouped into three clusters. GH17 family of soybean was divided into three clusters and in each cluster there was unique clustering of soybean GH17s with *Arabidopsis* homologues. Among GH28s, three phylogenetic groups were formed which further subdivided into 4, 3 and 5 clusters, respectively. Soybean GH35 gene family members grouped into three main phylogenetic groups which subdivided into 1, 3 and 3 clades, respectively. Physical map of GH9 family showed the distribution of GH9 members 17 chromosomes except Chr. 1, 13 and 19, GH10 genes on Chr. 4, 6, 10, 11, 14, 17 and 20, GH17 genes on all chromosomes except Chr. 1, GH28 genes on all chromosomes except Chr. 11, and GH35 genes on 14 chromosomes except on Chr. 3, 5, 10, 18, 19 and 20.

### Pectin modifying gene families

**Figure 4:**
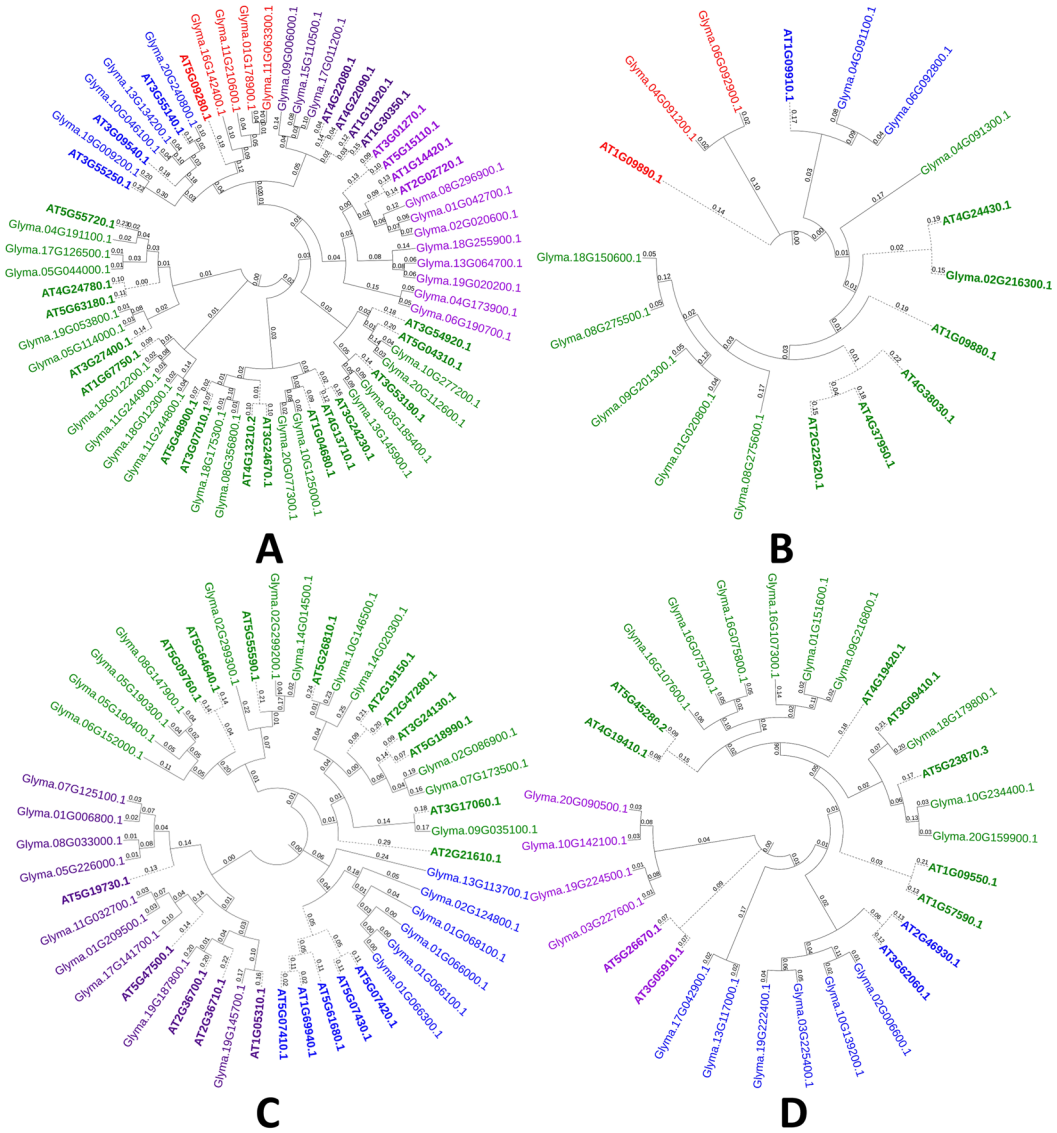
Phylogenetic tree representing pectin modifying gene families in soybean. (**A**) Pectate and pectin lyases, (**B**) Rhamnogalacturonana l lyases, (**C**) Pectin methyl esterases, and (**D**) Pectin acetyl esterases. Trees were constructed using Neighbour-Joining method with 500 times bootstrap values. *Arabidopsis* genes are represented with bold letters and dotted lines.

The first hurdle to hydrolysis is the selective cell-wall accessibility to GHs. This porosity feature is determined mainly by the pectin domain. To digest cell wall polymers for efficient ethanol generation, it is important to modify pectin through the action of pectin modifying enzymes^33^. A total of 94 gene members belonging to four pectin modifying gene families i.e. PL1, PL4, PME, and PAE were identified. Among pectin-related lyases (PLs) we found 37 and 11 genes for P1 and PL4 gene families, respectively. The membership of the identified genes to the respective families was confirmed by validating the presence of Pfam domain i.e. PF00544 and PF04431 for PL1 and PF06045, PF14686 and PF14683 for PL4 (Table 1). Phylogenetic analysis of PL1 grouped the genes into three groups with the largest containing 23 genes divided into five clades. PL4s showed their distribution into 3 main phylogenetic groups, third being the largest with 9 genes (Fig. 4A,B). Chromosomal distribution of members of PL1 gene family showed presence on all chromosomes except on Chr. 7, 12 and 14, while PL4 members were distributed on Chr. 1, 2, 4, 6, 8, 9 and 18.

The other two gene families i.e. PME and PAE, belonged to pectin esterases. We identified 19 PMEs and 27 PAEs in soybean based on their similarity with *Arabidopsis* pectin esterases and presence of conserved domains i.e. PF01095 and PF03283, respectively. The NJ trees showed that PAE and PME family members form distinct clades with *Arabidopsis* PMEs and were divided into three phylogenetic groups each (Fig. 4C,D).

### Gene structure analysis

Gene structure provides essential information related to gene function and conservation. Chromosomal locations, peptide sizes, protein molecular weights and isoelectric points for all gene families are given in Supplementary Table S1. To gain insight of evolution of CWRD gene families the intron/exon diversification was studied by knowing the exon/intron boundaries and intron phases. Among the cell wall loosening gene families maximum no. of introns was 5, 6 and 4 in EXP, yieldins and XTH, respectively. EXP subfamilies were characterized by similar intron/exon organization as reported elsewhere^31, 34^. However, among the whole EXP family, no conserved exons/intron was observed (Supplementary Table S2). Members of EXP gene family were found in phase 1; 2; 1, 2; and 0, 1, 2 implying that either during the course of evolution some member genes have experienced either gain or loss of introns, resulting into evolution of subfamilies. Interestingly, among analyzed gene families only yieldins showed intronless genes (8 genes). Because the ratio of the intronless gene to genes with introns was 8: 17 so it is possibly due to intron-loss. Furthermore, only three soybean yieldins had introns in phase 0, 1, 2 while all other were in different phases i.e. 0, 1 or 2. Average peptide length of EXPs, yieldins and XTHs was 256, 372 and 297 amino acids (Supplementary Table S2). Among GHs, exons length conservation was observed within subfamilies/phylogenetic groups of each gene family. GH35 family members showed a higher number of conserved exons within the family and subfamilies/phylogenetic groups. This might be because of longer gene sizes with a higher number of introns and exons. Maximum no. of introns in GH9, GH10, GH17, GH28 and GH35 (with 1 intronless gene) were 10, 7, 13, 11 and 20, respectively. Most of GH9 family member introns were either in phase 0 or 0, 1 except two genes having introns in 0, 1, 2 phases. Intron phase was not conserved in GH10 and GH17 gene families. On the other hand, GH28 gene family members tended to have an intron in phase 0, 1, 2. Strong conservation of intron phase was observed in GH35 gene family i.e. all introns were in phase 0, 1, 2. Average peptide sizes were 532, 650, 436, 451 and 802 residues for GH9, GH10, GH17, GH28 and GH35 gene families. Pectin modifying genes showed variation in maximum intron number, PL1, PL4, PAE and PME having 6, 16, 16 and 5, respectively. Intron phase was highly conserved (all genes having introns with phase 0, 1, 2) in PAE gene family members while the members of PME gene family also showed intron phase conservation with some variations. Average peptide size of PL1, PL4, PAE and PME members was 412, 642, 422 and 370 amino acids.

### Duplication and Synteny analysis

**Figure 5:**
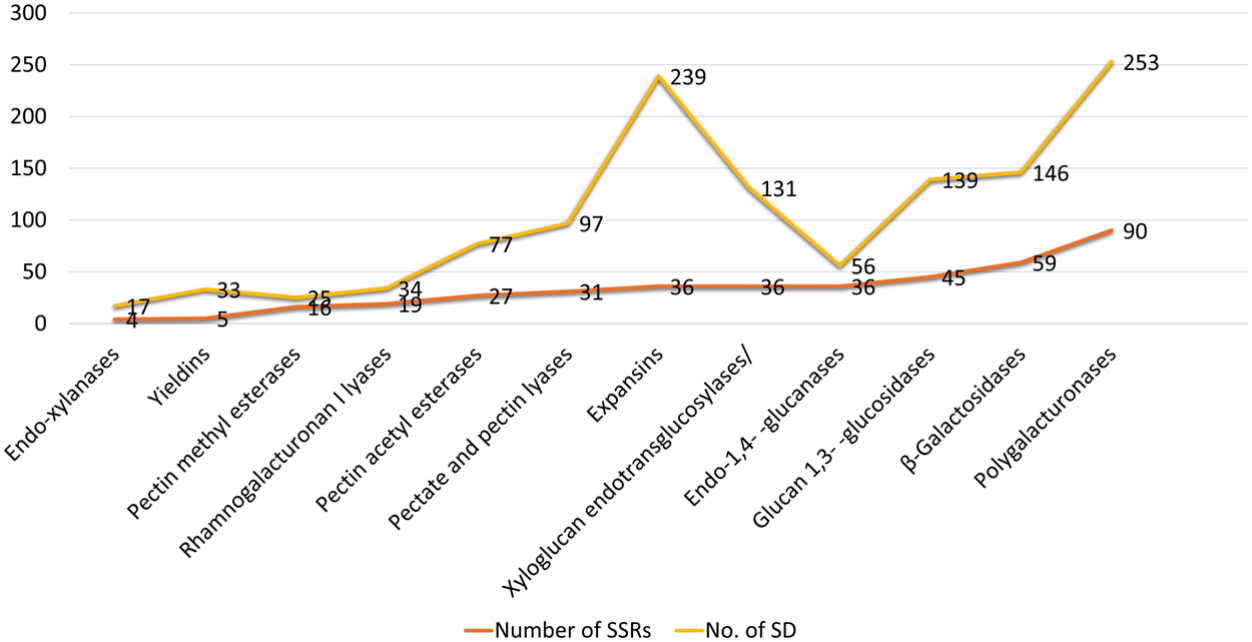
Details of identified SSR markers and Tandem duplications in 505 cell wall reassembly and degradation related genes of soybean. Gene family wise distribution of identified SSR markers and Tandem duplications.

We analyzed all CWRD related gene families for the presence of tandem duplication (TD) in order to reveal any expansion during evolution. Interestingly, out of 505 identified genes, we witnessed 1262 TDs in 401 genes (~80%). Maximum number of TDs (253) were observed in GH28 gene family and minimum number of TDs (17) were found in GH10 gene family (Fig. 5). Among cell wall loosening gene families, EXPs (254 events/55 genes), yieldins (33 events/16 genes) and XTHs (131 evens/45 genes) were observed. In GHs a total of 611 events were observed in 208 genes while in pectin modifying gene families 70 genes experienced 233 TD events (Supplementary Table S3). Chromosome 2 experienced maximum number of duplications i.e. 86 and Chr. 20 experienced minimum duplication events i.e. 32.

The estimated time of duplication for all CWRD related gene families ranged from 0 to ~300 mya. The most old duplication events took place in members of GH28 i.e. 300.82 mya followed by PL4 i.e. 296.72 mya. Among the cell wall loosening gene family members, EXPs duplicated around 61.47 to 177.05 mya, yieldins duplicated around 7.37 to 222.95 mya and XTHs duplicated around 4.92 to 282.78 mya. While those of GHs duplicated around 0 to ~300 mya. The pectin modifying gene family PL1 duplicated around 0–280.32 mya, PL4 duplicated around 0–296.72 mya, PAE duplicated around 0–165.57 mya and PME duplicated around 3.27 to 177.86 mya. Synteny within each soybean CWRD gene family members is given in Supplementary Fig. S1. The comparative synteny relationship map revealed a high degree of similarity within soybean CWRD related gene family members.

### Simple Sequence Repeat Markers in CWRD related genes in soybean

Microsatellites are efficient molecular markers which are distributed across the eukaryotic genomes^2, 15, 35^. These are markers of choice mainly because of their genome-wide distribution and ability to detect polymorphism. They are considered an efficient marker system due to taxon-specific variation in motif structure, the frequency of occurrence, and genomic location and are an important tool for marker assisted selection. Such a tool can aid selection of parents for developing soybean lines and mutants for biofuel applications^12, 15^. Keeping in view their utility in soybean improvement stratagems, simple to score length variations and ease of mining, we analyzed soybean CWRD related gene family members for the presence of SSRs. Out of 505 genes, 316 had 687 SSR markers (Table 2; Supplementary Table S4). Mining for SSR markers resulted in the identification of 189 dinucleotides, 165 trinucleotides, 230 tetranucleotide, 74 pentanucleotides, and 29 hexanucleotide repeats (Table 2). Dinucleotide repeats were the most abundant SSRs in soybean CWRD. We found SSR markers in all 12 studies gene families. Maximum number of SSRs (166) was found in GH28 gene family.

**Table 2:** Summary of putative SSRs identified in soybean CWDE.

Parameter	Details
Total number of sequences examined	505
Total size of examined sequences (bp)	1981887
Total number of identified SSRs	687
Number of SSR containing sequences	316
Number of sequences containing more than 1 SSR	316
Distribution of different repeat type classes	
Dinucleotide repeats	189
Trinucleotide repeats	165
Tetranucleotide repeats	230
Pentanucleotide repeats	74
Hexanucleotide repeats	29
Number of Sequences with successful primer pairs	319
Number of sequences without primer pair picked	31
Total primer pairs picked	695

### Soybean CWRD genes with putative miRNA target sites

MicroRNAs (~21-nt long) regulate gene expression at transcriptional and post-transcriptional levels. Post-transcriptional regulation can contribute considerably to phenotypic modifications during plant development^36^. Here we explored the putative miRNA target sites in soybean CWRD gene family members. A total of 10 genes belonging to EXP (2), GH9 (4), GH10 (1), and GH17 (3) contained putative miRNA target sites. The BLAST alignment algorithm identified 13 query sequences with high statistical confidence and sequence homology to the short sequences present in the degradome database. On the other hand, 18 predicted miRNA targets didn’t retrieve any significant hits. Overall, our *in silico* analysis followed by a further validation in an independent dataset identified potential novel miRNA targets in the studied CWRD gene families. (Supplementary Table S5).

### Expression potential of soybean CWRD genes

#### Digital expression in soybean plant development and anatomical parts

**Figure 6:**
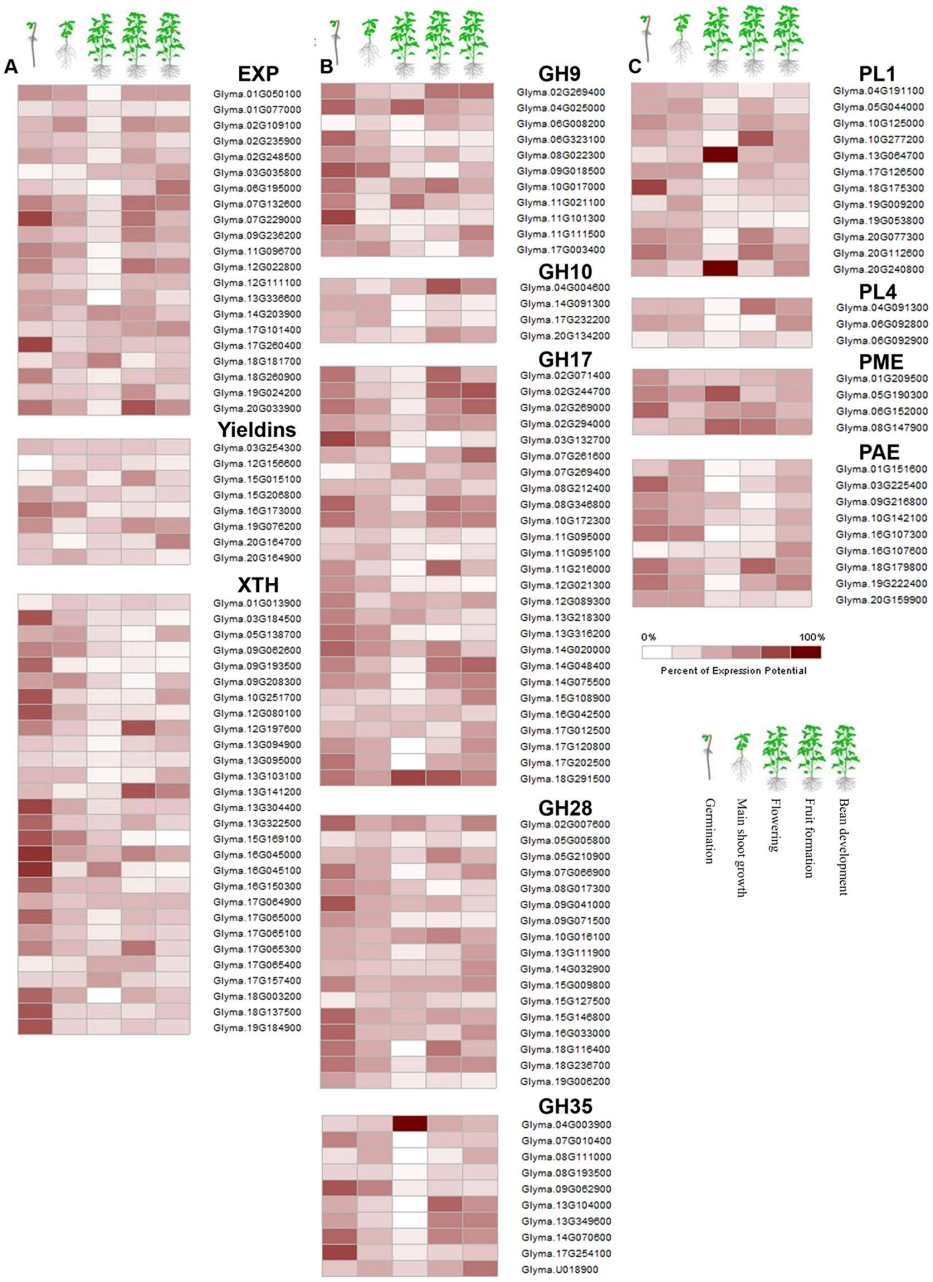
Expression potential of soybean CWRD genes in soybean plant development. (**A**) Digital expression potential of cell wall loosening genes, (**B**) digital expression potential of glycoside hydrolases, and (**C**) digital expression potential of pectin modifying genes in five soybean plant developmental stages i.e. germination, main shoot growth, flowering, fruit formation and bean development.

Recent developments in bioinformatics has facilitated availability of whole genome, proteome, metabolome and microarray data for major crops. To determine expression potential of CWRD genes in soybean development and 68 anatomical parts we used GENEVESTIGATOR and accessed the spatial expression profiles. The expression potential in five soybean plant developmental stages and different anatomical parts is presented as heat maps for individual gene families to better visualize and understand (Fig. 6; Supplementary Fig. S2). Among cell wall loosening gene families, EXP gene members showed differential expression potential with notable expression of *Glyma*.*07G229000* and *Glyma*.*17G260400* in germination stage. Interestingly one gene i.e. *Glyma*.*20G033900* showed higher expression during bean development stage. All EXPs had notable expression during cell culture and primary cell development. *Glyma*.*20G033900* was highly expressed throughout the anatomical parts of soybean plant except flowering stage. Among yieldins, all genes showed varied and relatively lower expression throughout five developmental stages. Similar expression profile was observed in all 68 anatomical parts. All XTHs showed higher expression potential in germination stage and lower or varied expression in remaining four developmental stages (Fig. 6A). Interestingly, same expression pattern was observed in anatomical parts of seedlings i.e. shoot apix (shoot apical meristem), hypocotyl and radicle (maturation zone and root hair). *Glyma*.*12G197600* and *Glyma*.*13G141200* had 100% expression potential in cotyledon, epidermis (abaxial and adaxial), hilum and endothelium (Supplementary Fig. S2a). The GH gene families GH9, GH17, GH28 and GH35 showed varied expression potential throughout the plant developmental stages. GH9 family members showed differential expression in all 68 anatomical parts with maximum expression potential in seedling (radicle; maturation zone and root hair). GH10 genes showed very low expression throughout the plant anatomical parts except the different compartments of seed where the expression was maximum. GH17s showed varied but relatively higher expression throughout plant anatomical parts except in inflorescence and flower compartments and leaf. GH28s had varied expression potential in all anatomical parts of soybean plant. Among GH35s, *Glyma*.*04G003900* showed 100% expression potential in inflorescence (raceme, flower, androecium, stamen, anther and pollen). *Glyma*.*13G10400*, *Glyma*.*13G349600*, *Glyma*.*14G070600*, *Glyma*.*17G254100* and *Glyma*.*U018900* had higher expression in seed (testa, outer integument, palisade layer, hourglass-cell layer, inner-integument and parenchyma) (Supplementary Fig. S2b).

Pectin modifying enzymes showed consistent but lower expression throughout five soybean plant development stages. Two PL1 genes i.e. *Glyma*.*13G64700* and *Glyma*.*20G240800* showed 100% expression potential in shoot development stage (Fig. 6C). Among PL1s, two genes i.e. *Glyma*.*05G04400* and *Glyma*.*19G09200* had 100% expression potential in cell culture and primary cell development (leaf cell, mesophyll cell; paraveinal mesophyll cell and palisade parenchyma cell). Interestingly, two PL1s (*Glyma*.*13G064700* and *Glyma*.*20G240800*) showed maximum expression in inflorescence related anatomical parts. All pectin modifying enzyme related genes showed maximum expression during hourglass-cell layer of seed testa (Supplementary Fig. S2c).

#### Digital expression under abiotic stress

Plants exposed to abiotic stresses display morphological changes that in turn modify cell wall such as decrease in thickness and increased level of cell wall bound phenolics^37–39^. We determined expression potential of CWRD related gene family members under heat stress and salt stress. It was notable that expression of EXPs was same in plants with 20% and 30% photosynthesis inhibition (at 42 °C) and 22 °C as compared to control. However, four EXPs (*Glyma*.*01G05100*, *Glyma*.*02G109100*, *Glyma*.*19G024200* and *Glyma*.*20G033900*) was relatively higher than other members of the same gene family (Supplementary Fig. S3a). Yieldins also displayed no difference in expression potential under heat stress and control conditions. Two XTH members i.e. *Glyma*.*09G062600* and *Glyma*.*15G169100* showed increased expression potential under heath stress (42 °C) in comparison to controls. Among the GH families, GH9 (except *Glyma*.*4G025000*, and *Glyma*.*9G018500*), GH17 (except *Glyma*.*11G095000*, *Glyma*.*11G095100*, *Glyma*.*12G021300*, and *Glyma*.*14G020000*), GH28 (except *Glyma*.*15G127500* and *Glyma*.*18G116400*) and GH35 (except *Glyma*.*08G1000*, *Glyma*.*09G062900* and *Glyma*.*U018900*) had similar expression pattern under heat stress and control (Supplementary Fig. S3a). Among pectin modifying gene families, PL4 and PME gene members showed no variations in expression potential however, members of PAE and PL1 showed variations in expression under heat stress and control. Notable expression potential was observed in *Glyma*.*19G009200* (PL1) and *Glyma*.*10G142100* and *Glyma*.*16G107300* (PAE) (Supplementary Fig. S3a).

In Alkaline stress of 50 Mm NaHCO_3_ for 3, 6 and 12 hours, EXP gene family members showed an increase in expression potential with respect to time of exposure. A similar pattern was observed in yieldins and XTHs (Supplementary Fig. S3b). Among GHs, GH9s especially *Glyma*.*02G269400* and *Glyma*.*04G025000* showed higher expression potential when exposed to 3 and 6 hours alkaline stress as compared to 12 hours exposure time. GH10s showed very low but increasing expression pattern in response to increasing exposure time. GH17 gene family members expressed at higher levels when the exposure time was increased to 12 hours. GH28 and GH35 family member genes showed expression potential similar to those of XTHs (Supplementary Fig. S3b). Among pectin modifying enzymes, PL1 and PL2 and PME showed increasing expression potential in response to increasing exposure time while the members of PAE expressed at very higher levels during 3 and 6 hours exposure time as compared to 12 hours (Supplementary Fig. S3b).

#### Digital expression under biotic stress

The dynamic structure of cell wall determines the outcome of the plant-pathogen interactions^40^. The microbial pathogens exploit cell wall reassembly and degradation machinery of the host to facilitate pathogenesis. In order to understand the response of CWRD related gene family members again pathogen attack, we used Affymetrix data^41^ and https://www.ncbi.nlm.nih.gov/geo/query/acc.cgi?acc = GSE7124) during *Bradyrhizobium* *japonicum* and *Phytophthora* *sojae* infestations, respectively. All CWRD related gene family members showed increased expression potential as compared to control (Supplementary Fig. S3c) in *B*. *japonicum* infected plants. Interestingly, EXPs, XTHs, and GH9s showed maximum expression potential (up to 100%). Among GH gene families, lowest expression potentials were observed in gene members of GH10 while among pectin modifying gene families, PL4 had the lowest expression potential. Interestingly, the expression pattern of genes of CWRD families was higher in early infection period i.e. 6 and 12 hours and either decreased or remained unchanged with increased infection period (18 to 24 hours) (Supplementary Fig. S3c). The expression pattern of CWRD genes under the infection of *P*. *sojae* is given in Supplementary Fig. S3d. Among the cell wall loosening gene families, the expression potential was similar. It was notable that expression potential increased when the infection period was increased from 72 to 120 hours. All gene family members of GHs showed higher expression under prolonged infection time as compared to control. Among pectin modifying enzymes, the expression of PL2 and PME gene family members was lowest but comparable among treatments. PL1 and PAE gene families members were highly expressed under prolonged infection period as compared to lower infection time and control (Supplementary Fig. S3d).

### Co-expression networks of soybean CWRD gene families

Availability of postgenomic technologies supported with genome sequences and expression data has made it easier to understand how biological systems work by analyzing co-expression networks of functionally related genes which are transcriptionally coordinated^22^. To broaden our understanding of transcriptional coordination of soybean CWRD related gene families, we developed co-expression networks of individual gene families by using PlaNet platform which uses available Affymetrix GeneChip microarray datasets (Supplementary Fig. S4 and Supplementary Table S6).

Co-expression network of EXP gene family showed 54 nodes involved in 36 biological processes, two cellular compartments, and 11 molecular functions. Most importantly, it contained nodes related to biological processes in response to different biotic and abiotic stimuli, regulation of cellular process, plant cell wall organization, and cell wall polysaccharide metabolic process etc. (Supplementary Table S6b). Among CWRD related gene families, PME and GH28 gene members had co-expression with EXPs (Supplementary Fig. S4a). Thirty-four nodes co-expressed with yieldins (query gene *glyma03g41510*). The co-expressed genes were involved in 14 biological processes and 14 molecular functions. All the co-expressed nodes belonged to different gene families involved in multiple biological processes but none was the member of CWRD related gene family (Supplementary Fig. S4b). Fifty nodes co-expressed with XTH gene family member *glyma09g34140* which were involved in 13 biological processes with notably in cellular glucan metabolic process. The co-expressed genes were found to be involved in 10 molecular functions in five cellular compartments (Supplementary Fig. S4c, Supplementary Table S6a,b).

Among GHs, GH9 (query gene *glyma04g02740; glyma06g02760*) was co-expressed with 163 nodes involved in 21 different biological processes having 44 different molecular functions within 27 cellular compartments (Supplementary Fig. S4d, Supplementary Table S6a). Among molecular functions, microtubule motor activity, structural constituent of the cytoskeleton, cellulose synthase activity and lyase activity were notable (Supplementary Table S6b). PL1, GH17, GH28, GH35, XTHs, PAE, PME, cellulose synthase and COBRA gene families were observed in co-expression network of GH9 gene family (Supplementary Table S6a). GH10 showed co-expression with members of yieldins gene family (Supplementary Fig. S4e). Co-expression network of GH17 family member showed that GH28 had some functional correlation (Supplementary Fig. S4f). Similarly, GH28 and GH35 gene family members showed co-expression with PL1, GH35, PAE, and PAE (Supplementary Fig. S4g and Fig. 3H).

Among pectin modifying enzymes, co-expression networks revealed that GH9, PL1, PAE, and GH28 (Supplementary Fig. S4i–k). The biological processes, cellular components and molecular functions of co-expressed genes are given in Supplementary Table S6b. The annotations and labels of co-expressed genes are given in Supplementary Table S6a. Notably, Tubulin Tubulin_C and cellulose synthases were found in co-expression network of most of CWRD related gene families (Supplementary Table S6a).

## Discussion

Among the key processes involved in second generation biofuel production, cell wall hydrolysis is an essential step, which demands identification and characterization of enzymes dealing with the cell wall loosening, hydrolysis, and degradation. The possible two ways to find possible candidates related to CWRD enzymes are either identify genes and proteins in different microorganisms which they use to break plant cell walls during pathogen attack or by controlling *in planta* mechanisms involved in CWRD^12^. It has been suggested previously that controlling early and over production of GHs and pectin modifying enzymes within plants will greatly aid the step-wise cell wall degradation process^10^. Successful production of biofuels from plant cell wall polymers calls for a comprehensive understanding of the genes and their transcriptional coordination. Soybean has been proposed as a resourceful biofuel crop^6^, however, a comprehensive report on CWRD related genes was missing in soybean. Identification of genes and their distribution will greatly comprehend our understanding about the natural processes involved in CWRD in soybean. Such a knowledge will help to improve the efficiency of degradation of cell walls and reduction of recalcitrance by modifying biomass composition. On the long run, such an inventory of genes would augment the plant breeders and biotechnologists to design and engineer plants having the ability to change or modify their cell walls in order to produce endogenous biological pre-treatments. In this study, 505 genes belonging to 12 CWRD related gene families have been identified and characterized *in silico*.

The identified genes were distributed in to 3 groups i.e. cell wall loosening genes families, GHs and pectin modifying gene families (Table 1). Conserved domain analysis and phylogeny constructed with *Arabidopsis* CWRD proteins suggested that these proteins are conserved (Figs 1, 2 and 3) and greatly supported by other studies^11, 34^. Gene structure analysis supported with intron or exon boundaries and intron phases greatly help to identify conserved exons and clarify the conservation or divergence of gene structure and functions within gene families^42^. High conservation of intron phases in GH35 and PAE gene families suggests the possibility of shuffling of symmetrical exons between the same phase-introns. The correlation of intron-exon boundaries with three dimensional structure of proteins has also been proposed^43^. Relatively higher number of CWRD related genes in soybean is most probably due to its late linage-specific duplication. Two rounds of whole genome duplications, shuffling of symmetric exons among introns having same phases further clarifies the expansion of CWRD gene families of soybean^34, 44^. Incredibly larger number of TD i.e. 1262 duplication events (~80%), greatly contributed towards expansion and diversification and implies that majority of studied genes are duplicated. This further suggests the expansion of these gene families has been mainly achieved by the retention of gene copies after two rounds of whole genome duplications. This expansion feature has been previously reported in EXP gene family of soybean, *Arachis duranensis* and *Arachis ipaensis* ^31, 34^

Such gene retentions and diversifications of genes copies in soybean CWRD gene families further suggests sub-functionalization of these gene families, which may result in different cell wall compositions. This evolutionary process may also support neofunctionalization as already established in cell wall compositional differences in eudicots and grasses^45^. Higher numbers of CWRD genes in soybean as compared to *Arabidopsis* advocates a selective advantage to preserve these multiple copies after a series of duplication. Abundance of CWRD genes were distributed on all 20 soybean chromosomes forming small clusters of same gene family members. Such clusters could be considered as hotspot targets for breeders and molecular biologists to engineer soybean for *in planta* loosening of cell walls which is strongly needed during pre-treatment of molasses for bioethanol production^6, 10, 11^.

Co-dominance, genome-wide distribution, ease of handling and detection and multi-allelic nature makes SSR markers a good candidate to support marker assisted breeding, map-based cloning and detection of specific genes^15, 46^. Engineering and breeding plants for higher biomass production and or *in planta* cell wall loosening could be accelerated by identifying molecular markers linked with the CWRD related genes. Such a strategy has been successfully used in alfalfa where marker-assisted selected was carried out to identify QTLs for enhanced biomass yield and cell wall compositional variations (Li *et al*. 2011). Identification of soybean CWRD related SSRs with greatly comprehends the selection of suitable cultivars or breeding lines for enhanced biomass production for the biofuel industry. Furthermore, these makers would help to screen and identify mutants^46^. *In silico* SSR identification along with a genome-wide analysis of CWRD related genes has been successfully adopted elsewhere^12, 47^. The identified SSR markers in our study showed a higher proportion of tetranucleotide repeats which is in line with our previous report related to cellulose synthase gene superfamily in soybean^15^ as well as reported in coffee^48^ (Table 2). Identification of cell wall-related genes based on molecular markers has also been successfully done in *Arabidopsis*, rice^49, 50^. Additional to SSRs, our study identified the presence of putative miRNA target sites (Supplementary Table S5). These miRNAs are one of the major types of endogenous non-coding RNAs involved in post-transcriptional/translational modifications in gene expression. The role of miRNAs during cell wall biogenesis has been confirmed in plants^51^. Shen *et al*.^52^ characterized miRNAs in response to the pathogenic attack of *Verticillium longisporum* in alfalfa. The expression is obviously related to cell defense structure i.e. cell wall. The identified families of miRNAs in our studies has been previously reported to be involved in different plant defence mechanism related to cell wall response to abiotic and biotic stress responses in soybean and other plants^53–56^. These studies greatly support that the expression these miRNAs greatly effect plant cell wall composition under different stimuli. Such a knowledge of miRNA target sites in uncharacterized CWRD related genes will increase our understanding of post-transcriptional/translational modifications.

Cell wall compositional variations accompanied by varied gene expression levels has been reported in different plant tissues^29, 57^. Expression potential analysis of CWRD related genes provided essential knowledge to determine spatiotemporal role of these genes and will help to decide at which plant developmental stage it could yield higher biomass. A similar approach was reported in sorghum^11^. We determined expression potential of soybean CWRD related genes at five plant developmental stages and 68 anatomical parts (Fig. 6 and Supplementary Fig. S1). Which in turn highlighted many tissue/anatomical part specific genes involved in CWRD, these could be potential candidates to engineer these tissues/anatomical parts for achieving higher biomass yields. A recent study on expression patterns of pectin, cellulose, cell, wall, lignin and fatty acid related genes was done in different soybean tissues^5^. In our studies, varied expression potential of all members of 12 studies gene families was observed in five soybean plant developmental stages. Relatively higher expression potential was observed at seedling stage for all gene families suggesting extensive cell wall modification during soybean germination stage occur (Fig. 6). During later four plant growth stages, all CWRD related genes were expressed. A more detailed expression potential analysis of studied gene families of soybean revealed that among the studied gene family groups GHs had relatively higher expression potential (Supplementary Fig. S1b). This could shed light upon spatiotemporal expression of GHs relative to other plant CWRD related gene families, especially those involved in cell wall loosening. Plant cell wall growth is resulted simultaneously by synthesis of cell wall polysaccharides as well as the expansion of existing polysaccharides^29^ as observed by Ohmiya *et al*.^58^ while studying members of GH9. Among cell wall loosening gene families higher expression potential of EXPs and XTHs in cell culture, shoot apical meristem, radicle and maturation zone during germination stages and endosperm, testa and integuments at pod elongation stages suggests their roles in the shoot and pod elongation (Supplementary Fig. S1a). A similar correlation of expression of a soybean EXP gene i.e. *GmEXP1* with root elongation was observed^59^. A detailed review on mechanism and action of different enzymes was recently published by Cosgrove^60^ who explained about the cell wall role of EXPs. Higher expression of PMEs and PAEs in growing anatomical parts such as seedling (radicle, root hairs, and meristems), cotyledon, testa (hourglass-cell layer and parenchyma cells) and primary roots were in line with previous studies^61, 62^ (Supplementary Fig. S1c).

It is evident from the genetic studies that analogous gene family members (e.g., XTH, EXPs, and PLs) are frequently involved in the response to different stresses^63^. However, the intrinsic complexity of the cell wall and a large number of genes involved in its biosynthesis, assembly, loosening, reassembly, and degradation means that many details remain unclear regarding the genetic and biochemical basis for cell wall responses to stress^64^ Meides *et al*. 2014). Plants respond to abiotic stresses by various morphological, physiological and biochemical mechanisms^65–67^. The improvement of genomics and proteomics technologies with the availability of massive publically available data. Many studies have compared cell wall modification under abiotic stress with controls^5, 64, 68^. Higher expression potential of EXPs and XTHs under heat stress in our studies is strongly in line with the previously established fact that XTHs act as tethering polymers between cellulose fibrils^64, 68–70^. EXPs and XTHs showing higher expression potential could be strong candidates for engineering soybean for abiotic stress tolerance as well as for *in planta* cell wall loosening (Supplementary Fig. S2a). A soybean EXP gene i.e. GmEXPB2 was characterized and against abiotic stresses and was involved in root system architecture^39^. Similarly, those GHs which showed higher expression potential can be selected for breeding soybean with higher abiotic stress tolerance because GHs cover a broader range of molecular functions and also interact with plants hormones for their activation and inactivation^29, 68^. PAEs showed higher expression potentials in heat stress conditions as compared to controls (Supplementary Fig. S2a). Statistically significant variations in expression of PAEs, PMEs and PLs in barley caryopses exposed to heat stress have been previously reported^71, 72^. The change in expression potential of CWRD genes in response to salt stress is in line with previous reports in *Arabidopsis*, tobacco, and Jatropha^73–76^. Under salt stress conditions, the upregulation of XTHs and GHs was witnessed in *Arabidopsis* ^77^. Thus, many abiotic stress conditions lead to an altered response of CWRD related genes^64^.

Remodeling of cell wall under continuous threat of pathogen attack is a key response of plants^78^. The dynamic structure of cell wall determines the outcome of the plant-pathogen interactions^40^. The increased expression potential of soybean CWRD genes was observed in our *in silico* digital expression analysis by using available Affymetrix microarray data (Supplementary Fig. S2c,d). Previous transcriptome profiling studies revealed that 182 cell wall-related genes were differentially regulated in response to *B*. *japonicum* infection in soybean^79^. Decreased susceptibility to different pathogenic infections was observed by overexpressing PMEs in *Arabidopsis*, wheat, tomato, Populus, strawberry, broccoli and pepper^80^. Indeed most of CWRD genes especially GHs play significant roles in plant defense mechanisms and cell wall modification in response to pathogen attack^29^. The notable higher expression potential of XTHs in response to *B*. *japonicum* and *P*. *sojae* infection presented many potential candidates for engineering cell wall for biotic stresses as well as for bioethanol production.

Recent developments in bioinformatics and availability of genomics and proteomics data have enabled to explore more about the networks of genes regulating plant cell wall modification, loosening, degradation and hydrolysis^63^. Previously, co-expression networks of cell wall synthesis related genes have been explored in *Arabidopsis*, rice, and soybean^15, 22, 81^. We explored co-expressed networks of soybean CWRD genes to facilitate identification of genes for larger biomass production and *in planta* cell wall loosening (Supplementary Fig. S3a–k). This, in turn, shed light on correlated molecular functions in different biological processes related to cell wall synthesis, loosening, degradation. Such studies will aid *in vitro* and *in vivo* studies target to identify co-expressed genes under biotic and abiotic stresses as recently done in *Arabidopsis* ^82^. The transcriptional coordination of EXPs with PMEs and member of GH28 gene family is of great interest and could be the foundation of future studies targeted to investigate their coordination. The co-expression of cellulose synthases with all gene families is previously explored in many studies as discussed by Houston *et al*.^63^ and Miedes *et al*.^78^. The combined role of PLs with EXPs and GHs has already been under discussion and many reports showed the interaction of XTHs, EXPs with pectin modifying gene family members^63^.

## Methods

### Data retrieval and identification of CWRD related gene families

For the identification and analysis of soybean CWRD related gene families, publically available genomic, cds and peptide sequences were downloaded from Phytozome 11 database^83^ (https://phytozome.jgi.doe.gov/pz/portal.html) by conducting a BLASTp 2.2.28+ search. BLASTp was performed by using *Arabidopsis* CWRD related gene family members as queries to retrieve soybean CWRD related gene family members with an *E*-value of 10^−5^. All identified putative CWRD related proteins were further confirmed the presence of family specific conserved domains using NCBI’s Conserved Domain Database (CDD); (http://www.ncbi.nlm.nih.gov/Structure/cdd/wrpsb.cgi) and EMBL InterProScan^84^ (https://www.ebi.ac.uk/interpro/). After confirming the CDDs for all CWRD related putative proteins, we further obtain gene IDs, functional annotation, chromosome locations, chromosome number, genomic coordinates and peptide sizes from Phytozome database and used for further analysis. Protein molecular weight and pI-values for each identified proteins were calculated using ExPASy online tool Compute pI/MW^85^ (http://web.expasy.org/compute_pi/).

### Phylogeny, gene structure analysis, and physical mapping

Protein sequences of individual *Arabidopsis* and soybean CWRD gene families were aligned in ClustalW program of MEGA 7.0^86^ to construct respective phylogenetic trees using neighbour-joining (NJ) method with a bootstrap value of 500 iterations. All positions containing gaps and missing data were excluded in order to achieve phylogenetic trees. Finally, the trees were visualized and managed in iTOL^87^ (http://itol.embl.de/). To generate a physical map of CWRD related gene member on soybean chromosomes, we used chromosome numbers and genomic coordinates in Mapchart 2.30^88^. An intron-exon map of all individual gene families was generated in accordance with previous reports. Gene Structure Display Server^89^ (GSDS; (http://gsds.cbi.pku.edu.cn/) was used to determine intron phase and number of introns and exons. The exon-intron boundaries were determined by employing Analyze feature of PhytoMine (https://phytozome.jgi.doe.gov/phytomine/).

### Duplication and Synteny analysis

All identified members of CWRD related gene families were subjected to duplication analysis within the genome using Plant Genome Duplication Database^59^ (PGDD); http://chibba.agtec.uga.edu/). To avoid saturation anchors of >1.0 identity were rejected within a 100-kb range. Approximate dates of duplication events were calculated according to the method described by Lynch and Conery^90^. Briefly Ka (ratio of nonsynonymous substitutions per nonsynonymous sites) and Ks (synonymous substitutions per synonymous site) were retrieved from PGDD and were then used to calculate estimated duplication date in million years ago (mya) based on a rate of 6.1 × 10 − 9 substitutions per site per year, we calculated the divergence time (*T*) as *T* = Ks/(2 × 6.1 × 10 − 9) × 10 − 6 Mya^87^. Next, to identify syntenic regions within each soybean CWRD related gene family, we used Circoletto^91^ (http://tools.bat.infspire.org/circoletto/).

### *In silico* SSR Markers and miRNA target sites prediction

Genomic sequences of 505 soybean CWRD related gene members were subjected to identification of SSRs in MIcroSAtellite identification tool (MISA(, http://pgrc.ipk-gatersleben.de/misa/misa.html)). We included only those repeats which had stretches of a minimum of five repeats. We searched for repeat units for di, tri, tetra, penta and hexa nucleotides and the maximum distance between two markers was set to 100. To confirm the number and exact motifs for each SSR in all CWRD related genes and design primers, we further used BatchPrimer3 v1.0 (http://batchprimer3.bioinformatics.ucdavis.edu/). To detect the presence of putative miRNA target sites, the CDS sequences of CWRD related gene families of soybean were analyzed using psRNATarget server^92^ (http://plantgrn.noble.org/psRNATarget/) with default parameters. To confirm the presence of predicted miRNA target sites, we utilized the degradome data set of Song *et al*.^93^ to construct a custom reference database for the multiple sequence alignment using the BLAST algorithm version 2.6.0 on a local standalone command line-based environment^94^. First, we formatted the reference database using the BLAST embedded function of makeblastdb then the query sequences were aligned using the blastn function optimized for short sequences. The statistical significance was pre-set to *E*-value < 10.

### Digital Expression Analysis in soybean plant development, anatomical parts, biotic and abiotic stresses

Publically available microarray data was used to determine the expression of CWRD gene families of soybean in five soybean developmental stages, i.e. germination, main shoot growth, flowering, fruit formation and bean development and 68 anatomical parts. Heat maps were generated in GENEVESTIGATOR (https://genevestigator.com/gv/). We also performed digital expression profiling of CWRD gene families in soybean root hair cell in response to *B. japonicum* inoculation^41^, and *P. sojae* infection for 72 h and 120 h (https://www.ncbi.nlm.nih.gov/geo/query/acc.cgi?acc=GSE7124), in soybean roots under 50 mM NaHCO3 treatment for 3 h, 6 h, and 12 h^95^ and under heat shock treatment from 22 °C to 42 °C^96^.

### Co-expression networks of soybean CWRD related gene families

Co-expression networks of soybean CWRD related gene families were developed by using PlaNet^22, 97^ (http://aranet.mpimp-golm.mpg.de/index.html). To visualize functional association between gene families related to CWRD in soybean, we used FamNet database built in PlaNet (http://aranet.mpimp-golm.mpg.de/famnet.html). Briefly, Pfam labels of each gene family were used as a query to access the label specific networks and then a single probeset was select to redirect to co-expression network and ontology analysis. The networks containing all nodes supported by ELA and all genes two steps away from the query gene were drawn and exported along with the tables containing annotations and labels of genes found in co-expression networks^98^. We considered gene networks of a single query gene having maximum co-occurrences in order to expand our understanding about co-expressed CWRD gene families of soybean.

### Data availability statement

All data generated or analyzed during this study are included in this published article (and its Supplementary Information files).

## Electronic supplementary material

Supplementary material 2

Supplementary material 1
