# Supplementary material for: Systems Identification and Characterization of Cell Wall Reassembly and Degradation Related Genes in Glycine max (L.) Merill, a Bioenergy Legume
Source: Sci Rep. 2017 Sep 7;7:10862. doi: 10.1038/s41598-017-11495-4 (PMC5589831; doi:10.1038/s41598-017-11495-4)
Supplement: Supplementary file 1 — Supplementary material 2 [file 41598_2017_11495_MOESM1_ESM.doc]

**Systems Identification and Characterization of Cell Wall Reassembly and Degradation Related Genes in *Glycine max* (L.) Merill, a Bioenergy Legume**

**Muhammad Amjad Nawaz^1^, Hafiz Mamoon Rehman^1^, Muhammad Imtiaz^2^, Faheem Shehzad Baloch^3^, Jeong Dong Lee^4^, Seung Hwan Yang^1^, Soo In Lee^5*^, Gyuhwa Chung^1*^**

^1^Department of Biotechnology, Chonnam National University, Chonnam 59626, Republic of Korea

^2^School of Environmental Science and Engineering, Guangzhou University, Guangzhou 510275, China.

^3^Department of Field Crops, Faculty of Agricultural and Natural Science, Abant Izzet Baysal University, 14280 Bolu, Turkey

^4^Division of Plant Biosciences, Kyungpook National University, Daegu 702-701, Republic of Korea

^5^Metabolic Engineering Division, Department of Agricultural Biotechnology, National Academy of Agricultural Science (NAAS), Jeonju 560-500, Republic of Korea

***Corresponding author(s)**

Gyuhwa Chung

[chung@chonnam.ac.kr](mailto:chung@chonnam.ac.kr)

Co-corresponding author

Soo In Lee

[silee@korea.kr](mailto:silee@korea.kr)

**Supplementary figures**

**Supplementary figure S1. Synteny analysis of CWRD related gene families in soybean.** Figure shows synteny within members of each soybean CWRD gene family. (A) Expansins, (B) yieldins, (C) xyloglucan endotransglucosylases/hydrolases, (D) endo-1,4-β-glucanases, (E) endo-xylanases, (F) glucan 1,3- β-glucosidases, (G) polygalacturonases, (H) β –galactosidases, (I) Pectin acetyl esterases, (J) Pectate and pectin lyases, (K) Rhamnogalacturonana l lyases and (L) Pectin methyl esterases. Inside the circle, ribbons represent local alignments based on bit score, red (> 80%), orange (> 60%), green (> 40%) and blue (>20%). Ribbon width is correlated with % identity. Ribbons representing best hits are outlined and placed on top of all other ribbons. Histogram on the top of the ideograms, shows how many times each colour has hit the specific part of the sequence.


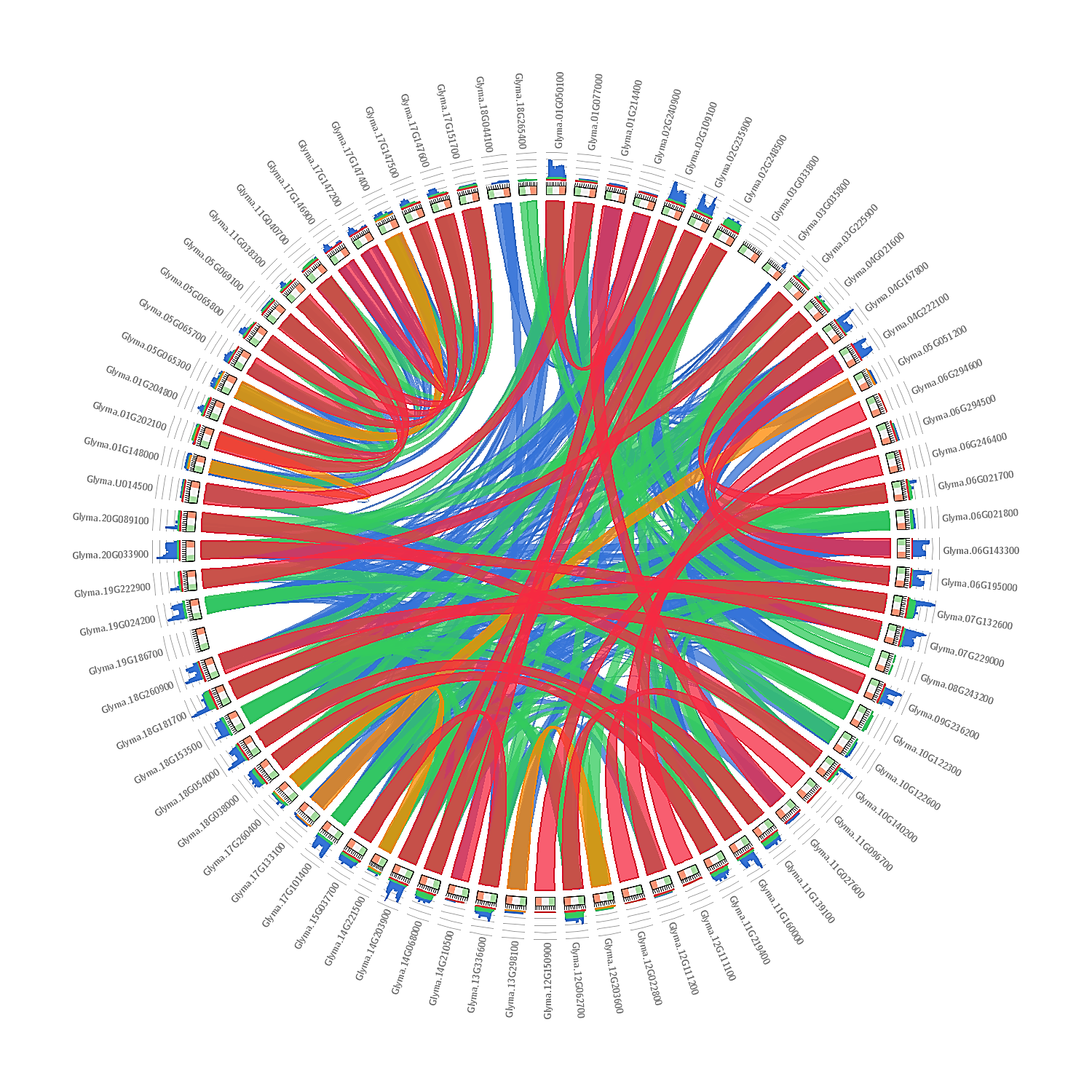


**A**


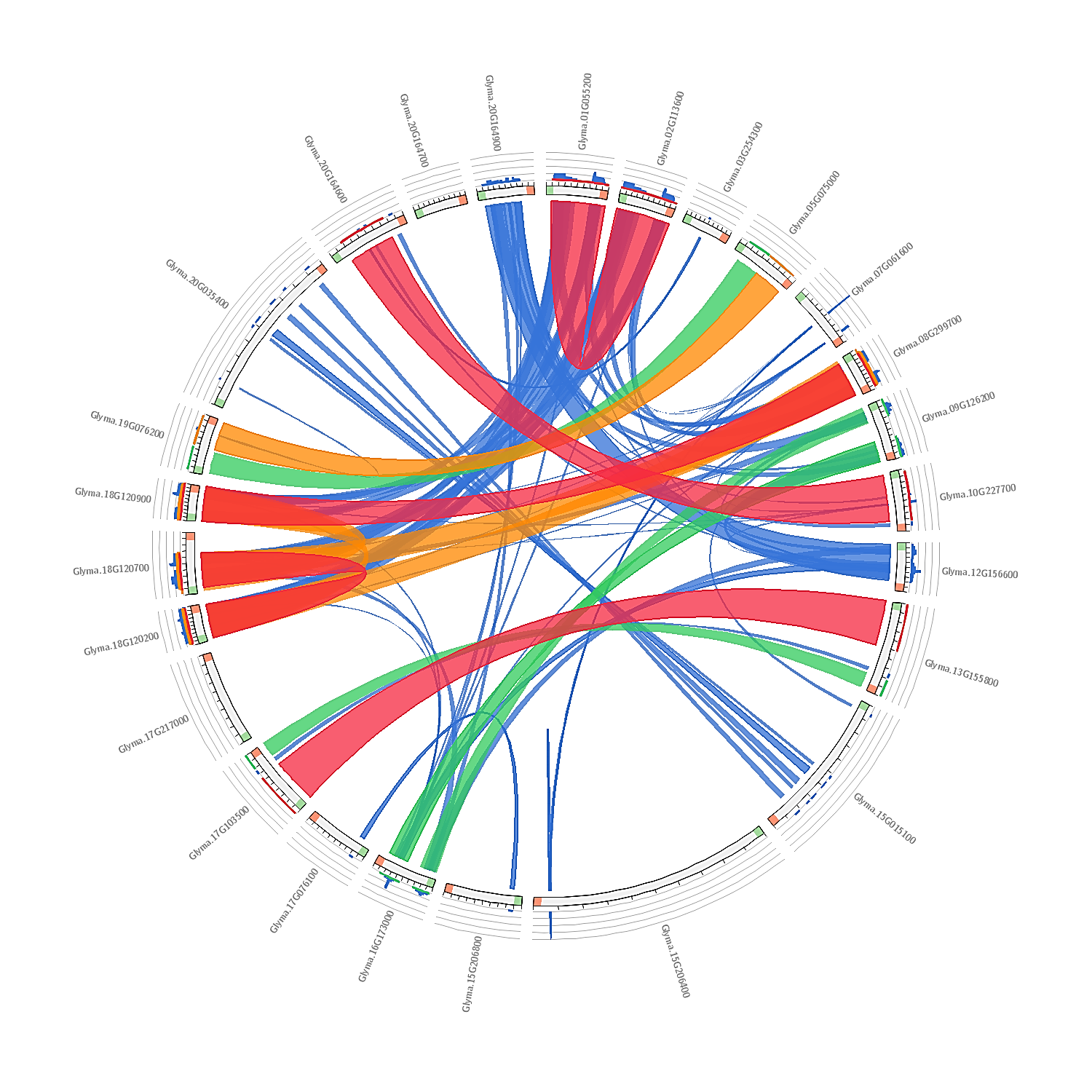


**B**


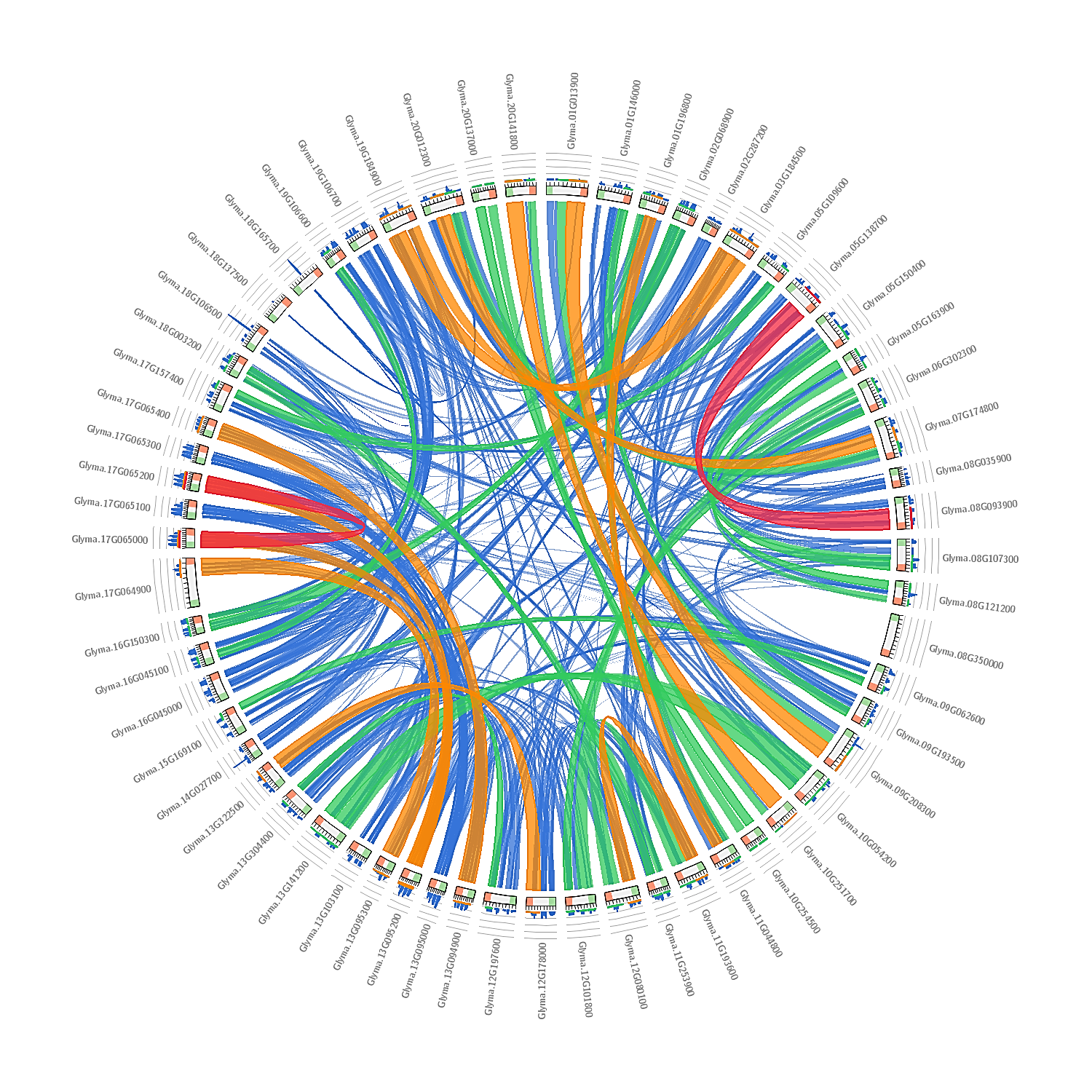


**C**


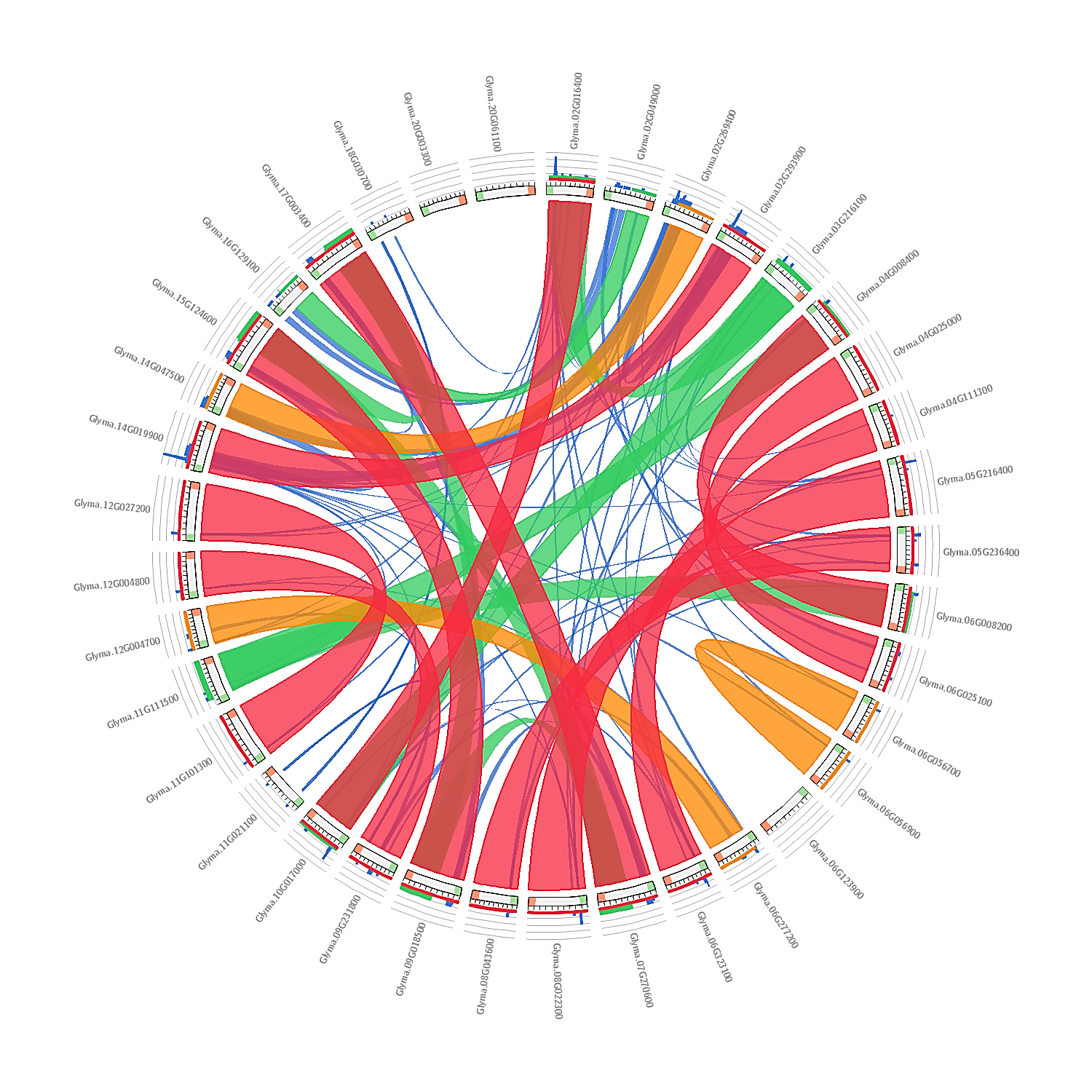


**D**


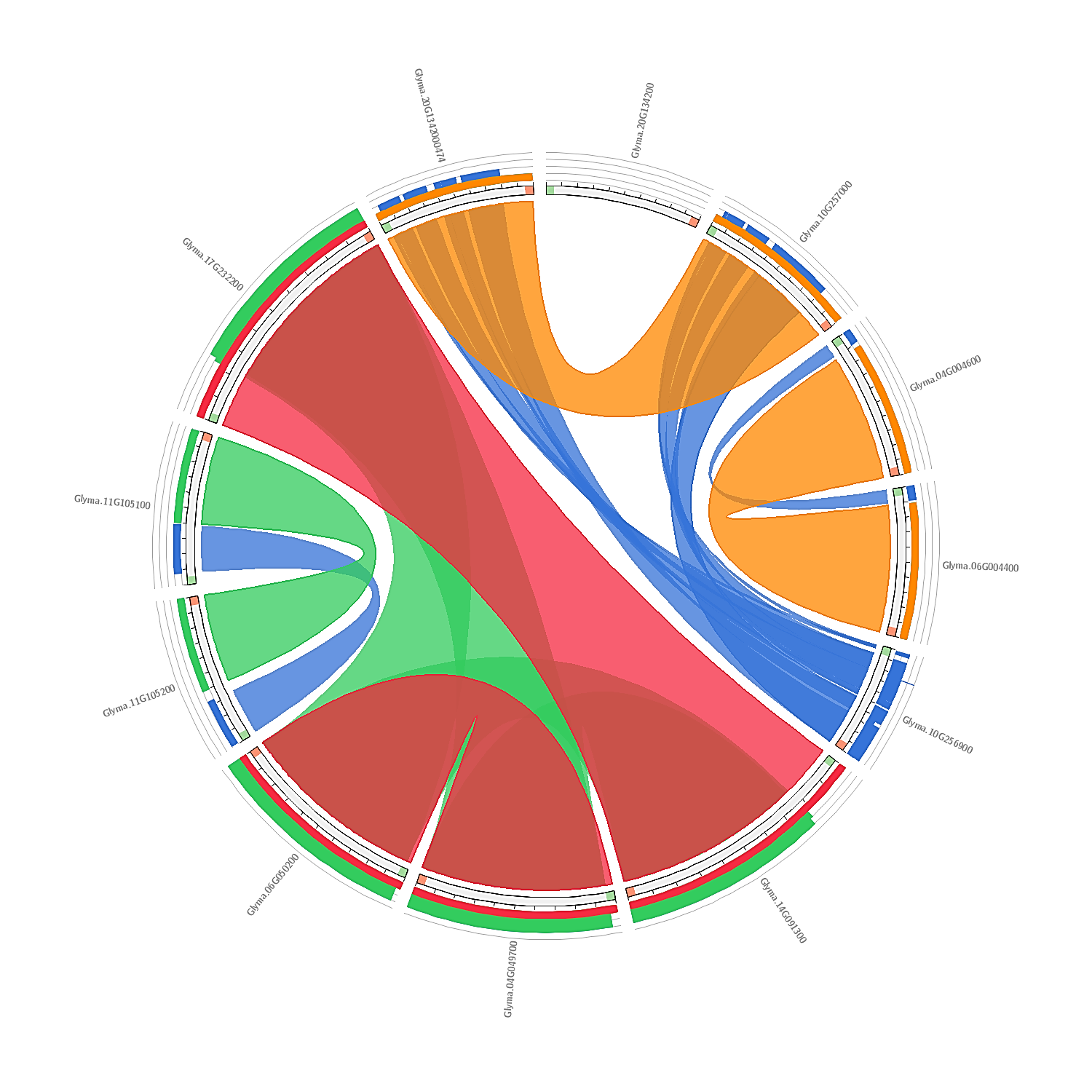


**E**


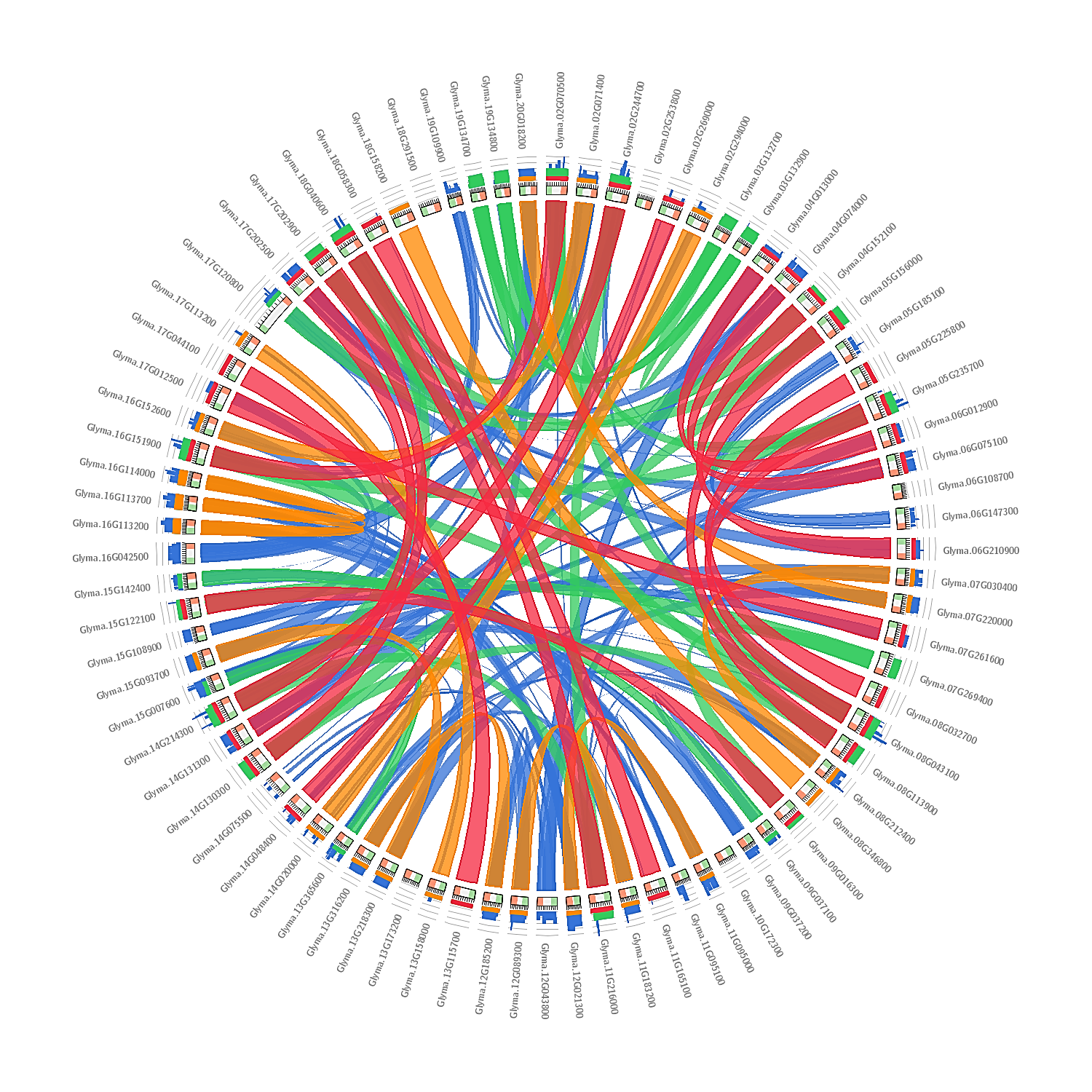


**F**


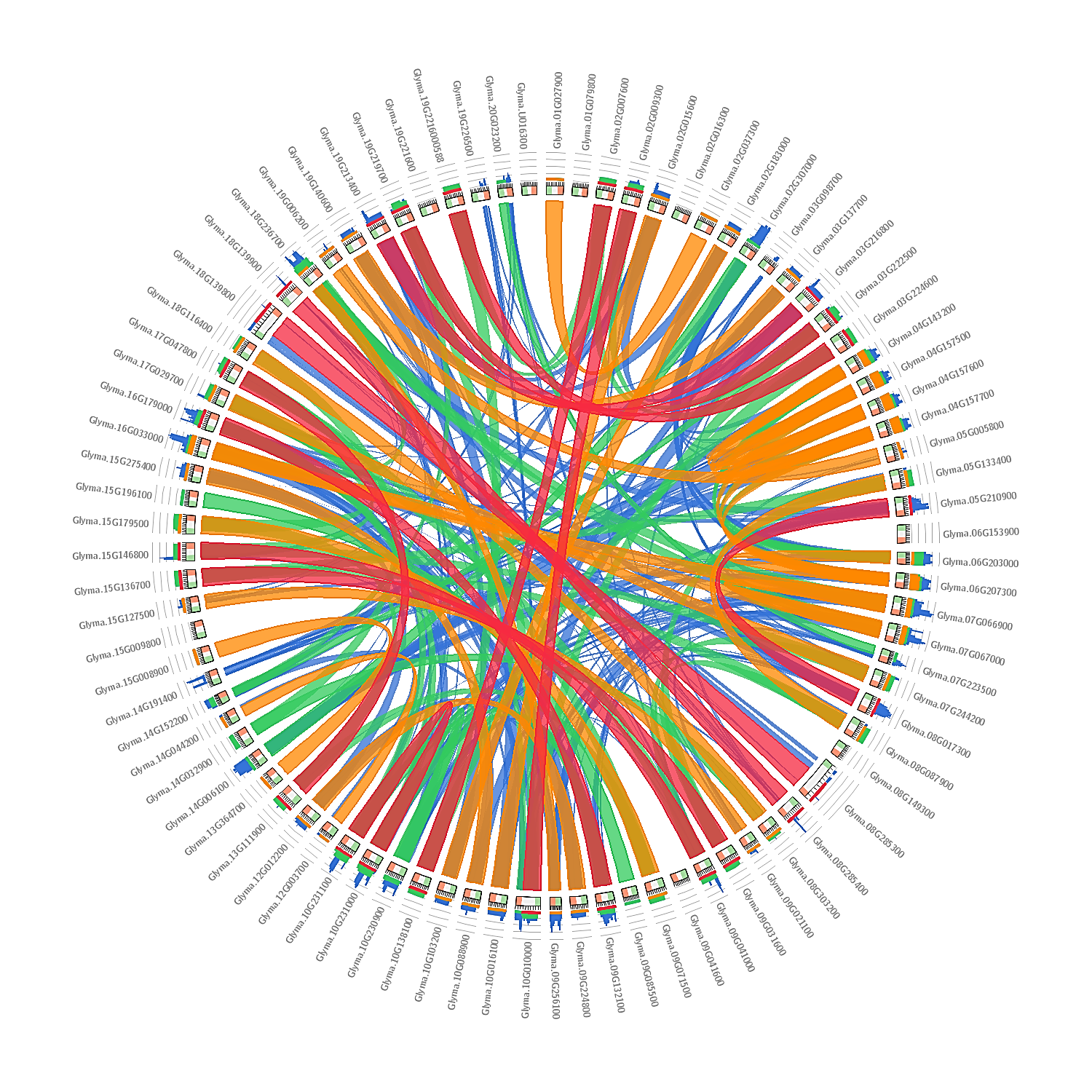


**G**


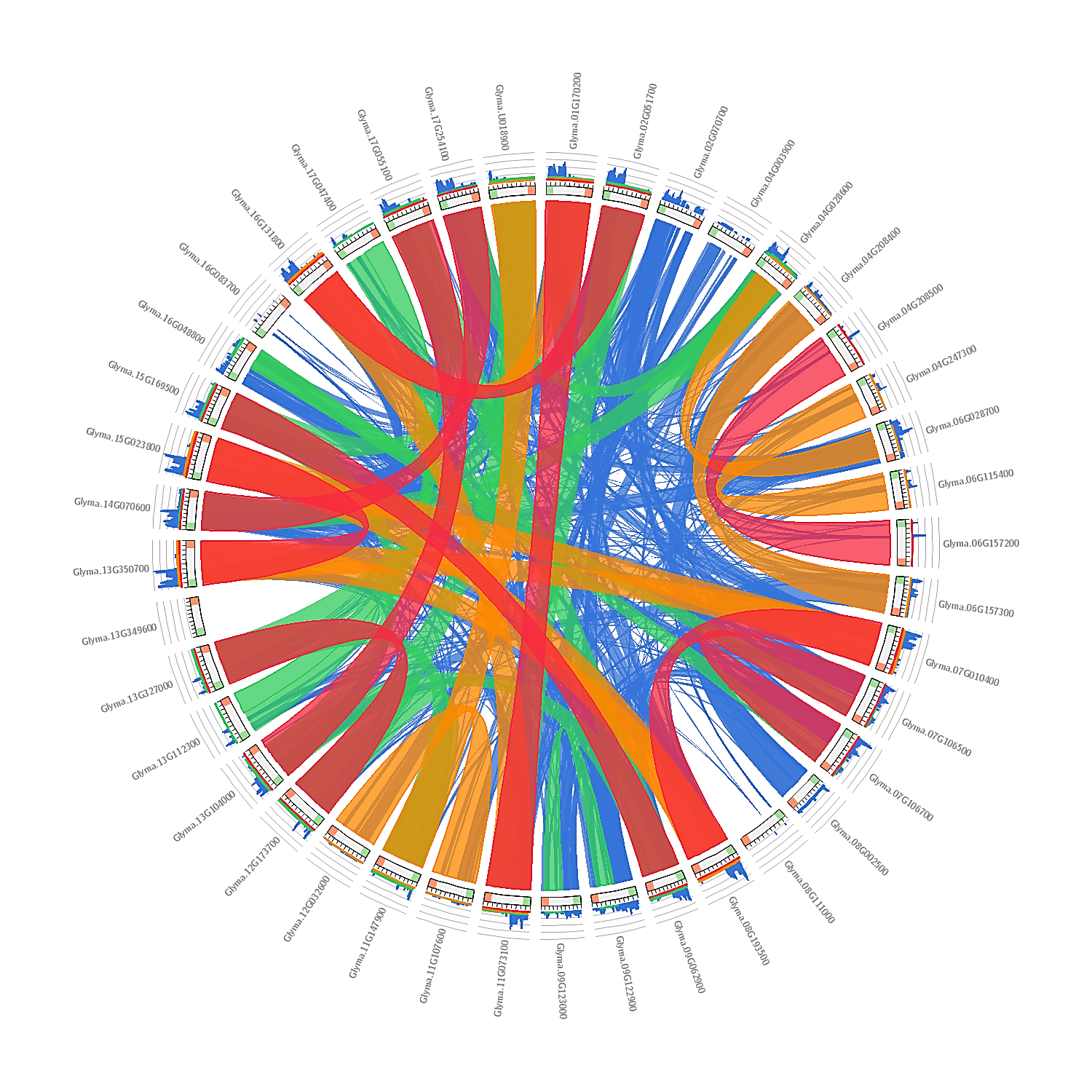


**H**


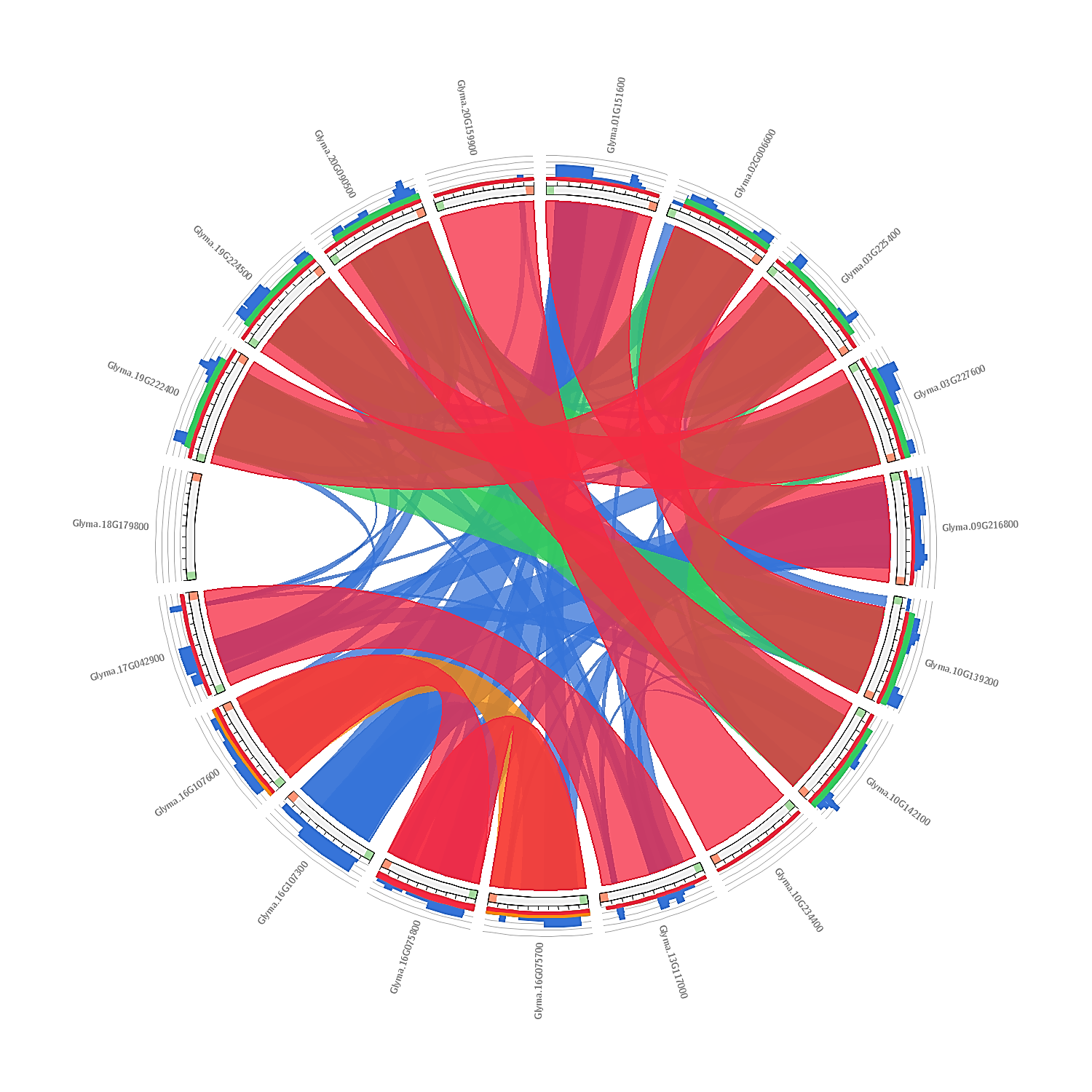


**I**


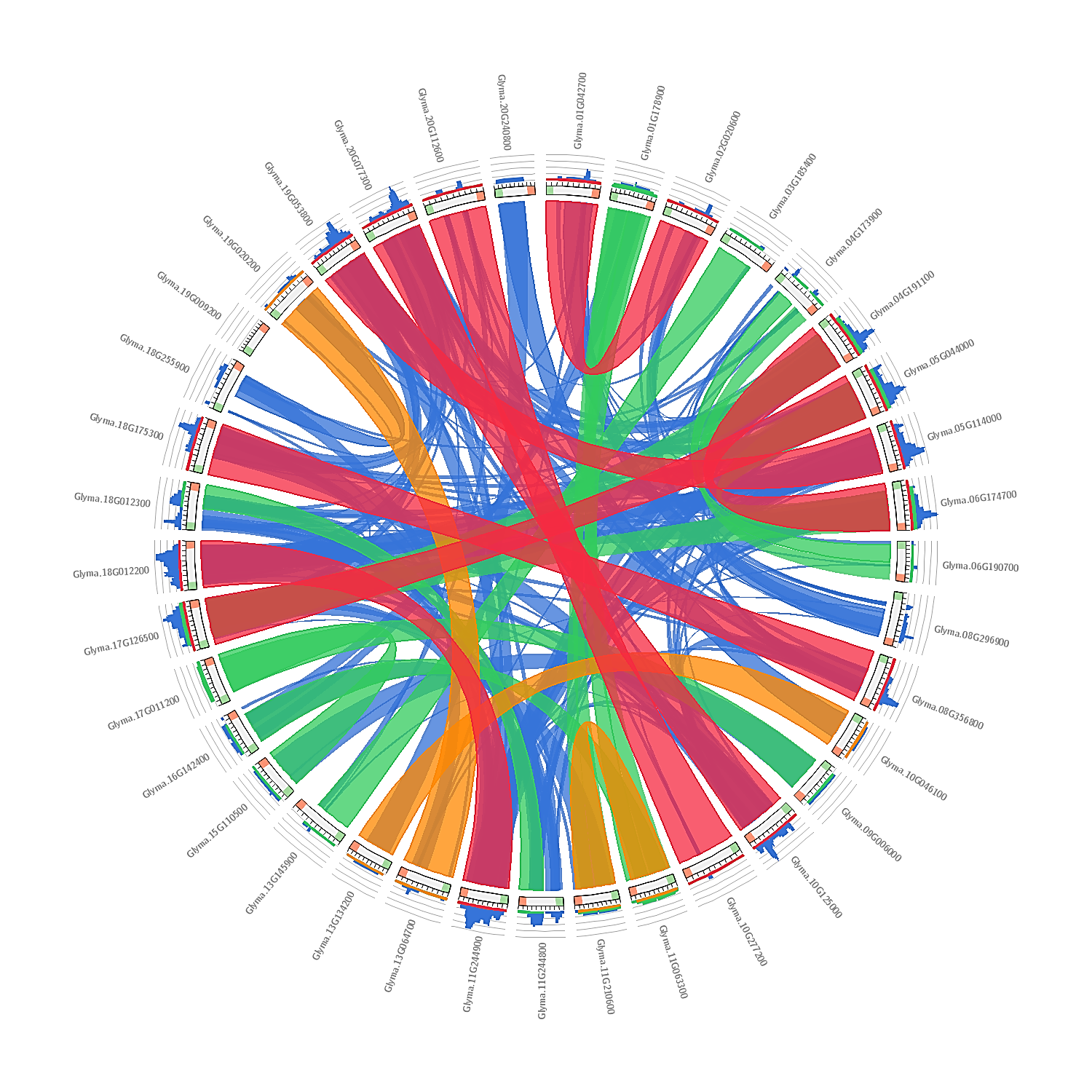


**J**


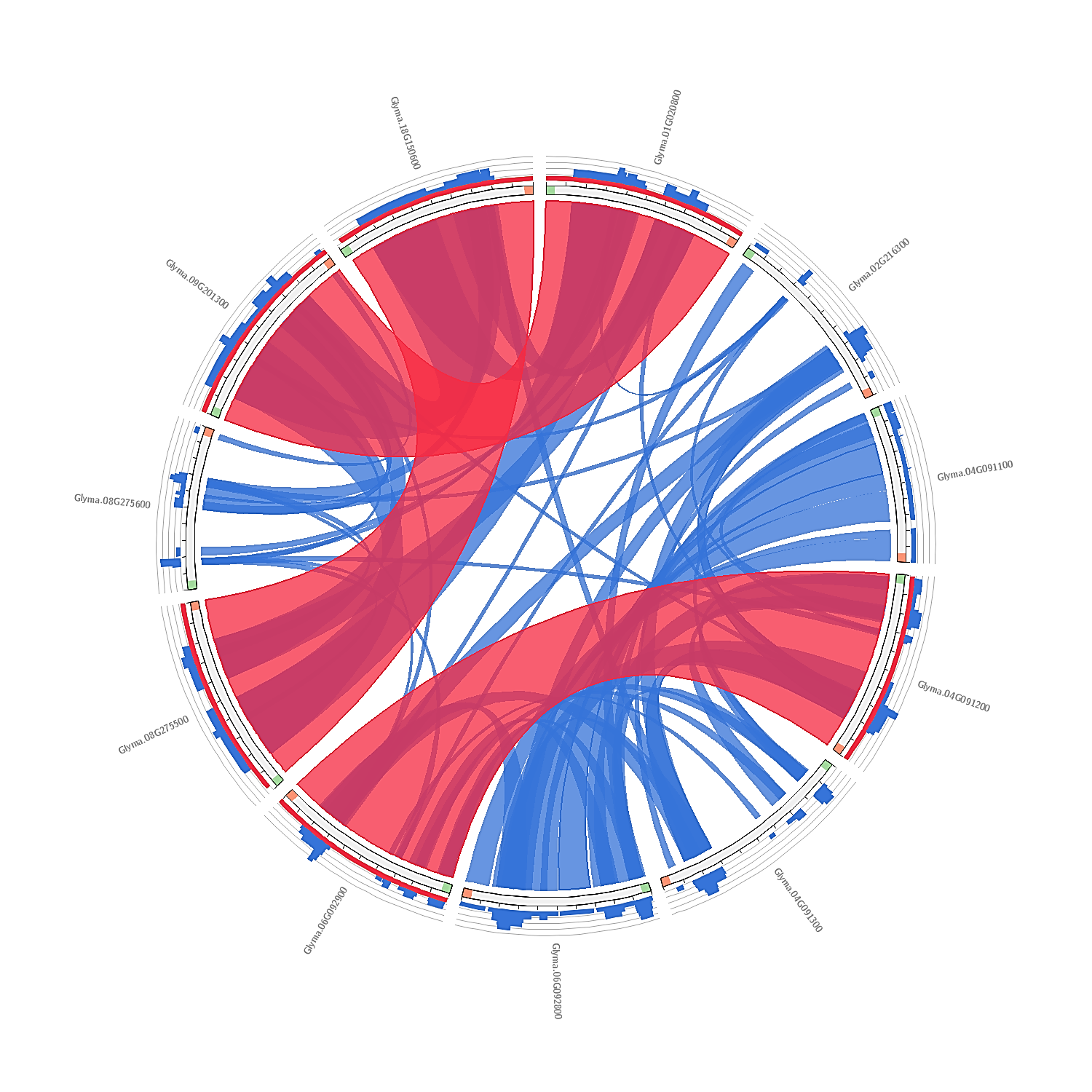


**K**


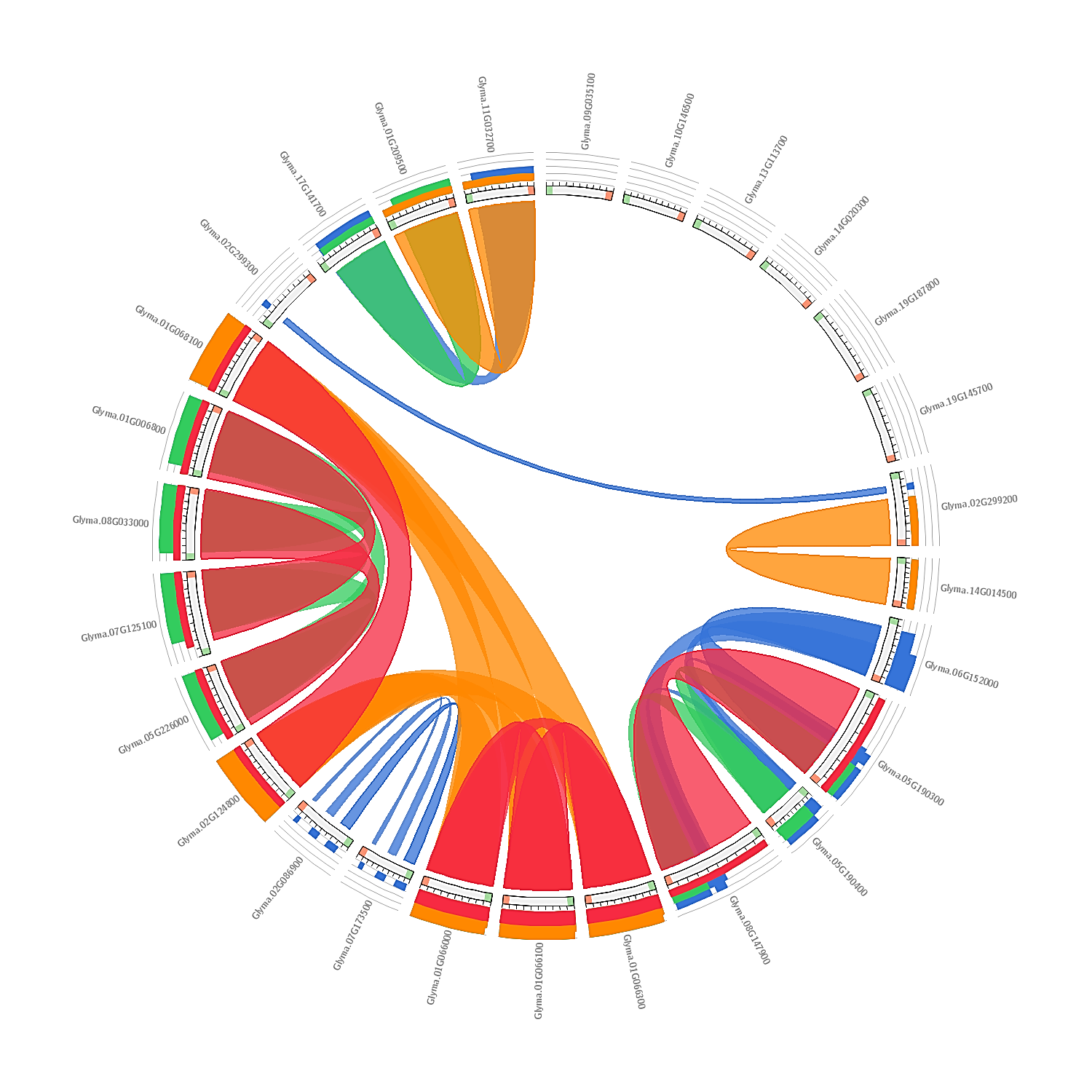


**L**

**Supplementary figure S2. Expression potential of soybean CWRD genes in soybean anatomical parts.** (A) Expression potential of cell wall loosening related gene family members in 68 soybean plant anatomical parts, (B) expression potential of glycoside hydrolase gene families members in 68 soybean plant anatomical parts, and (C) expression potential of pectin modifying related gene family members in 68 soybean plant anatomical parts


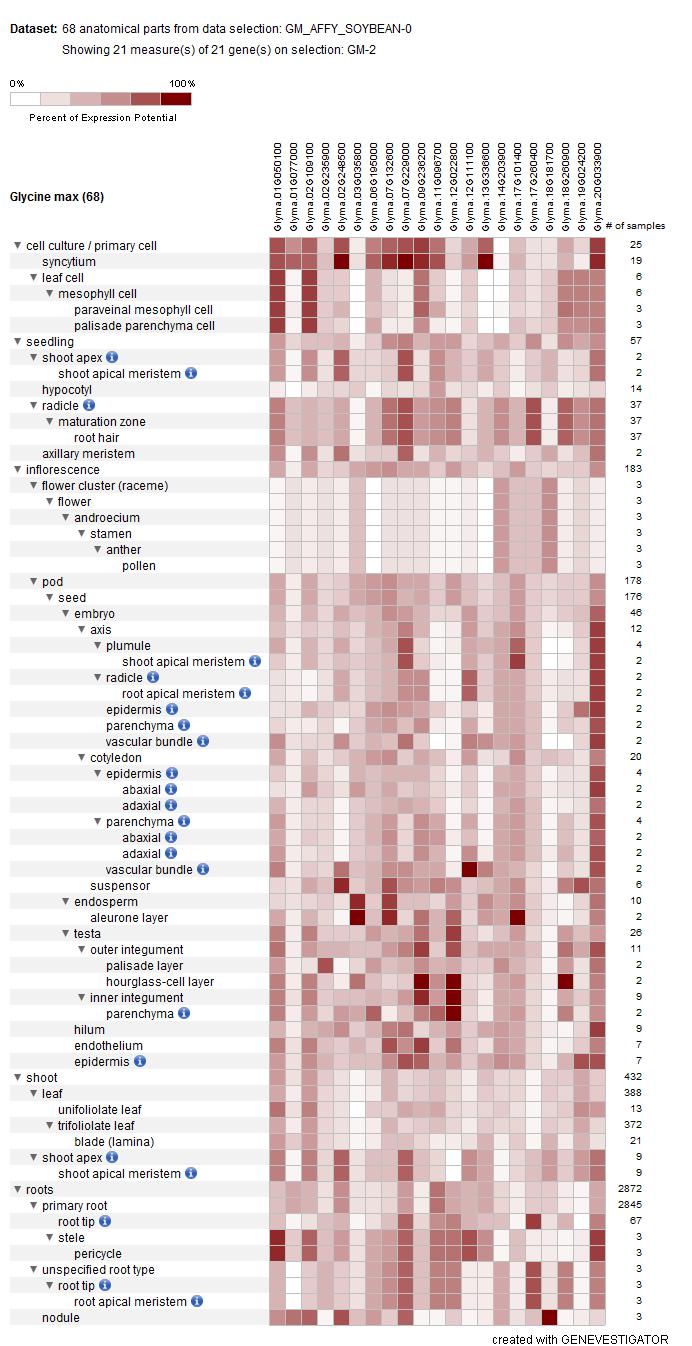


A(i) Expansins


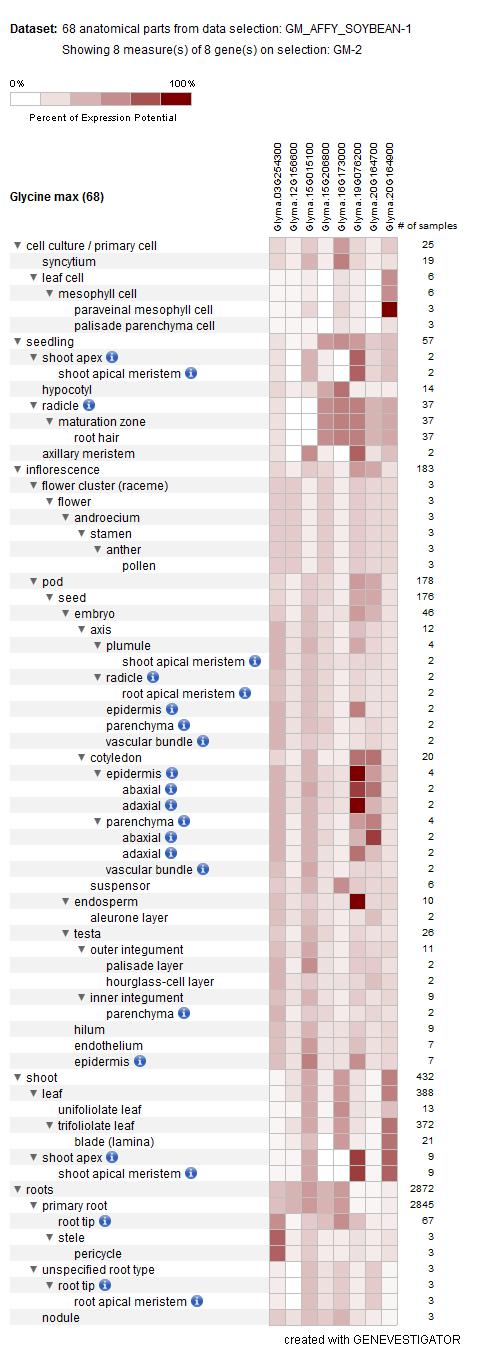


A(ii) Yieldins


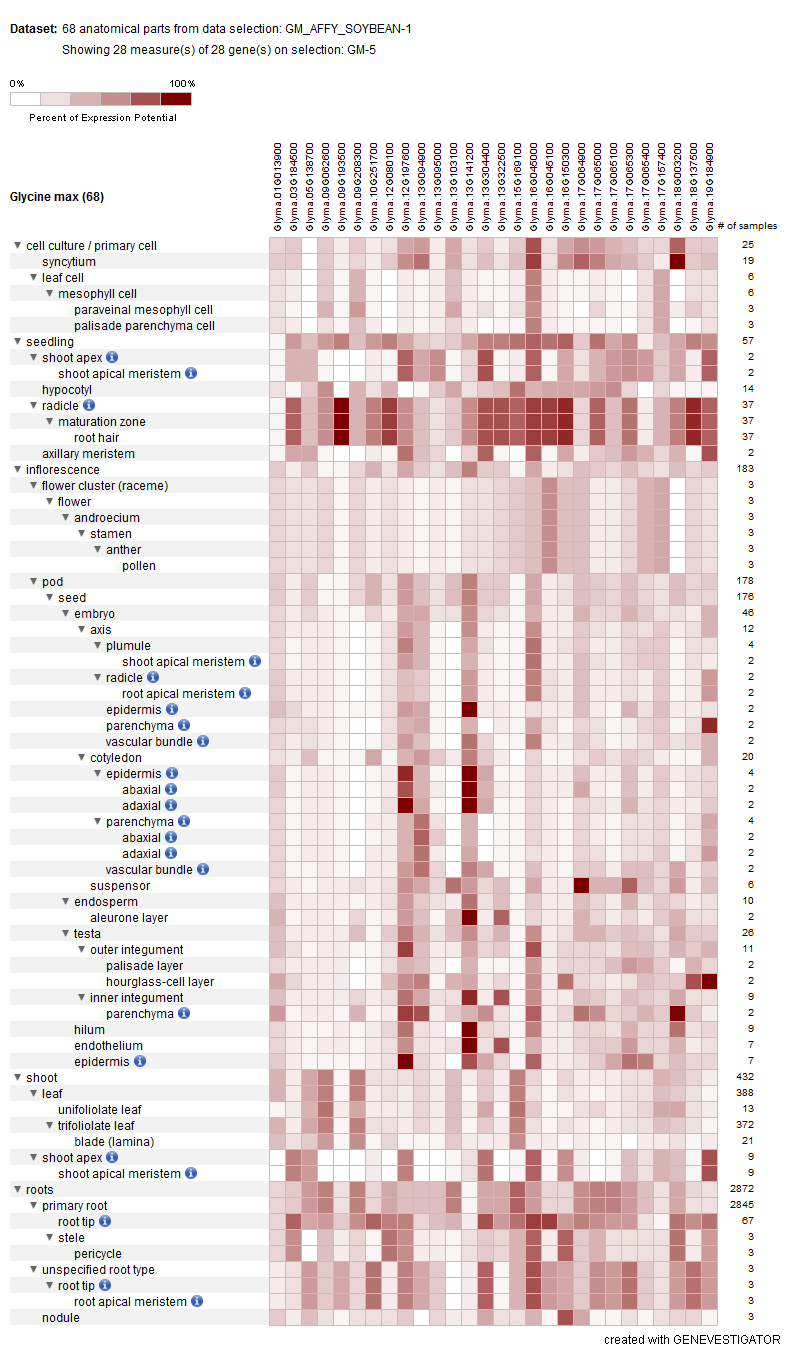


A(iii) Xyloglucan endotransglucosylases/hydrolases


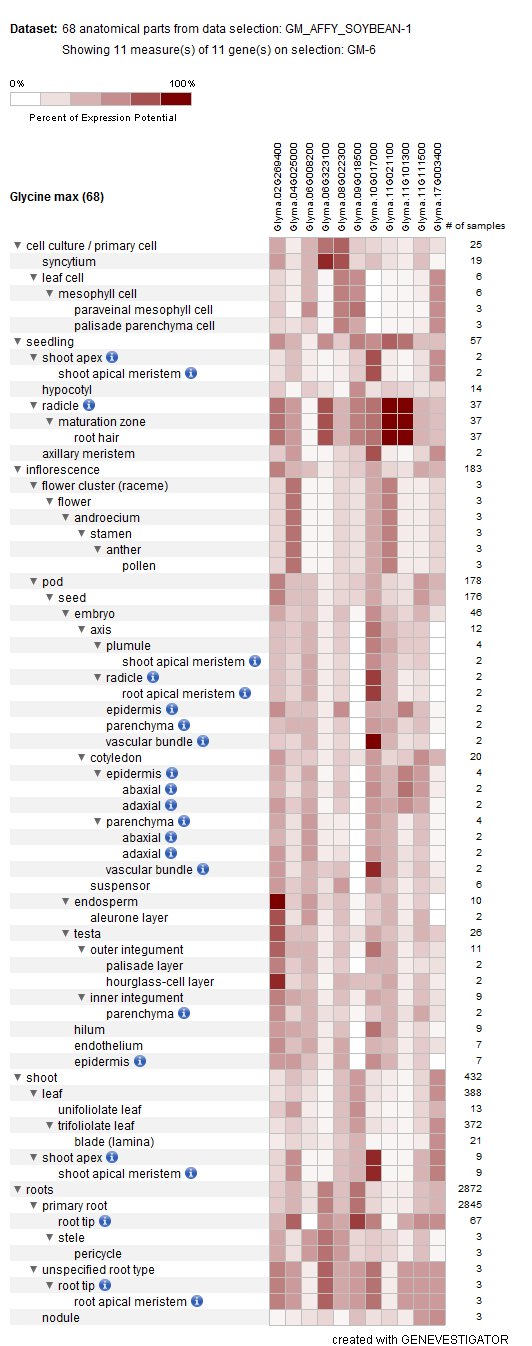


B(i) Endo-1,4-β-glucanases


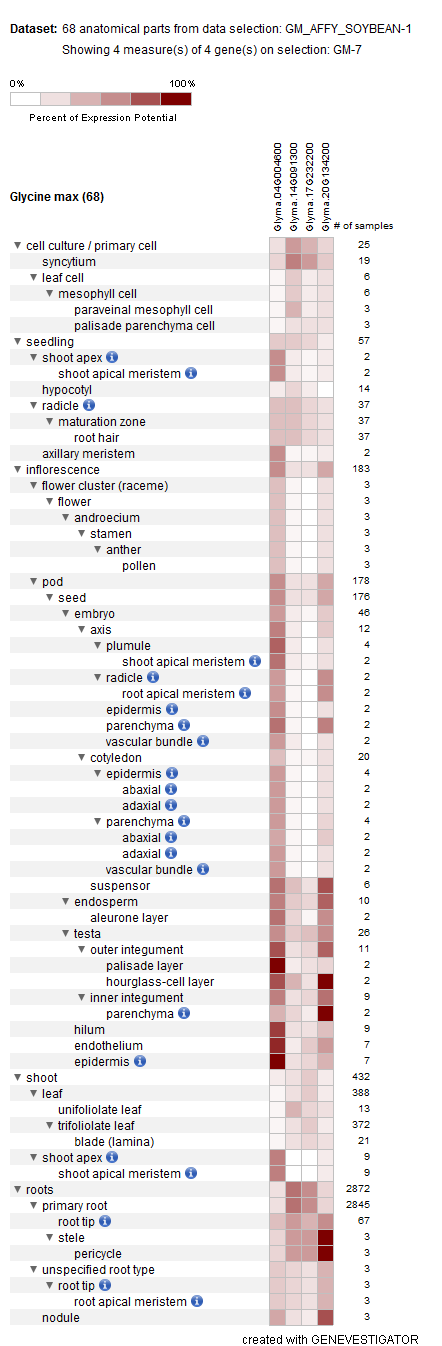


B(ii) Endo-xylanases


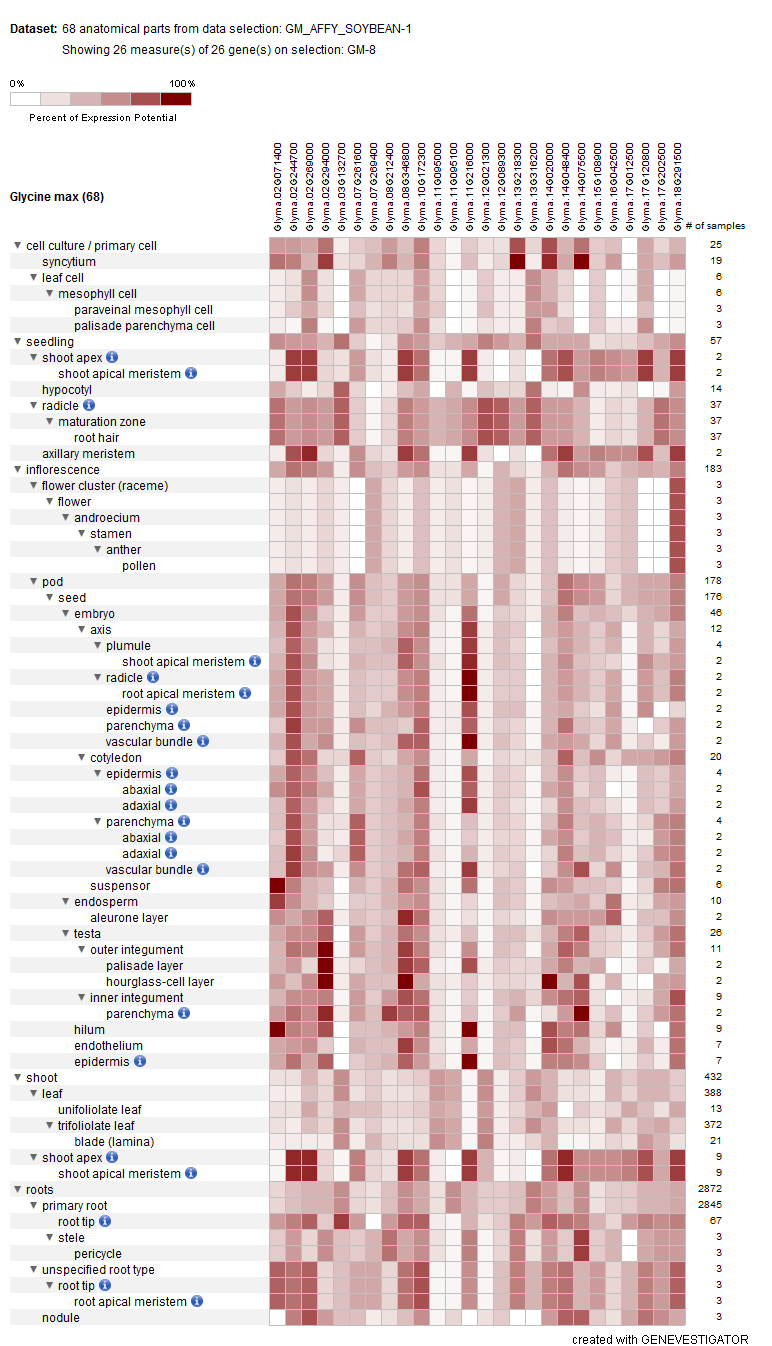


B(iii) Glucan 1,3- β-glucosidases


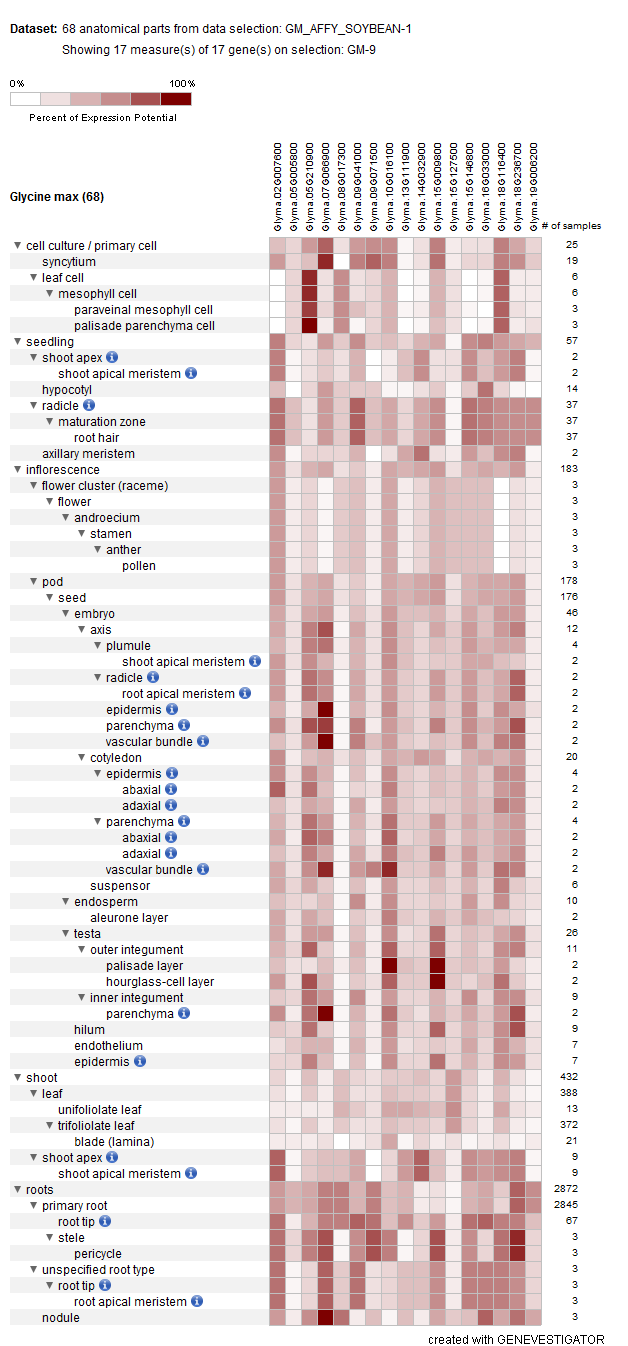


B(iv) Polygalacturonases


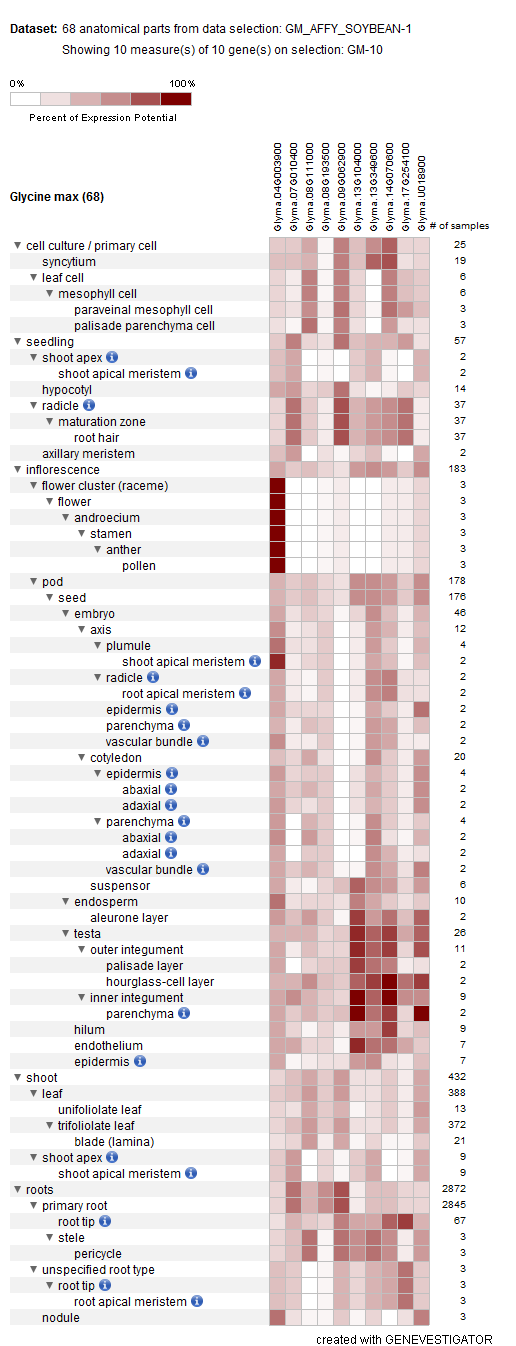


B(v) β -Galactosidases


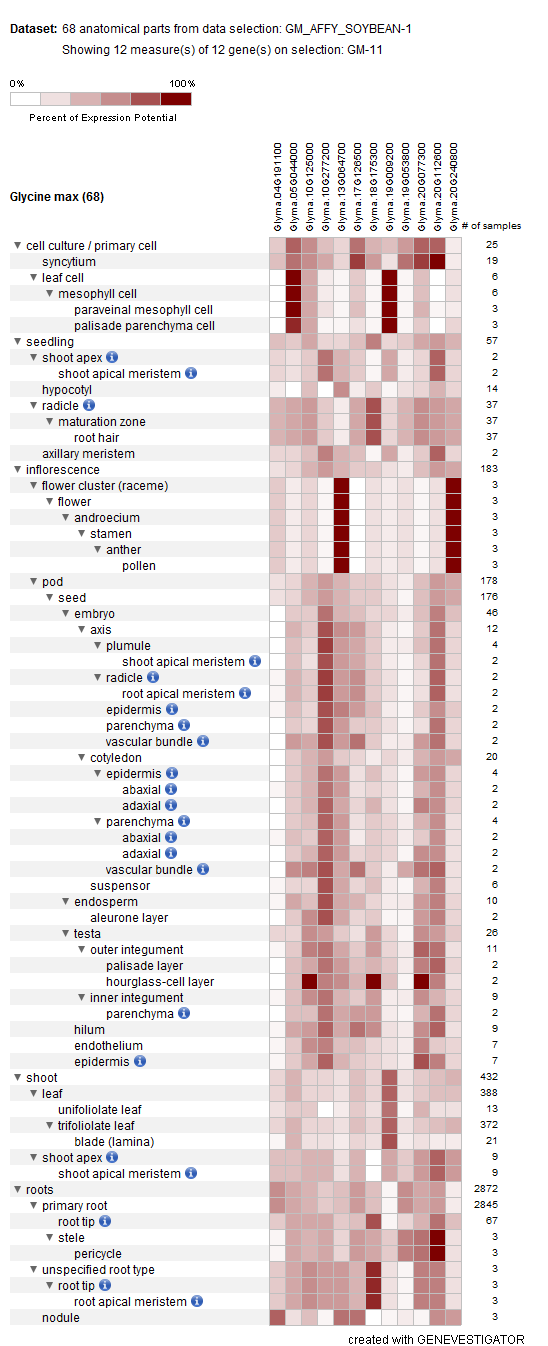


C(i) Pectate and pectin lyases


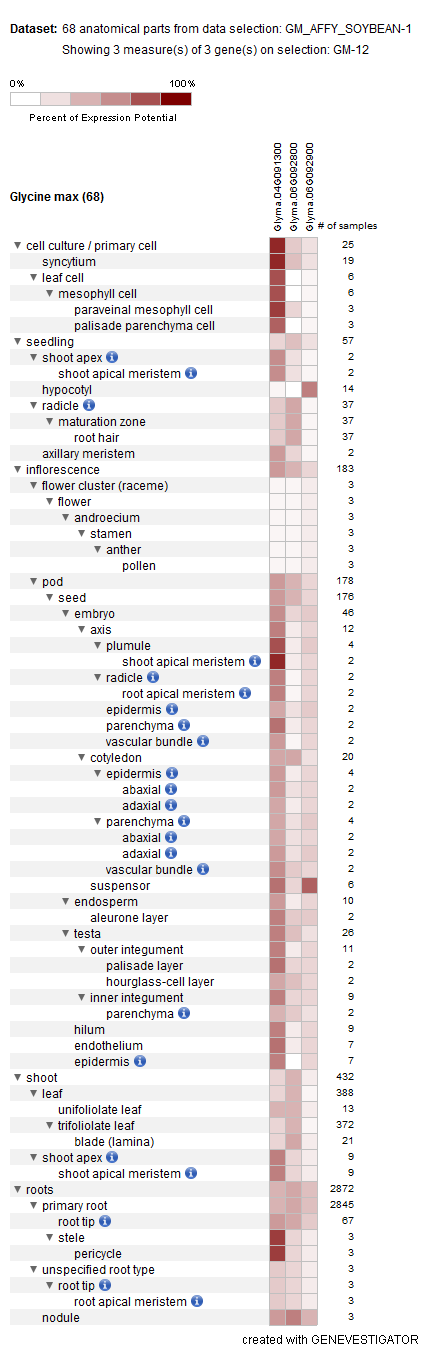


C(ii) Rhamnogalacturonana l lyases


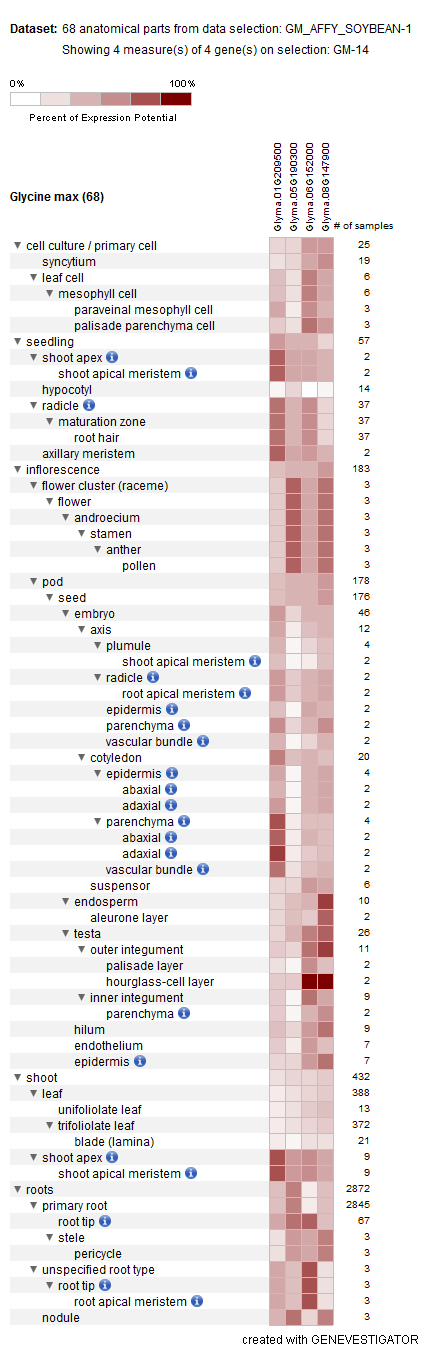


C(iii) Pectin methyl esterases


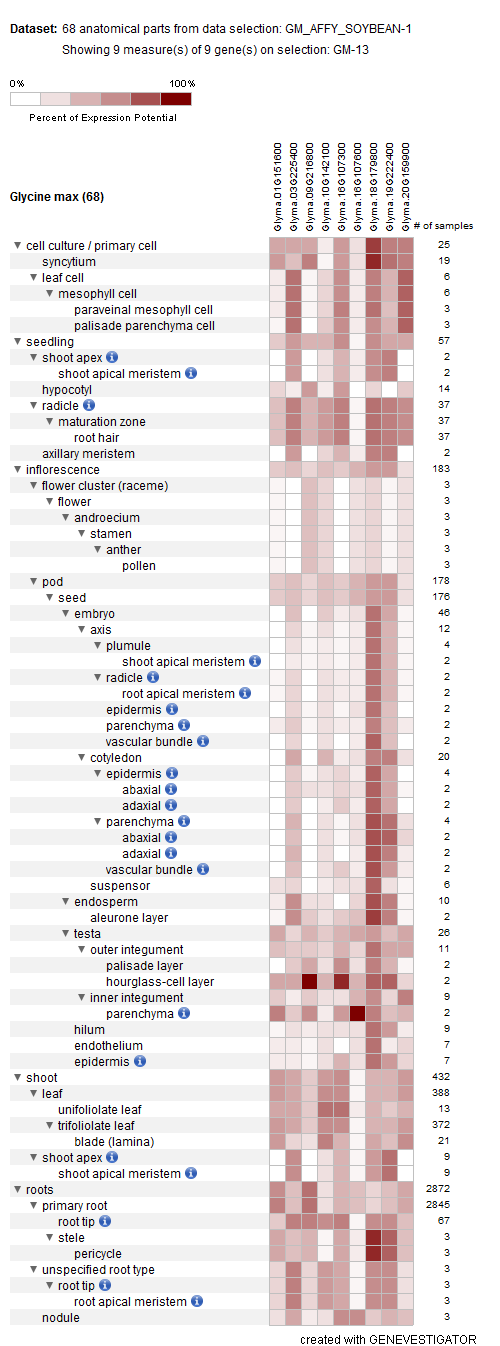


C(iv) Pectin acetyl esterases

**Supplementary figure S3. Expression potential of soybean CWRD related gene family members in biotic and abiotic stresses**. (A) Heat shock, (B) NaHCO_3_ treatment, (C) *Bradyrhizobium japoniucum* inoculation and (D) *Phytophthora sojae* inoculation.


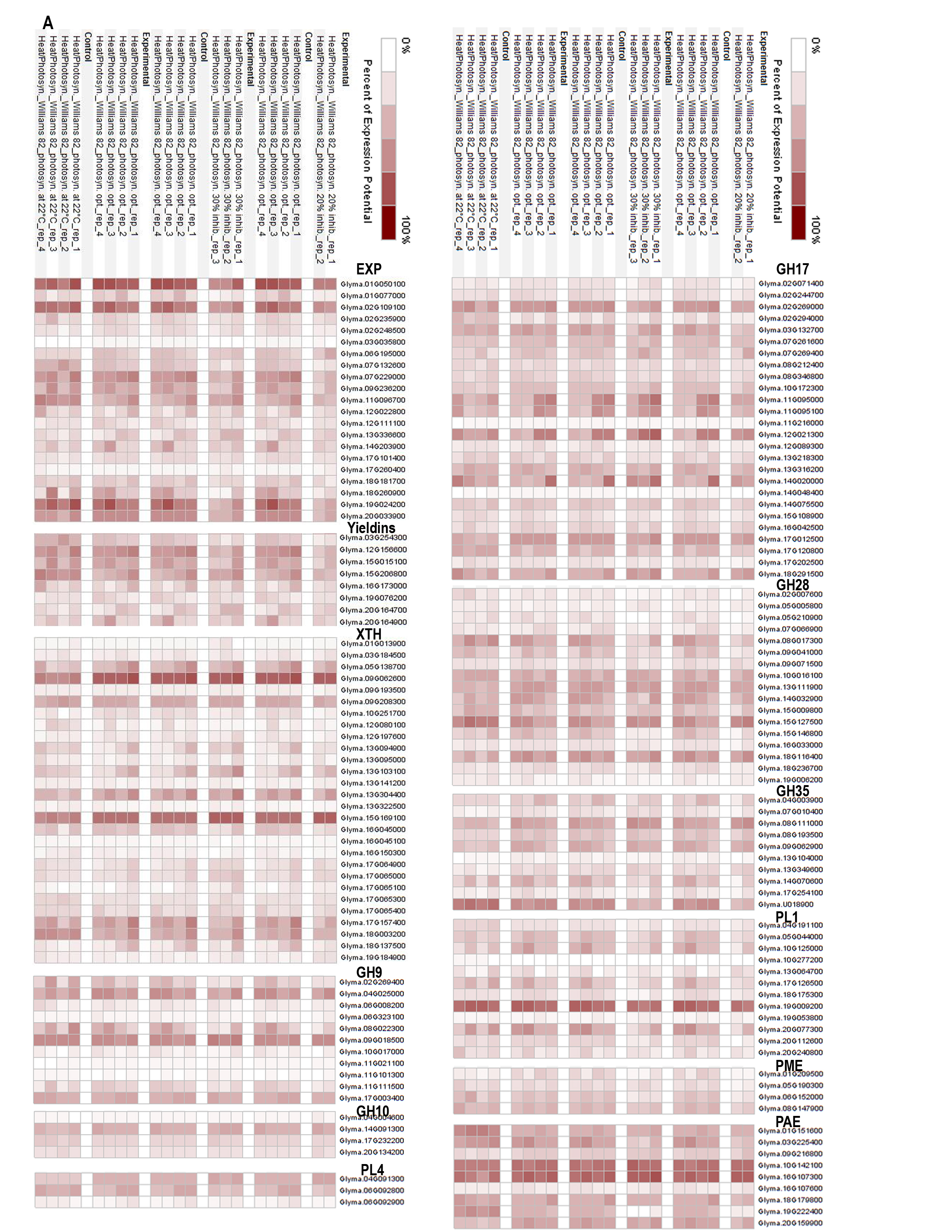


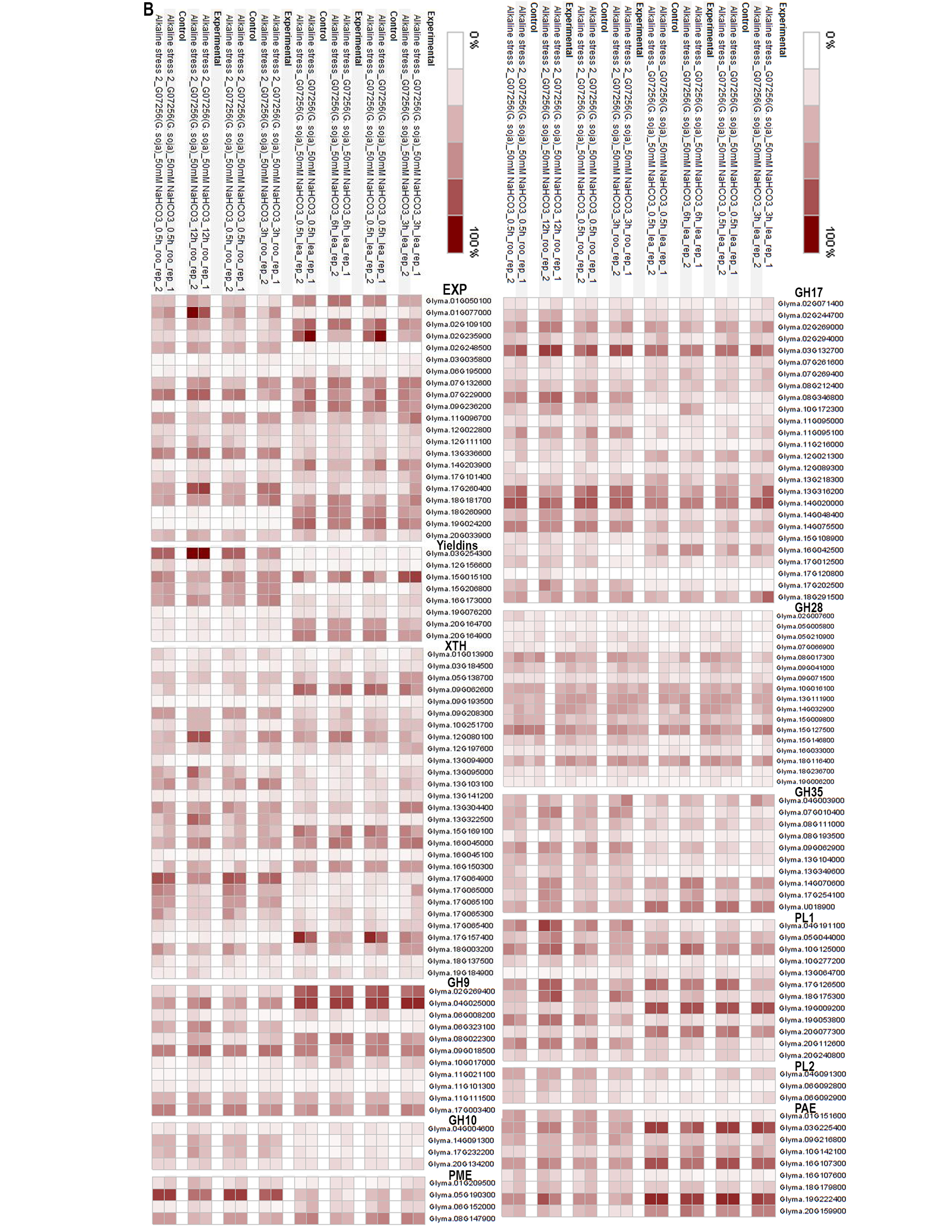


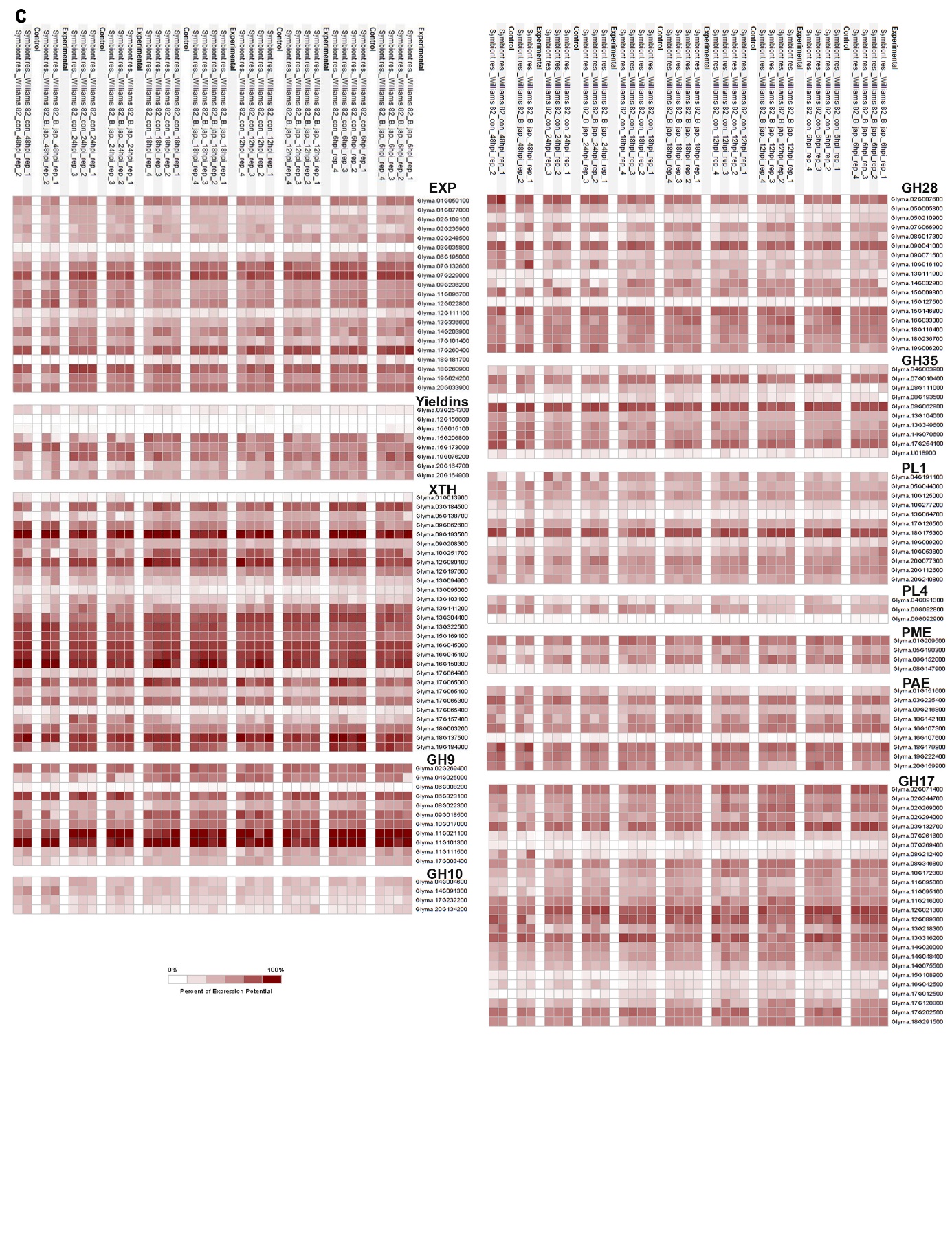


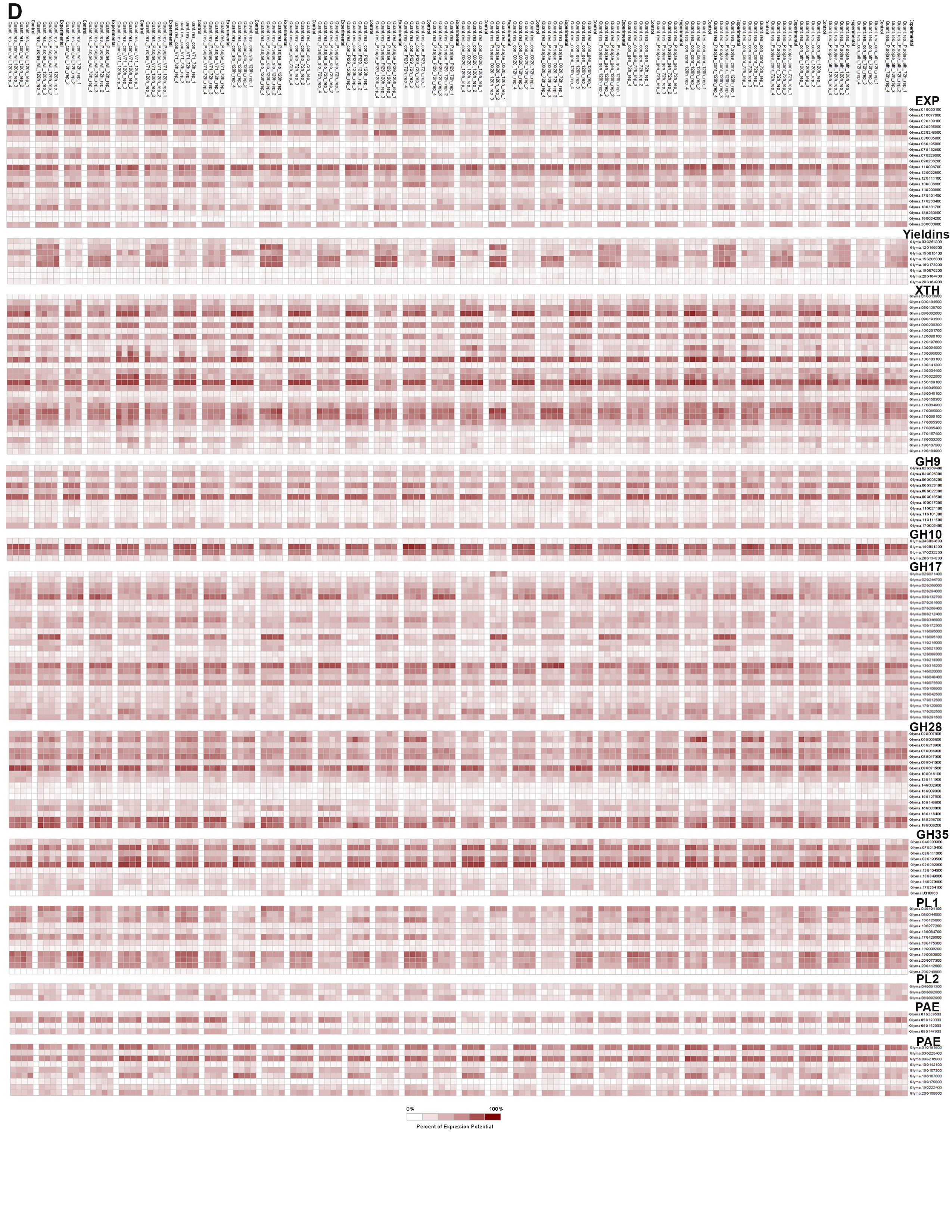


**Supplementary figure S4. Co-expression network neighbourhood of CWRD related gene family members.** The networks contain the query gene (large node) and all genes two steps away from the query genes. Figure shows eleven gene families except rhamnogalacturonana l lyases Node shapes and colours represent label co-occurrences. (A) Expansins, (B) yieldins, (C) xyloglucan endotransglucosylases/hydrolases, (D) endo-1,4-β-glucanases, (E) endo-xylanases, (F) glucan 1,3- β-glucosidases, (G) polygalacturonases, (H) β –galactosidases, (I) pectate and pectin lyases, (J) pectin acetyl esterases, and (K) pectin methyl esterases.

**
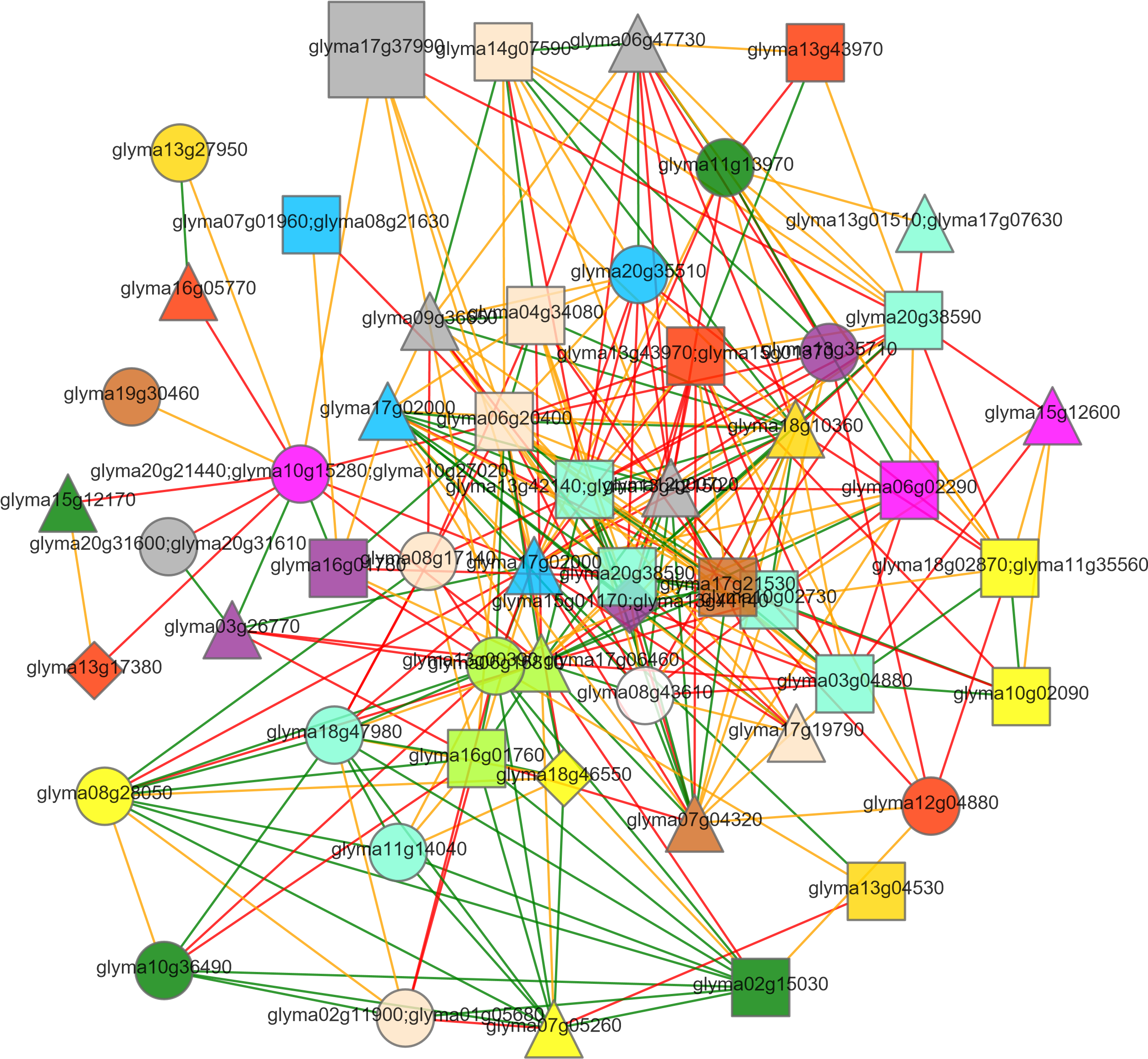
**

**
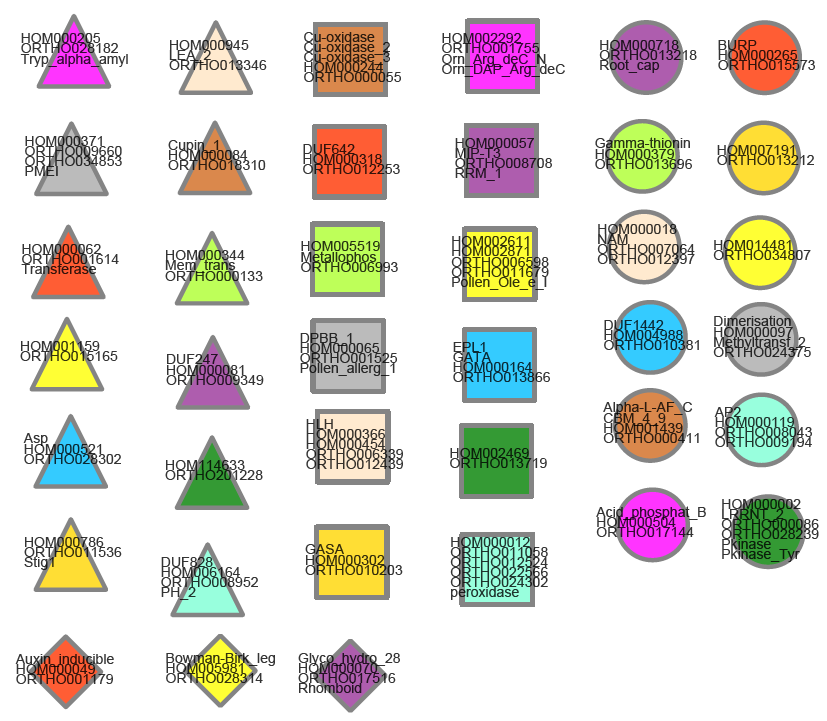
**

**A**

**
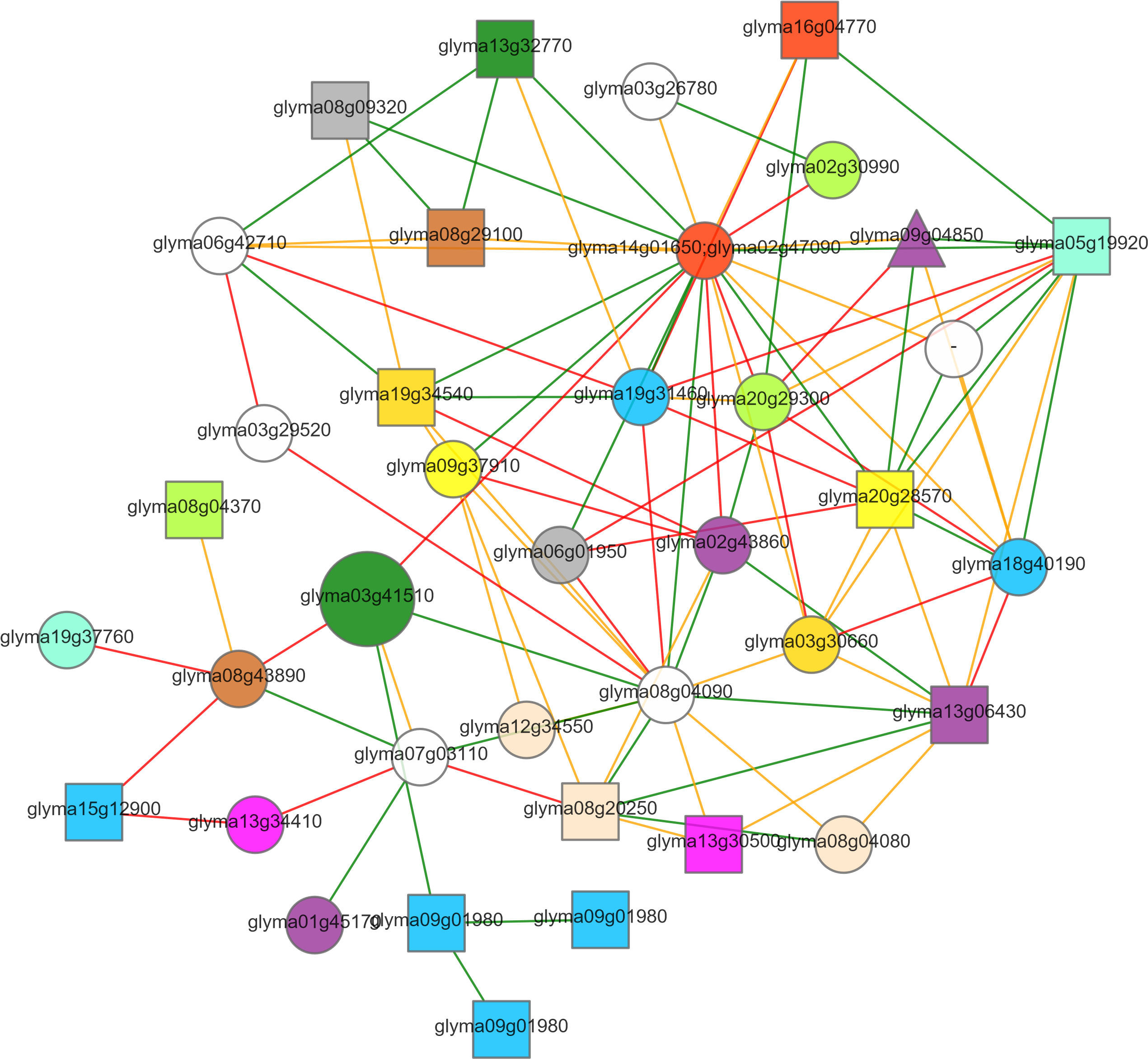
**

**
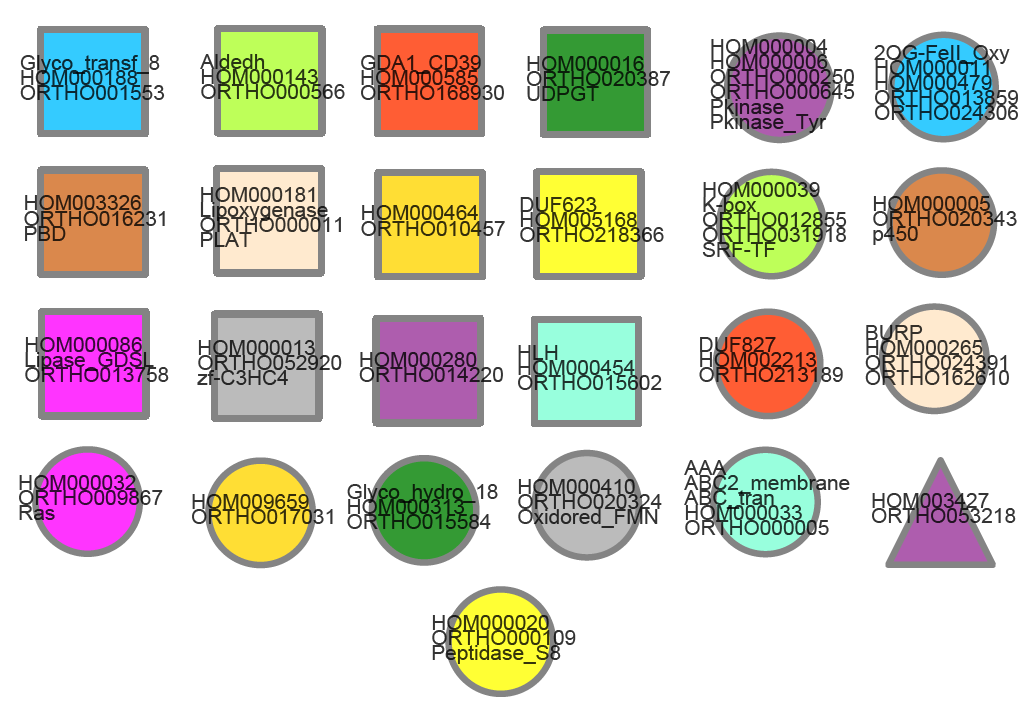
**

**B**

**
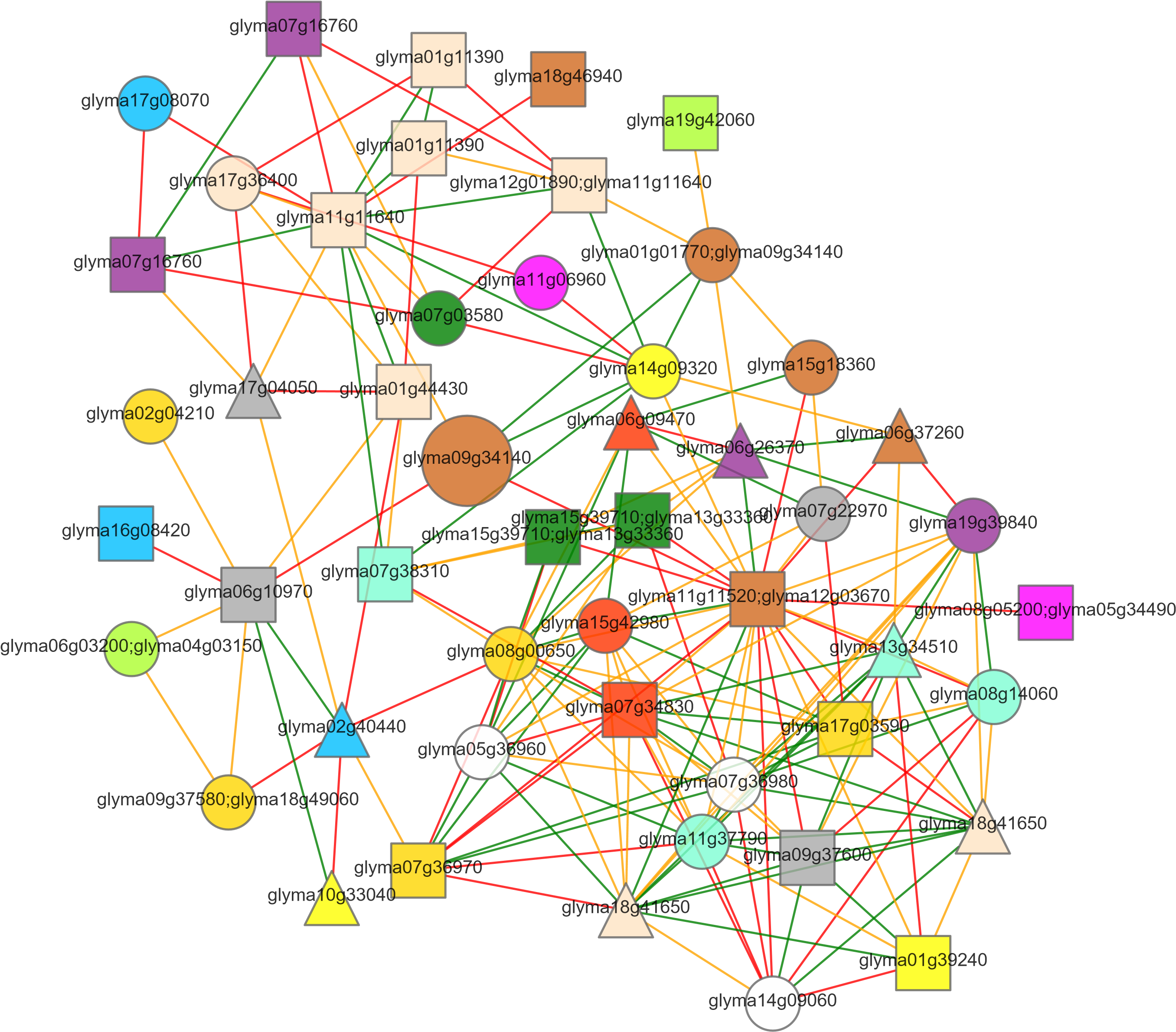
**

**
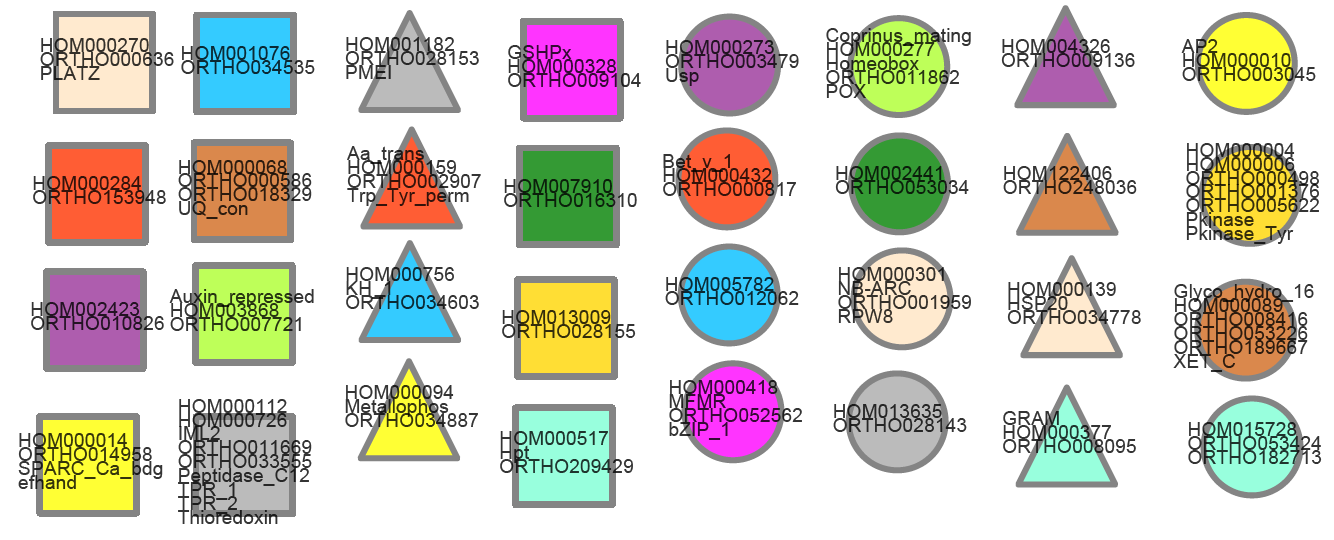
**

**C**

**
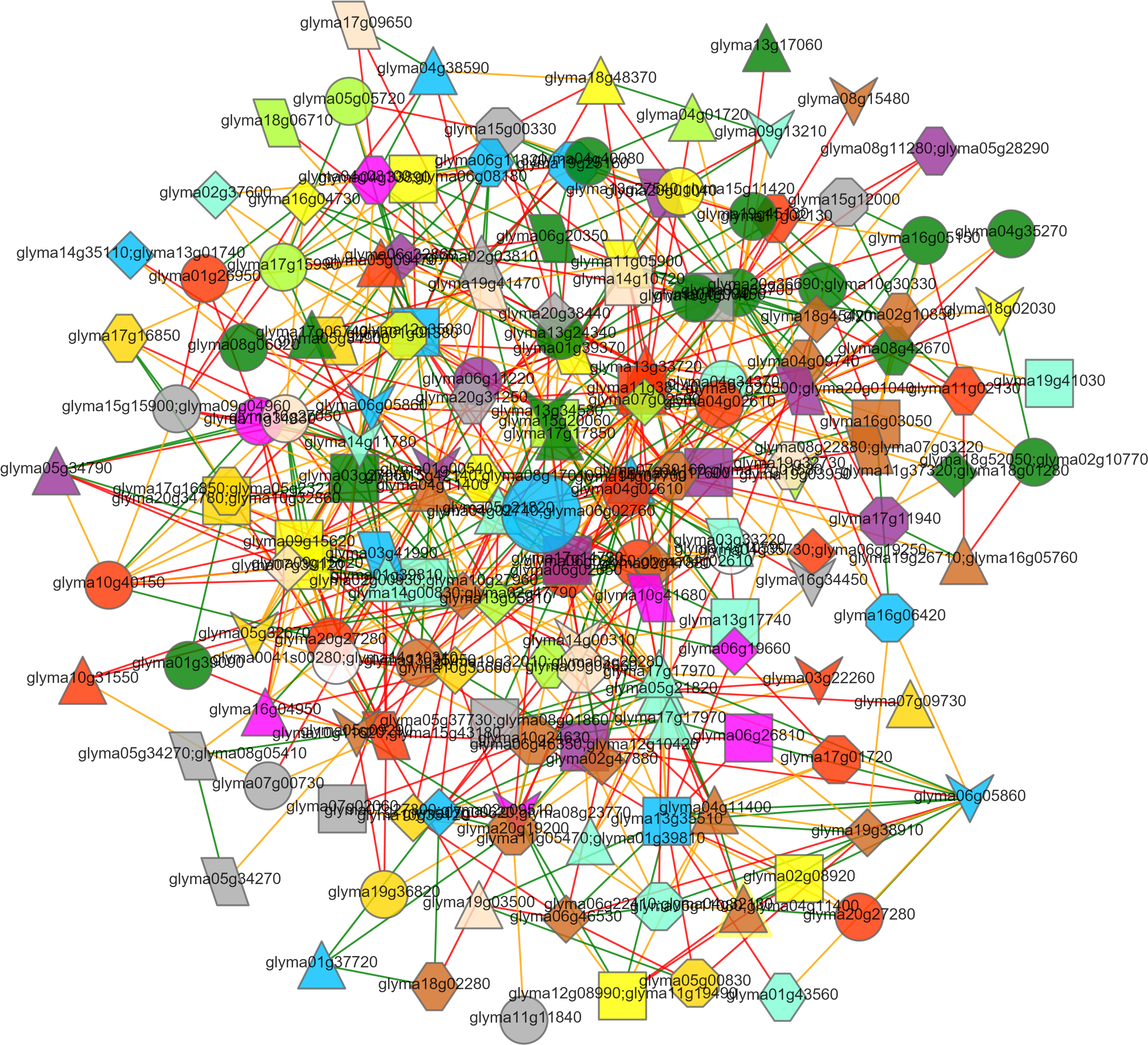
**

**
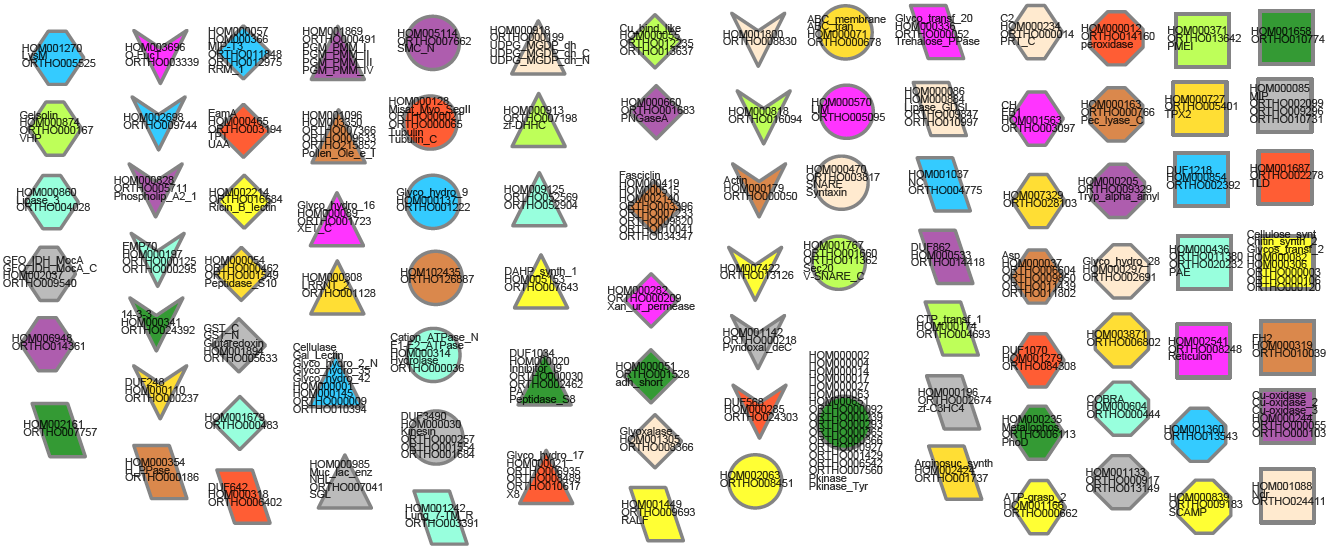
**

**D**

**
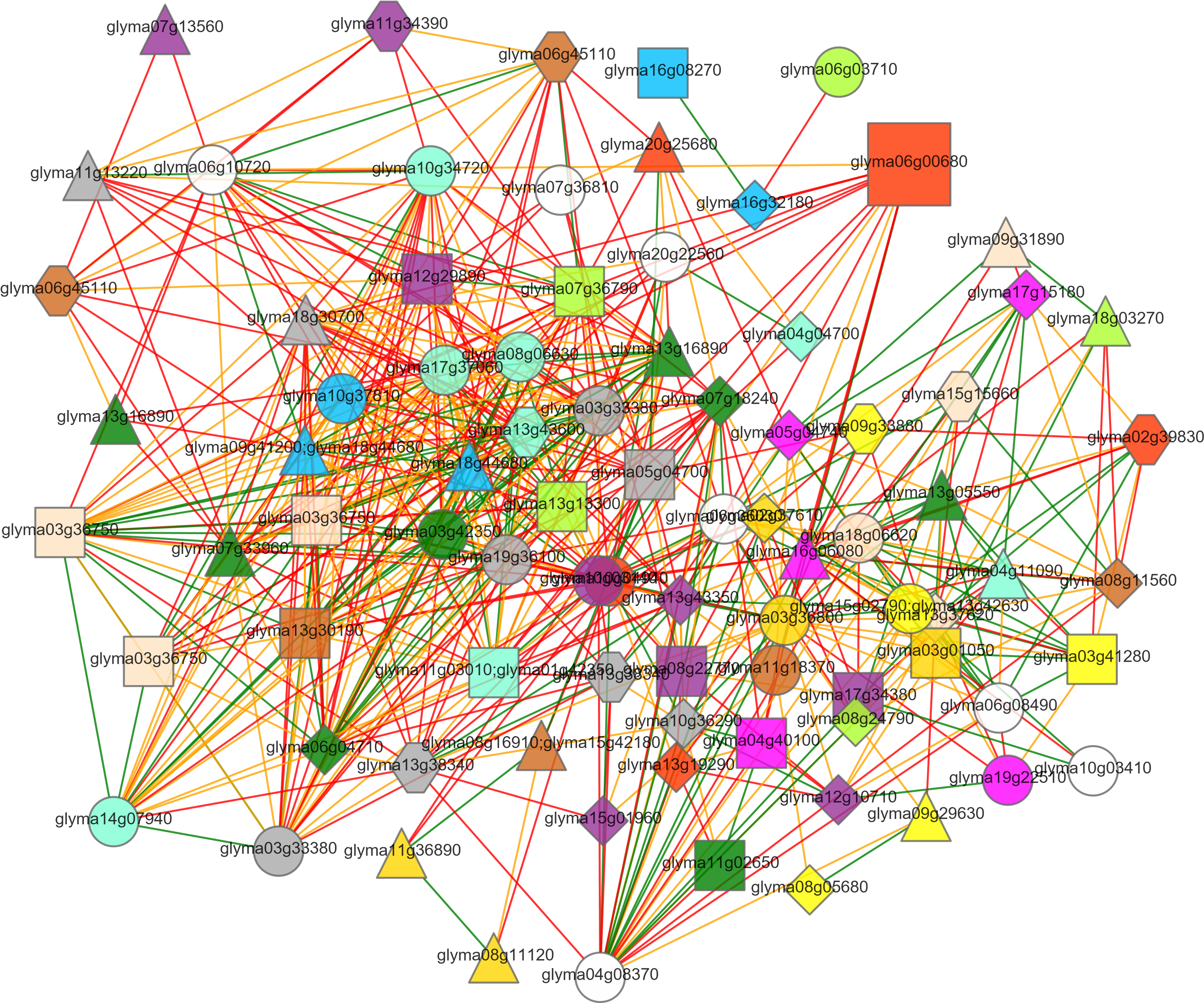
**

**
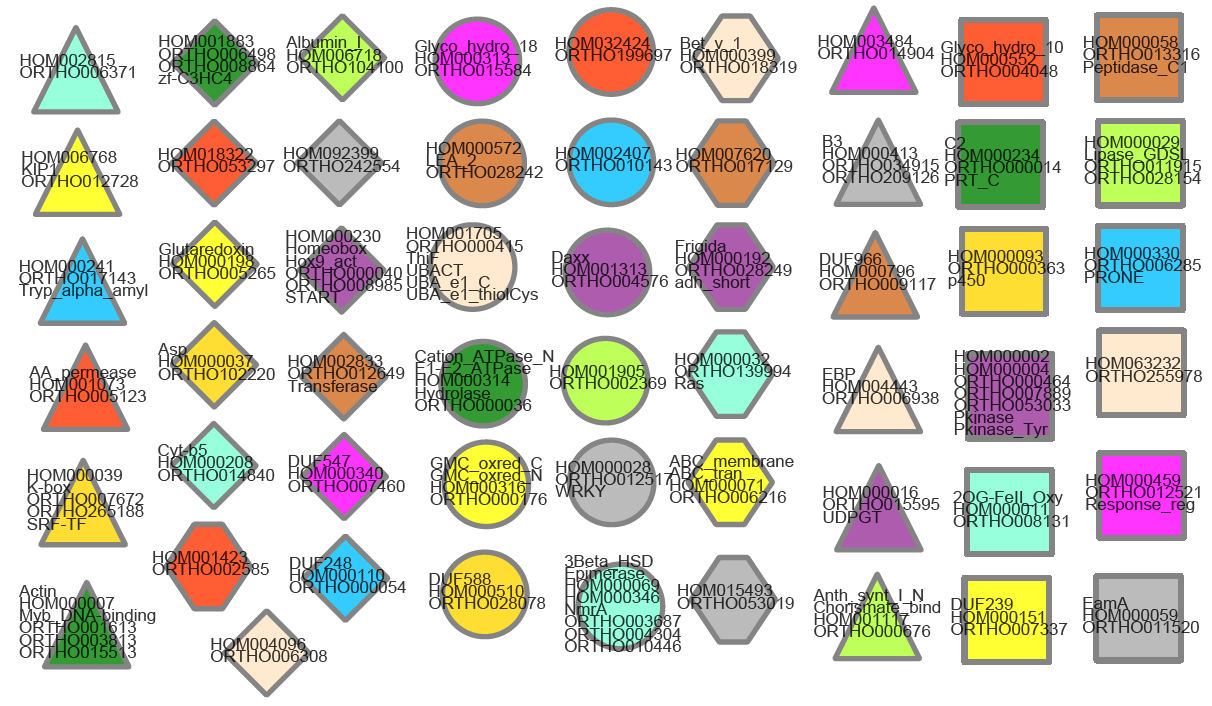
**

**E**

**
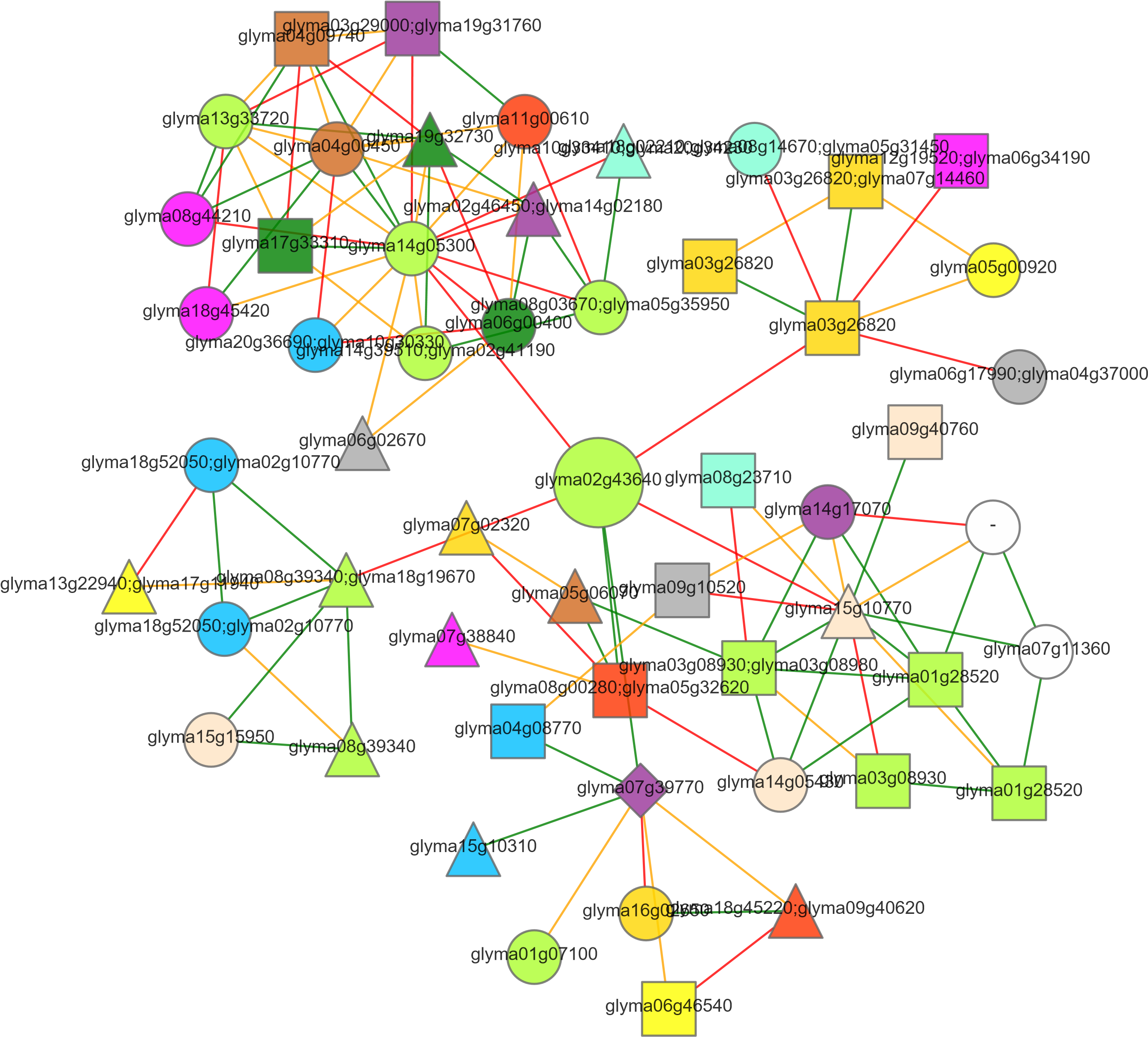
**

**
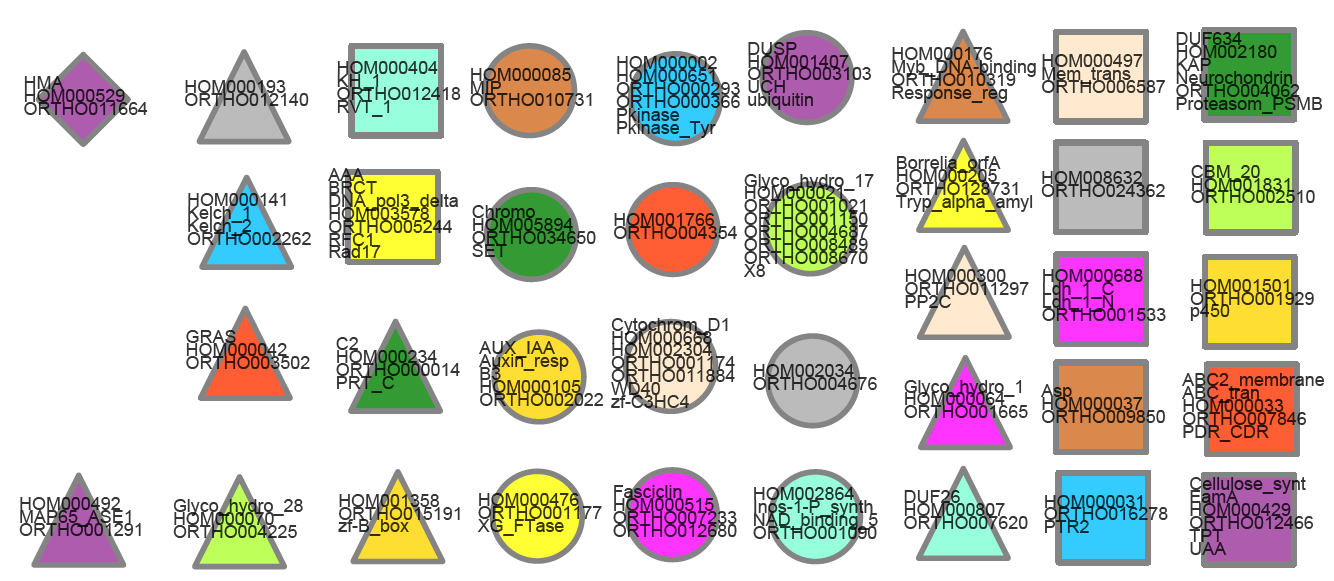
**

**F**

**
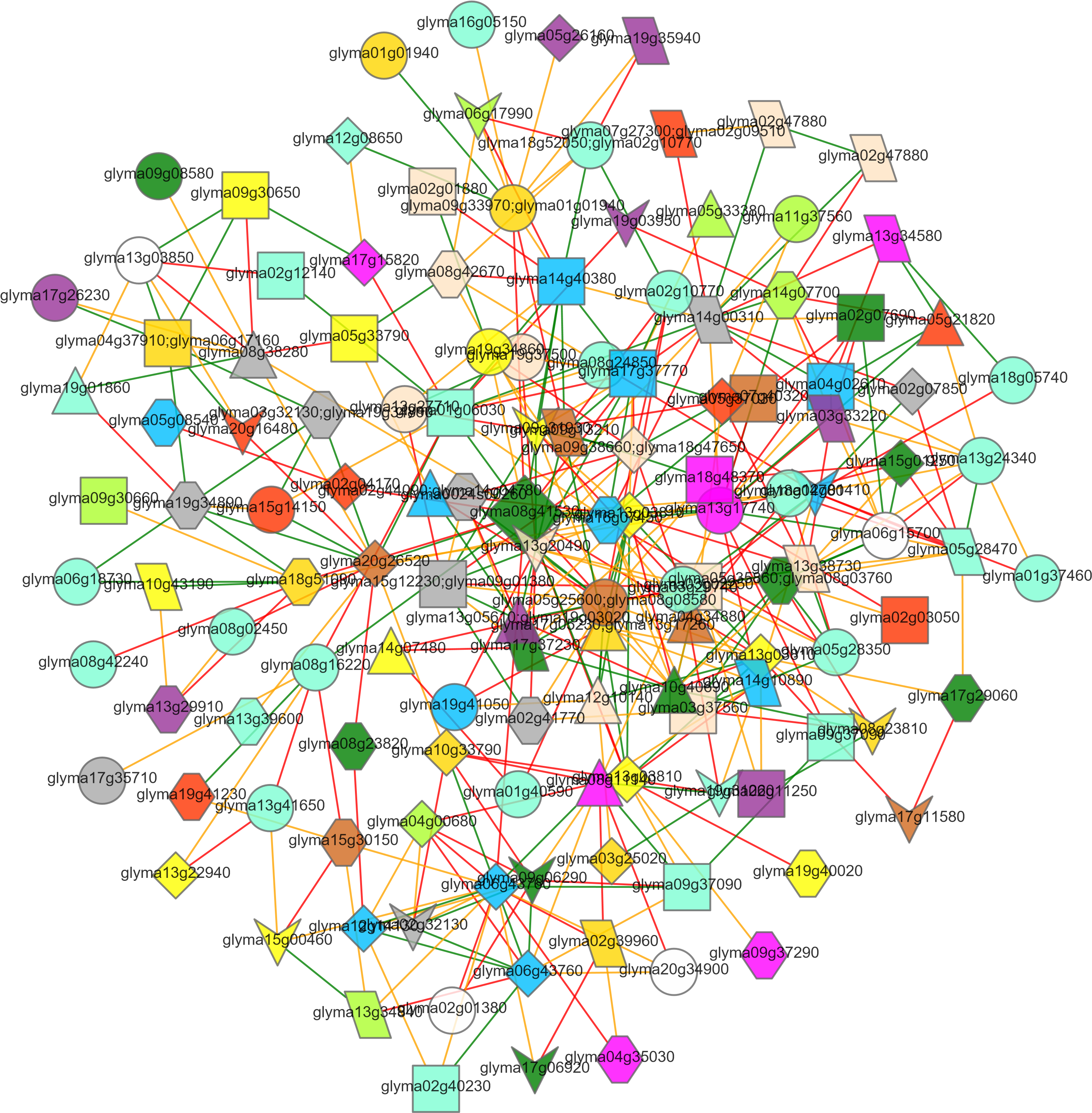
**

**
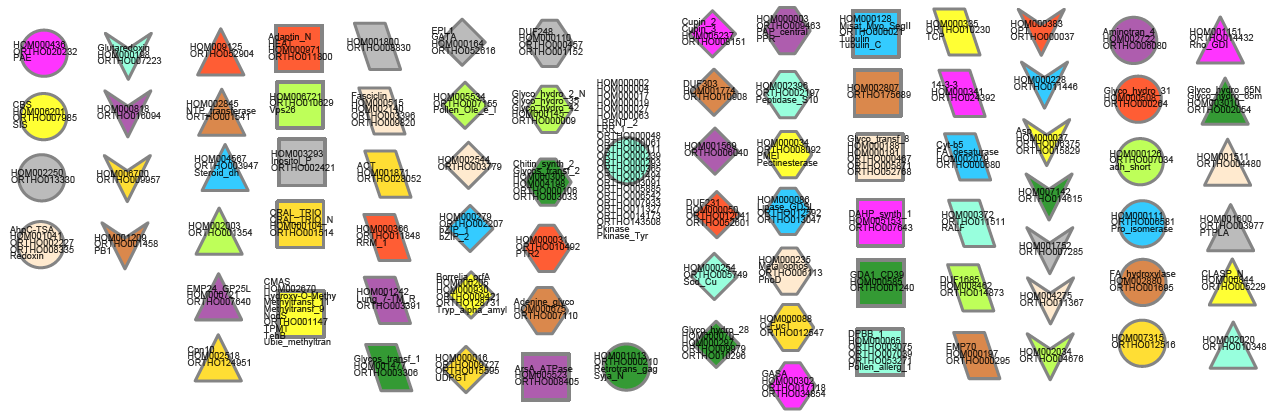
**

**G**

**
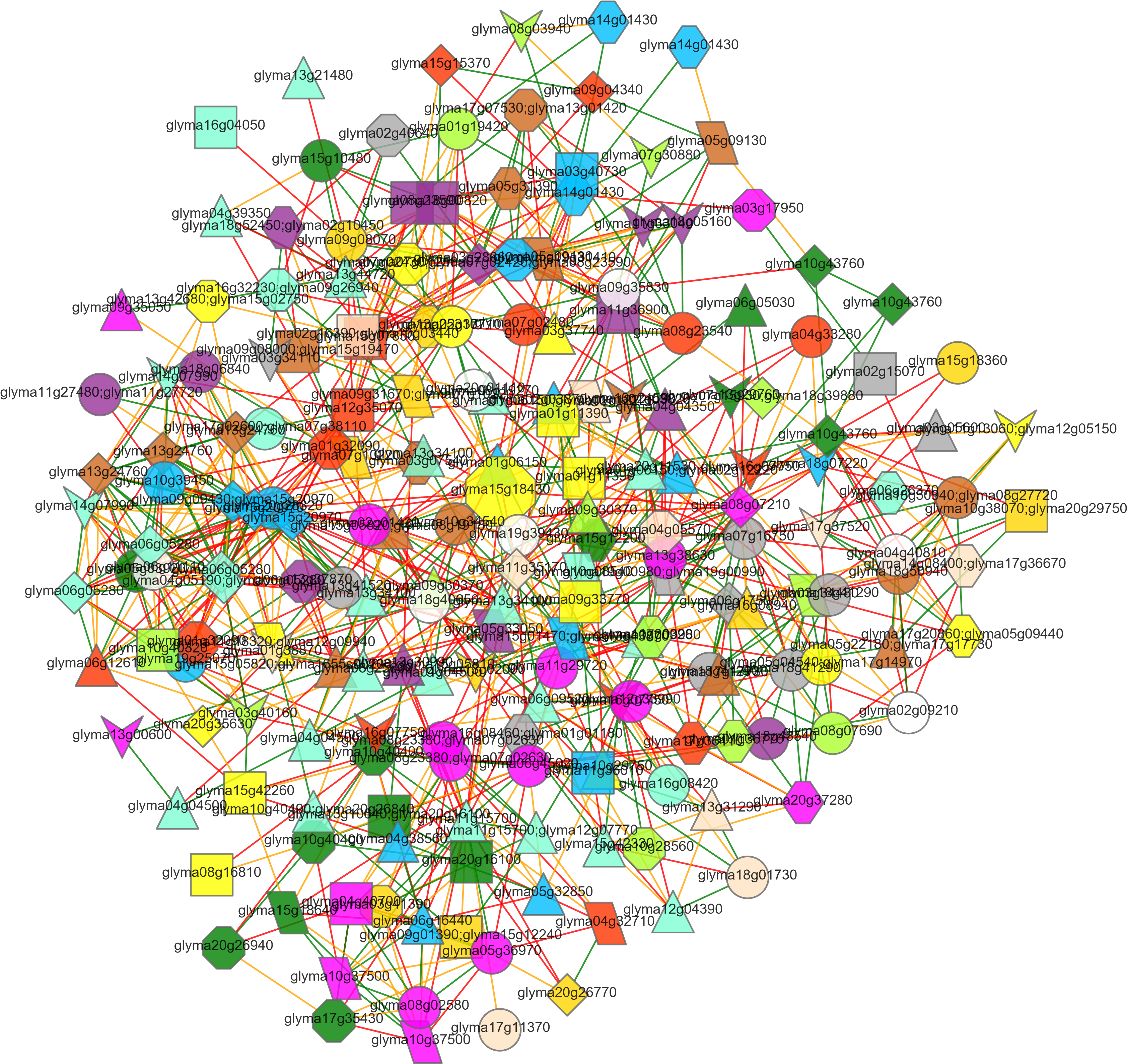
**

**
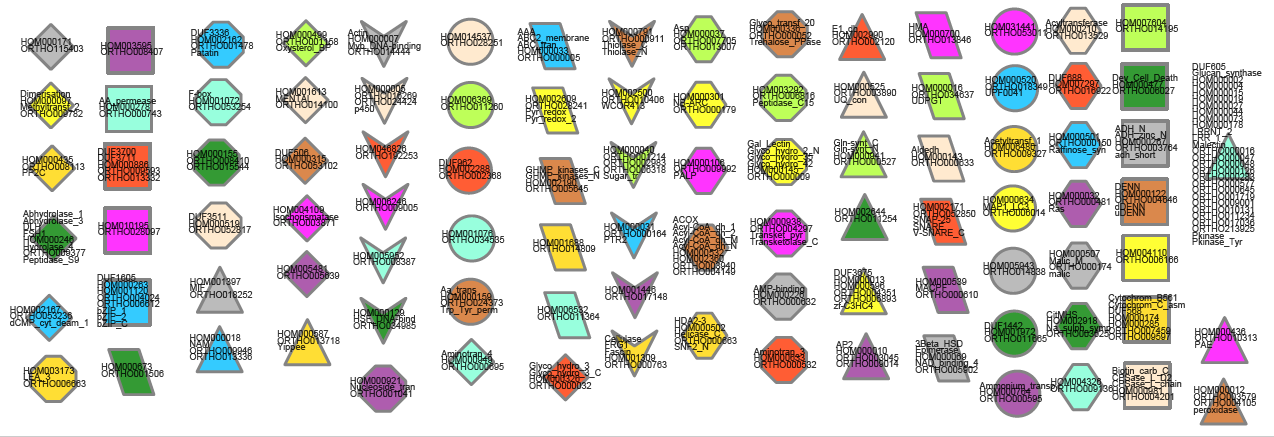
**

**H**

**
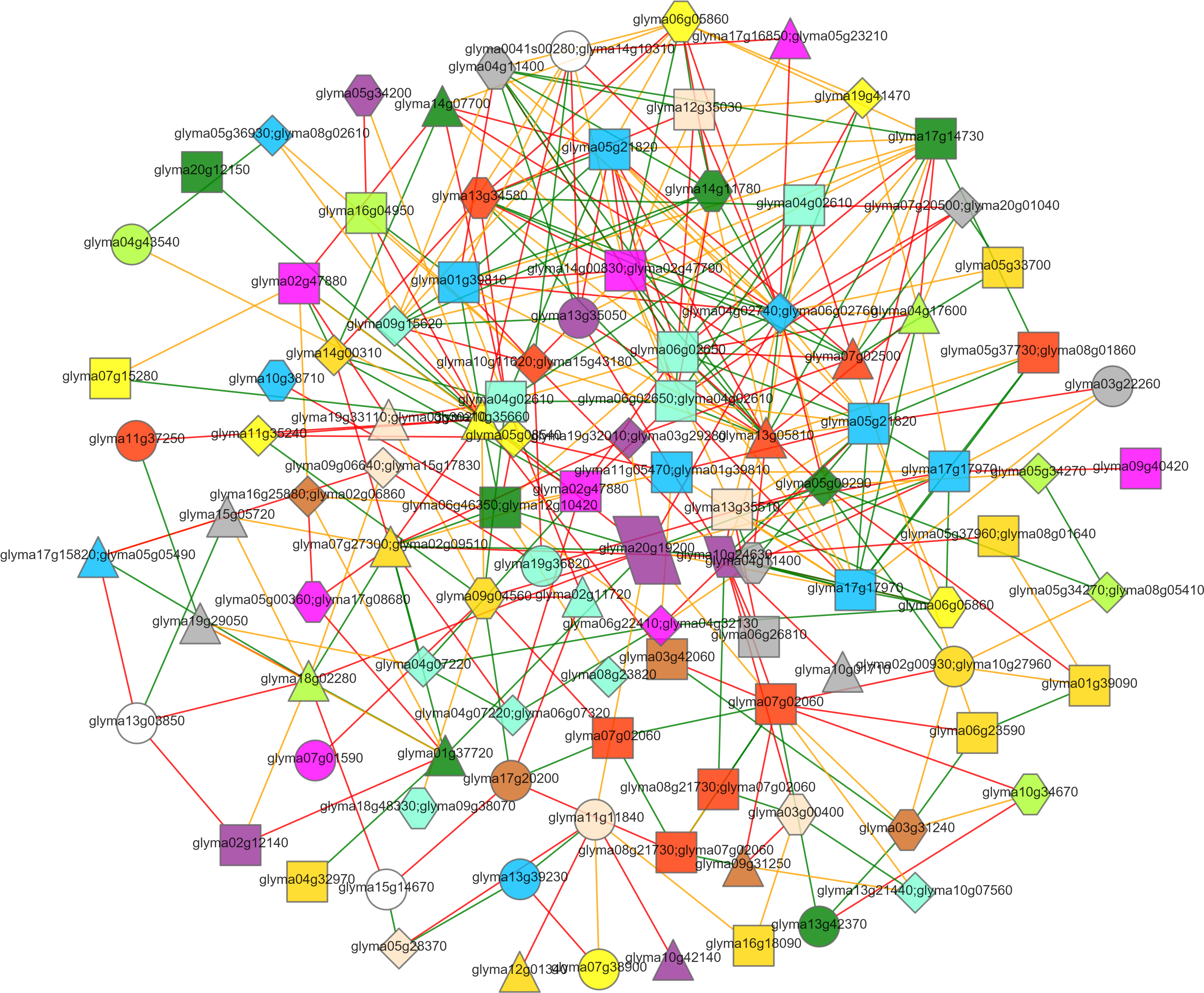
**

**
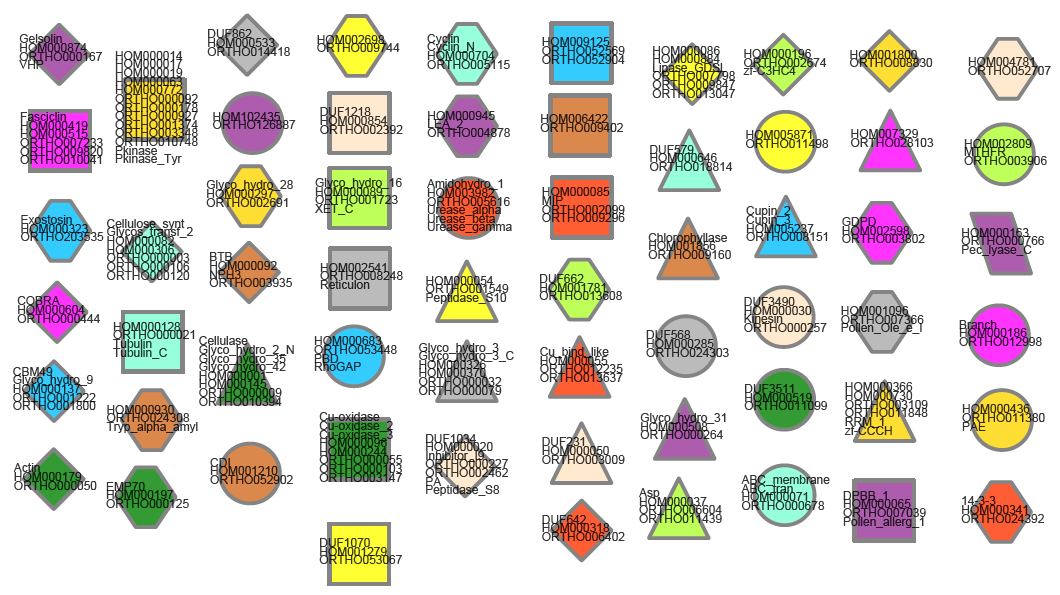
**

**I**

**
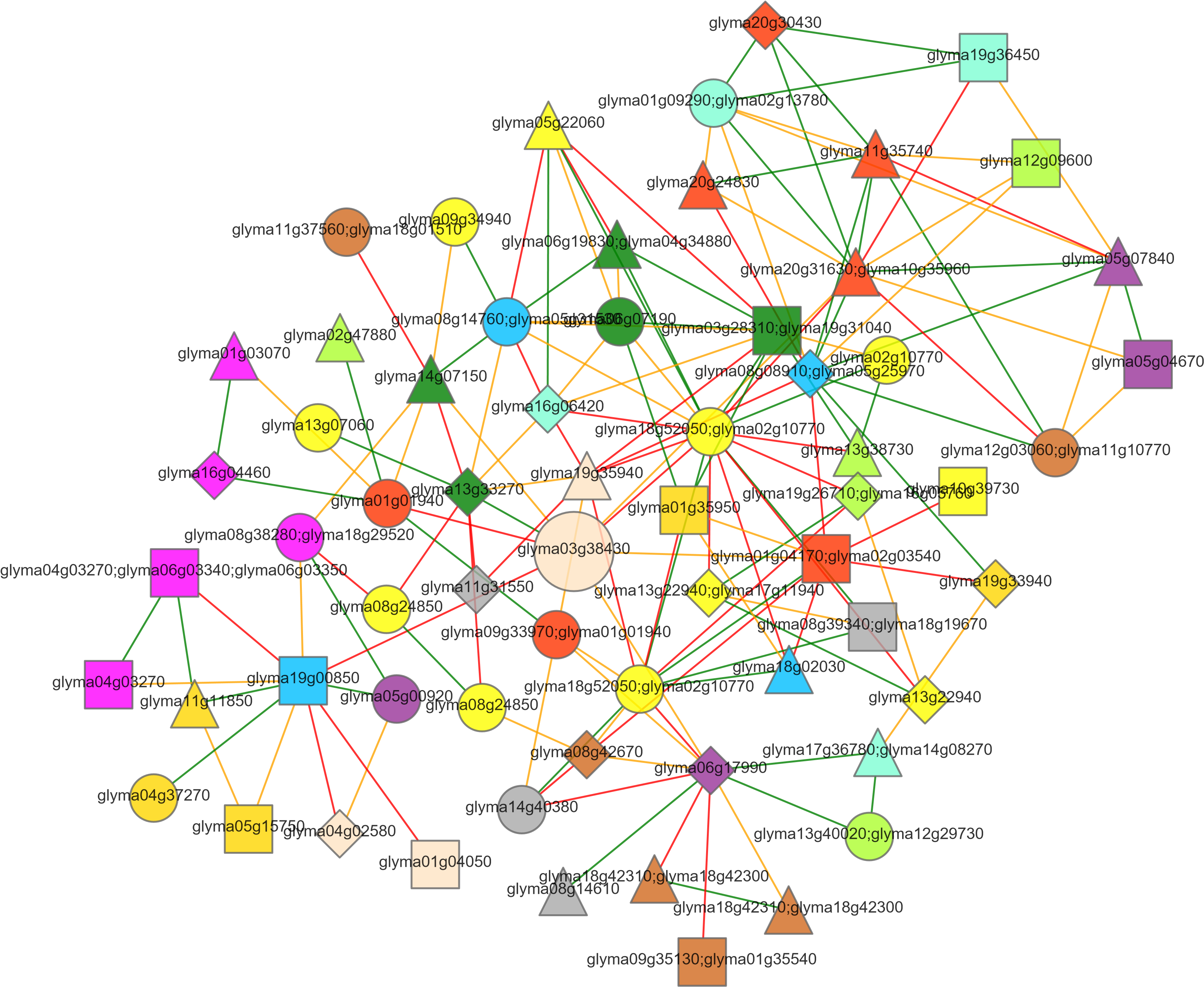
**

**
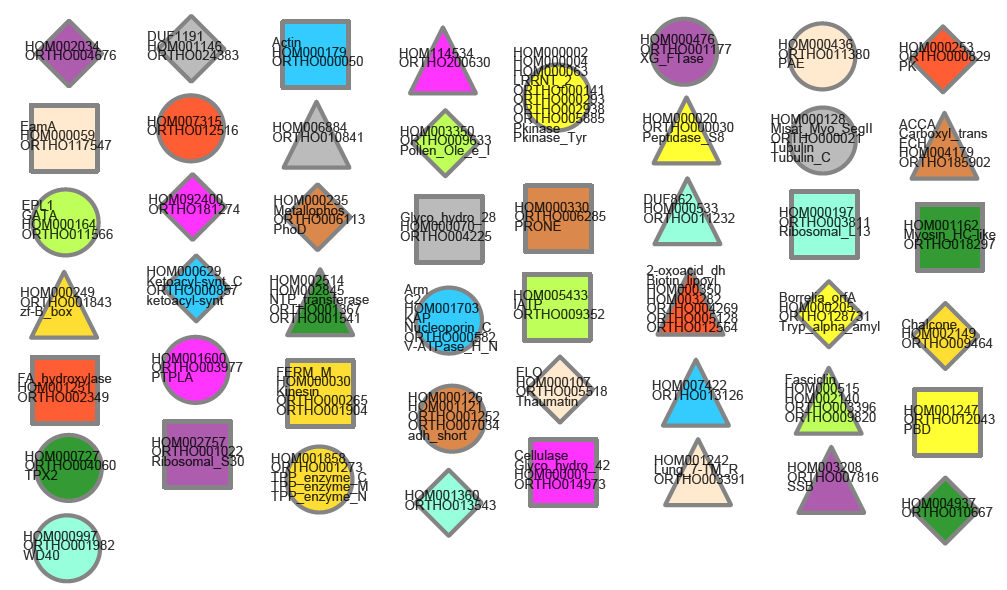
**

**J**

**
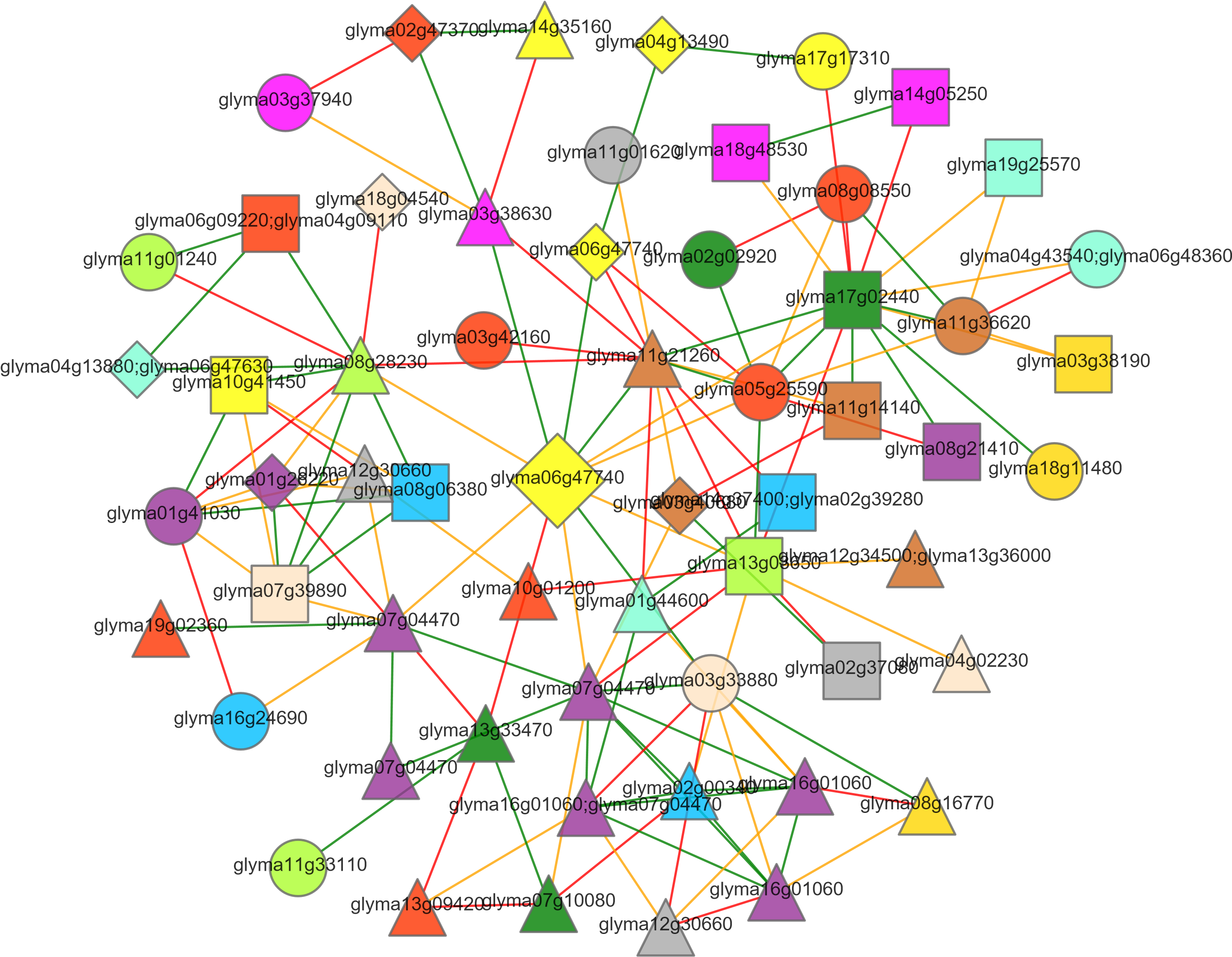
**

**
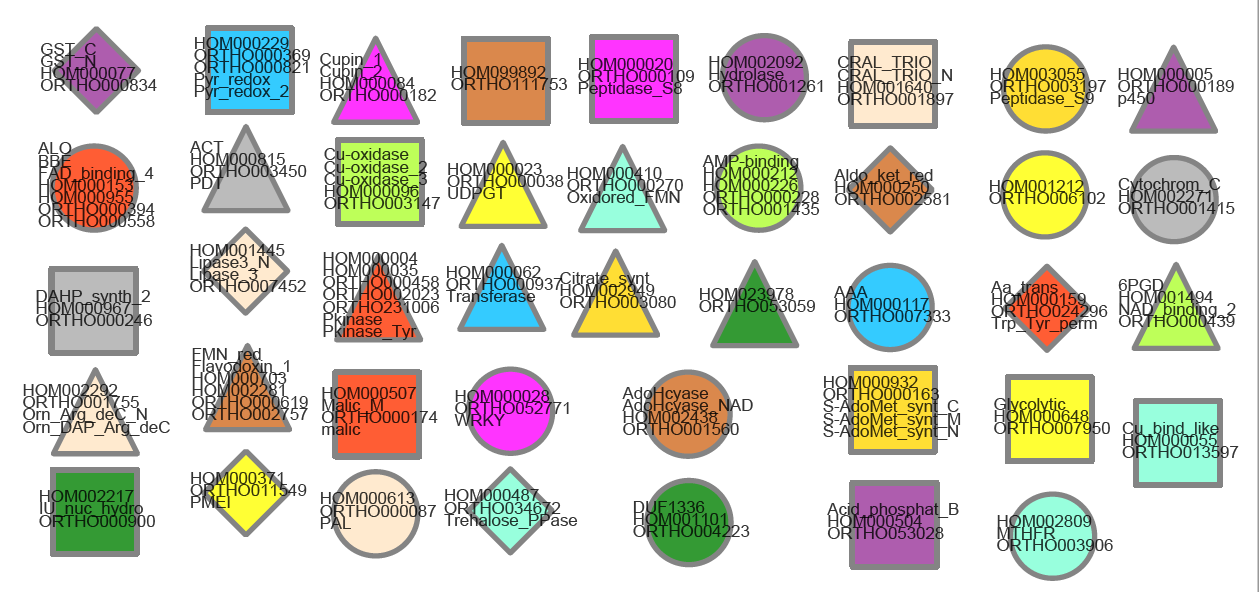
**

**K**
